# The replication initiator protein of a geminivirus interacts with host monoubiquitination machinery and stimulates transcription of the viral genome

**DOI:** 10.1371/journal.ppat.1006587

**Published:** 2017-08-31

**Authors:** Nirbhay Kumar Kushwaha, Mansi Bhardwaj, Supriya Chakraborty

**Affiliations:** Molecular Virology Laboratory, School of Life Sciences, Jawaharlal Nehru University, New Delhi, India; Zhejiang University, CHINA

## Abstract

Geminiviruses constitute a group of plant viruses, with a ssDNA genome, whose replication in the nucleus of an infected cell requires the function of geminivirus-encoded replication initiator protein (Rep). Our results suggest that monoubiquitinated histone 2B (H2B-ub) promotes tri-methylation of histone 3 at lysine 4 (H3-K4me3) on the promoter of Chilli leaf curl virus (ChiLCV). We isolated homologues of two major components of the monoubiquitination machinery: UBIQUITIN-CONJUGATING ENZYME2 (*NbUBC2)* and HISTONE MONOUBIQUITINATION1 (*NbHUB1*) from *N*. *benthamiana*. ChiLCV failed to cause disease in *NbUBC2*-, and *NbHUB1*-silenced plants, at the same time, H2B-ub and H3-K4me3 modifications were decreased, and the occupancy of RNA polymerase II on the viral promoter was reduced as well. In further investigations, Rep protein of ChiLCV was found to re-localize NbUBC2 from the cytoplasm to the nucleoplasm, like NbHUB1, the cognate partner of NbUBC2. Rep was observed to interact and co-localize with NbHUB1 and NbUBC2 in the nuclei of the infected cells. In summary, the current study reveals that the ChiLCV Rep protein binds the viral genome and interacts with NbUBC2 and NbHUB1 for the monoubiquitination of histone 2B that subsequently promotes trimethylation of histone 3 at lysine 4 on ChiLCV mini-chromosomes and enhances transcription of the viral genes.

## Author summary

Histone post translation modification plays vital role in regulation of gene expression in eukaryotes. In plants, Ubiquitin-Conjugating Enzyme 2 and Histone Monoubiquitination1 monoubiquitinates histone 2B that promotes trimethylation of histone H3 at lysine 4 and both modifications eventually activates host transcription. The chromatinized genome (viral DNA associated with host histone proteins) of geminiviruses serves as a potential template for histone modification enzymes. Our study reveals the role of Chilli leaf curl virus (*Geminiviridae*) encoded Replication initiator protein (Rep) in stimulation of viral transcription via histone modifications on the viral minichromosome. Furthermore, the Rep protein redirects NbUBC2 from cytoplasm to the nucleus to form monoubiquitination machinery in association with NbHUB1 and recruits the machinery to accelerate the H2B-ub deposition on the viral promoter. The silencing of *NbUBC2* and *NbHUB1* did not alter Rep localization in the nucleus but affect ChiLCV pathogenesis. Taken together, our results showed for the first time that geminivirus Rep protein recruits the NbUBC2 and NbHUB1 on the viral promoter for monoubiquitination of H2B and stimulates viral gene expression.

## Introduction

Geminiviruses, a group of single-stranded DNA (ssDNA) viruses have evolved a structurally simple yet elegant potential to interact with a wide range of plant proteins and manipulate cellular processes to create an ambience for the benefit of the virus. Geminiviruses infect several plant species and replicate in the nuclei of infected cells [1,2]. Using host proteins, the ssDNA genome of the geminiviruses is converted into double-stranded DNA that is further chromatinized in association with the host histones and forms a viral minichromosome [3], a potential template for transcription machineries. In eukaryotes, gene expression from a chromatinized DNA is initiated and regulated by a myriad number of post-translational modifications of histones that also regulate nucleosome dynamics [4]. In particular, many of these modifications directly or indirectly influence the recruitment of transcriptional regulatory factors to chromatin, providing an additional mechanism to control gene expression [5,6,7,8].

Viruses may target and utilize post-translational modifications of histones to modify and optimize the cellular environment for its own transcription [9,10]. For the efficient and orderly transcription of the viral genomic DNA to occur, the recruitment of the machinery responsible for histone post-translational modifications on the viral promoter is essential. Unlike RNA viruses, where either genomic or anti-genomic RNA serves as a transcript for the synthesis of virally encoded proteins, DNA viruses must necessarily undergo an additional step of transcription from genomic DNA to RNA to optimize and maintain the expression of the viral genome and to favour pathogenesis in a permissive host. For this, geminivirus genomes are organized in a manner that facilitates bidirectional transcription originating from the RNA polymerase II promoters embedded within the non-coding region (intergenic region; IR) near the origin of replication. The length of the IR varies among viruses (160 to 200 nt) and it encompasses all cis-acting elements required for DNA replication and transcription [2].

ChiLCV is a monopartite geminivirus (Family *Geminiviridae*, Genus *Begomovirus*) that accounts for major loss of chilli production in India and is predominant in the Indian subcontinent [11]. The ChiLCV genome consists of a single-stranded circular DNA (2750 nt) that is often associated with a betasatellite (1361 nt DNA) [11]. ChiLCV encodes for four open reading frames (ORFs), denoted as C1, C2, C3 and C4, in the complementary strand and two ORFs, denoted as V1 and V2, in the viral strand. The betasatellite depends on the helper virus genome and is transcribed to produce the βC1 protein, which functions as a pathogenesis determinant and suppressor of RNAi [12,13]. Of these proteins encoded by the geminivirus genome, replication initiator protein (Rep, encoded by ORF C1) is highly conserved both structurally and functionally across the *Geminiviridae* family [14,2,15]. Rep is a multitasking protein that is indispensable for the replication of the viral genome and confers virus-specific recognition of its cognate origin of replication [16,17,18]. Rep contains an N-terminal DNA-binding domain followed by an oligomerisation domain and a C-terminal helicase domain. The DNA-binding domain consists of motif I (DNA binding), motif II (metal binding), motif III (DNA cleavage and ligation) and a Geminivirus Rep Sequence (GRS) required for initiation of viral replication [14,15]. Rep interacts with and recruits several host factors to form the viral replisome [19,20] for rolling circle replication and interacts with proteins involved in the homologous recombination [21]. Rep can also reprogram plant cell cycles by disrupting the RBR-E2F complex by binding RBR [22,23,24]. Rep protein in conjunction with C4 protein represses methyltransferase (MET1) and chromomethylase 3 (CMT3) [25] to prevent methylation-dependent host RNAi surveillance. In addition, Rep can regulate its own transcription [26,27,16]. Rep has been shown to interact with small ubiquitin-related modifier (SUMO)-conjugating enzyme (SCE1) to presumably alter the SUMOylation level of host targets, thereby creating a suitable environment for virus replication [28,29]. Although, multitasking roles of Rep in replication have been elucidated, information about its role in the activation of viral gene expression is lacking. From the viral perspective, maintaining a steady level of viral transcript is the key to generate proteins that can facilitate pathogenesis and overcome host antiviral defense.

In one of the previous studies, the role of histone 3 (H3) in geminivirus movement has been demonstrated [30]. Recently, minichromosome of Pepper golden mosaic virus isolated from symptomatic pepper plants are found to be associated to a chromatin activator marker H3-K4me3 whereas minichromosome population obtained from a recovered tissues was associated to chromatin repressive marker H3-K9me2 [31]. Although, histone post translational modifications on viral minichromosome has been detected in the above case, the roles and mechanism of these modifications on geminivirus chromatin is still unknown. In one of our studies, histone 2B (H2B) transcripts were differentially expressed following ChiLCV infection, suggesting its role in viral pathogenesis. Histone 2B is one of the important components of the nucleosome and undergoes post-translational modification to regulate gene expression and nucleosome dynamics [32,33,34]. The role of H2B in geminivirus pathogenesis, however, is not yet known. Monoubiquitination of H2B has been reported to occur at specific location, such as at K120 in human [35], K123 in yeast [36,37,38] and K146 in the case of *Arabidopsis* [39]. In eukaryotes monoubiquitinated H2B (H2B-ub) marks chromatin that is transcriptionally active and serves as a precursor to epigenetic marks of transcriptional activation, including trimethylation of histone 3 at lysine 4 (H3-K4) and histone 3 at lysine 79 (H3-K79) by the COMPASS and DOT1 complexes [6].

Ubiquitin, a small 8.5 kDa protein with charged amino acid residues on its surface that facilitate protein-protein interactions, alters the molecular conformation of the target protein, hence promoting its interaction with other proteins. In *Arabidopsis*, monoubiquitination of H2B is achieved by the homolog of Rad6 ubiquitin conjugating enzyme (UBC1 and UBC2) and BRE1 homolog of histone monoubiquitination E3 (HUB1 and HUB2) enzymes [40,32,41]. In plants, monoubiquitination of H2B has been reported to regulate the expression of key genes involved in leaf and root growth and seed dormancy [40,32], as well as flowering time and plant development [39,42,41]. Recently, H2B-ub-mediated regulation of gene expression of photomorphogenesis and the circadian clock has also been documented [43,44]. Furthermore, the involvement of HUB1 as a regulatory component of plant defense against necrotrophic fungal pathogens [45] suggested a multifunctional role of HUB1 in plant developmental processes and responses to biotic stress.

The chromatinized DNA of geminiviruses acts as a potential substrate for the enzymes involved in cellular histones post-translational modification machineries, RNA polymerase II and other transcription activator proteins. Recruitment of these machineries and the fate of histone modification on the geminivirus minichromosome for regulation of viral gene expression are not known at present. Here we show that H2B-Ub is deposited on the viral promoter and marks chromatin regions with H3-K4me3. Altogether our results indicate that Rep protein influences the epigenetic control of ChiLCV by modulating the recruitment of chromatin-modifying enzymes onto the geminiviral minichromosome.

## Results

### Deposition of H2B-ub and tri-methylated H3-K4 on the ChiLCV genome

**Fig 1:**
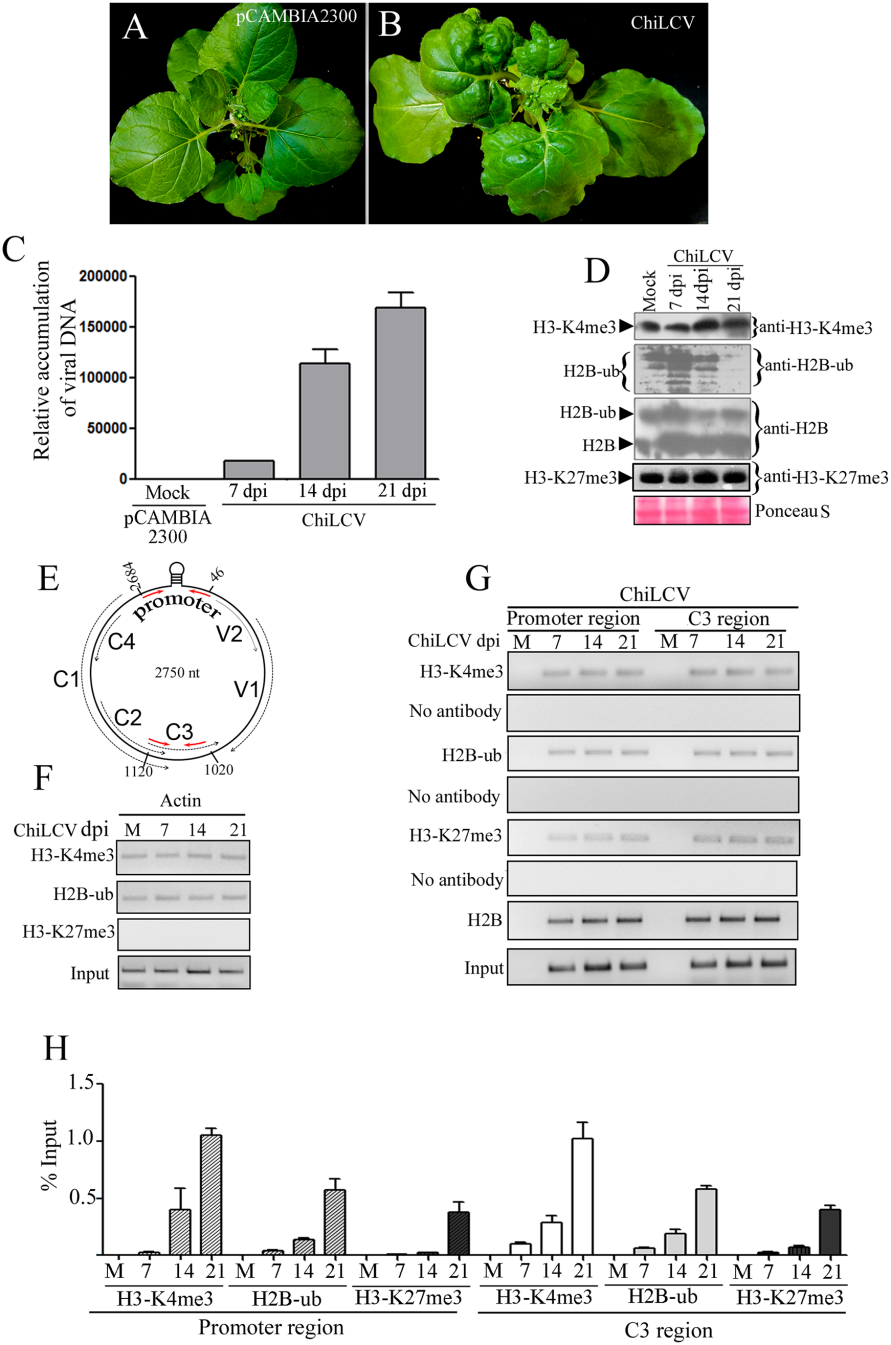
Deposition of H2B-ub and H3-K4me3 on the ChiLCV genome. **(A)** An *N*. *benthamiana* plant inoculated with the vector pCAMBIA2300. (B) Phenotype of a representative *N*. *benthamiana* plant inoculated with ChiLCV showing typical symptoms of leaf curl disease at 21 days post inoculation (dpi). (C) qPCR of viral DNA accumulation at different dpi. (D) Immunoblot analysis of global cellular H2B, H2B-ub, H3-K4me3 and H3-K27me3 levels in mock-, and virus-inoculated *N*. *benthamiana* plants at 7, 14 and 21 dpi. Immunoblotting was performed using anti-H3-K4me3, anti-H2B-ub, anti-H2B and anti-H3-K27me3 specific antibodies following standard protocol. (E) Schematic diagram of the circular genome of ChiLCV (2750 nt) indicating the relative positions of the viral ORFs and the position of the primers (red arrows) used for chromatin immunoprecipitation in the study. (F) Detection of occupancy of H3-K4me3, H2B-ub and H3-K27me3 on the *Actin* genic region by ChIP-PCR serves as control. (G-H) Detection of H3-K4me3, H2B-ub, H3-K27me3 and H2B on the promoter and C3 region of ChiLCV by ChIP-PCR at different time points following infection using anti-H2B, anti-H2B-ub, anti-H3-K4me3 and H3-K27me3 antibodies and primers specific to the ChiLCV promoter (2684–46 nt) and the C3 region (1020–1120 nt).

*N*. *benthamiana* plants inoculated with a tandem repeat of the ChiLCV genome (DNA-A-like) and betasatellite (DNA β) manifested severe leaf curl disease. Mock inoculated plants did not exhibit any morphological changes (Fig 1A). ChiLCV induced symptoms on *N*. *benthamiana* such as downward leaf curling, vein swelling, shorter internodes, and stunted plant growth with reduced flowering (Fig 1B). Susceptibility to ChiLCV was also determined by evaluating the viral DNA and transcript in the inoculated plants. The viral DNA and transcript levels increased with the progression of disease from 7 to 21 days post inoculation (dpi) (Fig 1C and S1A Fig). A previous study suggested that a replicative dsDNA form of a geminivirus genome assembled into 11–12 nucleosome-like structures to form a minichromosome using host histone proteins [46]. Since eukaryotic chromatin is a substrate for several post-translational machineries that regulate gene expression, we hypothesized that a geminivirus minichromosome could also serve as a precursor for histone post-translational modifications machineries for active and efficient expression of the viral genome. In eukaryotes, monoubiquitinated H2B (H2B-ub) promotes trimethylation of histone 3 at lysine position 4 (H3-K4me3). Together, these two histone modifications are predominantly associated with transcriptionally active chromatin, and are considered as a hallmark of active transcription. Firstly, we analyzed the global cellular level of both H2B-ub and H3-K4me3 in mock- and ChiLCV-inoculated plants at 7, 14 and 21 dpi. We isolated total histone proteins from the mock-, and virus-inoculated plants and performed immunoblotting with anti-H2B, anti-H2B-ub and anti-H3-K4me3 specific antibodies. The global cellular levels of the H3-K4me3 modifications in mock- and virus-inoculated plants did not indicate significant alterations during the course of the investigation (until 21 dpi) (Fig 1D). The H2B-ub level was increased at 7 dpi but further declined at 14 and 21 dpi of ChiLCV infection. Immunoblotting of total histone proteins with anti-H2B specific antibodies showed two distinct sizes of H2B, one of which is consistent with unmodified H2B and the other corresponds to ubiquitinated H2B (Fig 1D). As found earlier, an enhanced level of H2B-ub was detected at 7 dpi followed by a decrease at 14 and 21 dpi (Fig 1D). We also investigated the level of H3-K27me3, which is one of the signatures of repression of gene expression in eukaryotes. The global level of H3-K27me3 modifications remains low as compared to H3-K4me3 and H2B-ub during the course of experiments (7–21 dpi) (Fig 1D).

Next, we carried out ChIP-PCR to investigate the association of H3-K4me3, H2B-ub, H3-K27me3 and H2B with the viral genome. *NbActin* gene was taken as control for the ChIP experiments. The gene for actin is a house-keeping gene and, therefore, it is expected to be under active transcription through histone post-translational modifications. We detected deposition of H2B-ub and H3-K4me3 on actin in both mock- and ChiLCV-inoculated plants that served as controls (Fig 1F). However, H3-K27me3 occupancy could not be detected on the *NbActin* gene (Fig 1F). Furthermore, deposition of H2B-ub and H3-K4me3 were also examined on the viral promoter and the exon (C3) region by chromatin immunoprecipitation assays using specific antibodies and ChiLCV-specific primers (Fig 1G and 1H). ChIP experiments were conducted on the inoculated plants at different time points (7–21 dpi), whereas corresponding vector-inoculated plants were considered as mock plants. Amplification of viral and actin from sonicated samples were considered as input controls that showed DNA (Fig 1F–1G). ChIP-PCR using viral promoter and genic region primers of the mock-inoculated plants did not show amplification (Fig 1G and 1H). ChIP-PCR results showed deposition of H2B-ub both on the ChiLCV promoter and C3 region of viral genome (Fig 1G and 1H). The occupancy of H3-K4me3 and H2B-ub increased from 7–21 dpi with viral titre (Fig 1H). H3-K27me3 deposition was also detected on the viral promoter and genic region, but the level was considerably lesser than that of the H3-K4me3 and H2B-ub deposition. ChIP-PCR was carried out using anti-H2B specific antibody as control of unmodified histone. The result showed increasing association of H2B with viral genome during the course of study. ChIP-PCR of mock-, and virus-inoculated plants was performed likewise except for the addition of antibody. These samples served as negative controls to indicate the specificity of the antibodies used in the experiments. Samples treated without antibody did not show amplification in ChIP-PCR (Fig 1G). ChIP-PCR from mock-, and virus-inoculated plants using anti-GFP specific antibody indicates specificity of the experiments performed using antibodies used for detection of histone post translational modifications on viral genome (S1B Fig).

### Cloning of *NbHUB1* and *NbUBC2* and their expression profile

*NbUBC2* and *NbHUB1* were amplified, sequenced and annotated on the NCBI database (www.ncbi.nlm.nih.gov). *NbUBC2* (462 nt; GenBank accession no. KU726872) showed 97% nucleotide identity with *N*. *sylvestris* and the amino acid sequences shared 99% identity with most of the solanaceous plants including *S*. *lycopersicum*, *N*. *sylvestris*, *N*. *tabacum*, *S*. *tuberosum* (S1 Table). We extracted the protein sequence of UBC2 from various plant species and performed a CLUSTALW analysis using MEGA6 software (S1 Text). The NbUBC2 protein sequence indicated the presence of 152 highly conserved amino acid residues (S1 Text). Furthermore, the phylogenetic tree of NbUBC2 showed close homology with *N*. *tabacum* (S2A Fig). To determine the locus structure, we applied BLAST to the *NbUBC2* sequence on the *N*. *benthamiana* genome sequence available on the Sol Genomics Network database (https://solgenomics.net). *NbUBC2* with 462 nts was observed to span from nt number 319, 901 to nt number 323,600 (Niben101Scf02253) (S2C Fig) and the *NbUBC2* was found to be organized into five exons and four introns (S2C Fig). We also used the NbUBC2 protein sequence to search for any similar domains on the CDD NCBI database and it was observed that NbUBC2 was similar to residues 1–150 aa of the ubiquitin-conjugating enzyme domain (S2C Fig).

Similarly, we isolated *HUB1* from *N*. *benthamiana*, and this *NbHUB1* (GenBank accession no. KU726871) was observed to share 99% nucleotide sequence identity with that of *S*. *lycopersicum*. We performed a protein BLAST of the *NbHUB1* sequence, and this run of BLAST showed a 100% similarity of the NbHUB1 sequence with HUB1 of *S*. *lycopersicum* and a 97% similarity with that of *S*. *tuberosum* (S1 Table). The amino acid sequences of HUB1 were found to vary among the solanaceous plants. For example, the sequence similarity between NbHUB1 and HUB1 of *N*. *sylvestris* was found to be 88% (S1 Table). We extracted the HUB1 sequences from the NCBI database and performed multiple alignment using MEGA6 software. Unlike NbUBC2 described above, HUB1 sequences were highly variable among the different plant species (S2 Text). The phylogenetic tree of *NbHUB1* indicated a close homology of *NbHUB1* with *HUB1* of *S*. *lycopersicum*, *N*. *sylvestris*, and *S*. *tuberosum* (S2B Fig). The *NbHUB1* gene size was mapped on *N*. *benthamiana* using the Sol Genomic Network database information. The *NbHUB1* CDS sequence (with a length of 2544 nt) was spread over 14,279 bp on the *N*. *benthamiana* genome (Niben044Scf00014408Ctg007) but segregated in 21 exons (S2D Fig). Furthermore, we analyzed the conserved domain in the NbHUB1 protein sequence using the conserved domain database (CDD) of NCBI. This analysis indicated that NbHUB1 contains a low-complexity region (97-110aa), three alpha-helical coiled-coil structures (201-223aa, 581-624aa, 771-809aa) and a conserved RING domain of E3 ligases (826-847aa) (S2D Fig).

Further, we used sequences of the major components of the monoubiquitination machinery such as NbHUB1, NbUBC2 and NbH2B to generate a protein interactome network on the STRING database that used *Arabidopsis* information. The interactome results suggested an interaction of H2B with HUB1 and UBC2. Furthermore, the results also suggested an interaction between NbHUB1 and NbUBC2 (S2E Fig).

We carried out qRT-PCR to check the expression of *NbH2B* (Genbank accession no. KU726873), *NbHUB1* and *NbUBC2* in different parts of the plant. The root and stem showed comparable levels of accumulation of H2B transcripts, whereas *NbH2B* expression was lower in the leaves sample and least in the flowers (S2F Fig). *NbUBC2* expression was found to be higher in the leaf and root tissues than in the stem and flower (S2G Fig). The *NbHUB1* transcript accumulation was found to be minimum in the flowers, and the maximum in the root tissues (S2H Fig). Leaf samples showed a greater than two-fold higher level of *NbHUB1* expression than did the stems (S2H Fig).

Furthermore, we investigated expression of *NbUBC2*, *NbHUB1* and *NbH2B* transcripts in ChiLCV-inoculated *N*. *benthamiana* plants. *NbH2B* transcripts showed different expression pattern in the ChiLCV-inoculated plants than did *NbUBC2* and *NbHUB1* (S2I Fig). *NbH2B* expression was very low in mock-inoculated plants (21 dpi), but viral infection raised the NbH2B level. NbH2B level was found to be increased from 7 to14 dpi then reduced at 21 dpi (S2I Fig). *NbUBC2* expression level was the same in mock and 7 dpi plants, whereas 14 and 21 dpi plants showed almost equal but reduced levels of *NbUBC2* transcripts (S2J Fig). *NbHUB1* transcript expression was reduced following ChiLCV infection at 7 dpi, and further increased at 14 dpi (S2K Fig). Noticeably, *NbHUB1* expression was again reduced at 21 dpi in *N*. *benthamiana* (S2K Fig).

### Silencing of *NbHUB1* and *NbUBC2* affected ChiLCV-mediated disease development

**Fig 2:**
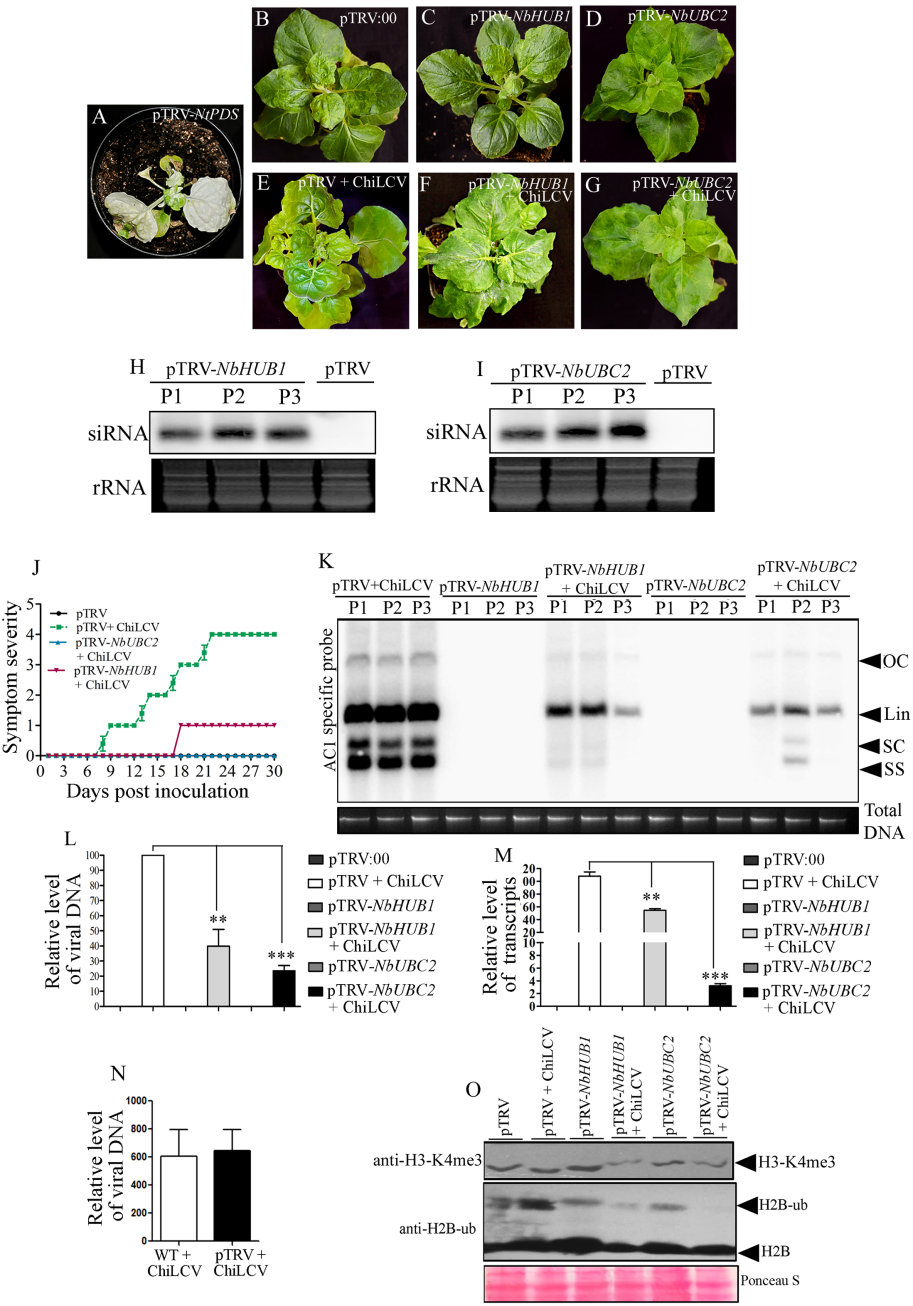
Silencing of *NbUBC2* and *NbHUB1* affected ChiLCV pathogenesis and deposition of H2B-ub and H3-K4me3 on the viral genome. (A-G) Phenotype of *N*. *benthamiana* plants infiltrated with pTRV-*NtPDS*, empty vector (pTRV: 00), with or without ChiLCV inoculated, pTRV-*NbUBC2*, pTRV-*NbHUB1* plants at 21 dpi. (H) Detection of *NbHUB1* specific siRNAs in three independent silenced plants (P1, P2, P3) of *N*. *benthamiana*. (I) Detection of *NbUBC2* specific siRNA in three different silenced plants of *N*. *benthamiana*. (J) Symptom severity graph showing disease development of ChiLCV on pTRV:00, *NbUBC2-*, and *NbHUB1*-silenced *N*. *benthamiana* plants. (K-L) Relative level of ChiLCV DNA accumulation in either mock-, or virus-inoculated silenced plants by Southern blot and qPCR assays. Total plant DNA loaded is indicated below the blot (K). The statistical significance between the mean values was calculated by *t*-test (***p<0.001, **p<0.01). (M) Expression analysis of C2 transcripts level in pTRV:00, *NBUBC2*-and*NbHUB1*-silenced plants inoculated with ChiLCV (21 dpi). The statistical significance of the differences between the mean values were calculated by performing *t*-test (***p<0.001, **p<0.01). (N) An analysis of the viral titer in the absence (WT) or in the presence of pTRV infiltrated *N*. *benthamiana* plants inoculated with ChiLCV at 21 dpi. (O) Detection of H3-K4me3 and H2B-ub in *NbUBC2*, and *NbHUB1* silenced *N*. *benthamiana* plants by immunoblotting assays.

We used a TRV virus-based VIGS approach to study the roles of *NbUBC2* and *NbHUB1* in ChiLCV pathogenesis in the *N*. *benthamiana* plants (Table 1). Although VIGS is a transient assay but it is a highly effective reverse genetics tool to study functional characterization of gene(s) of interest. This methodology has successfully been used for the functional characterization of several genes in plants [47,48,49,50,51]. We infiltrated *N*. *benthamiana* plants with equal amount mixture of *Agrobacterium* containing pTRV1 and pTRV2-*NtPDS* to test the silencing efficiency. PDS silencing was manifested on the silenced plants as photobleaching, which appeared on the newly emerging leaves as early as 8 dpi and showed prominent chlorosis throughout the leaves at 21 dpi (Fig 2A). Similarly, a pTRV-based VIGS vector was constructed to silence *NbUBC2* and *NbHUB1* genes in *N*. *benthamiana* (Table 1). Agroinfiltration was performed with a pTRV vector containing a fragment of either the *NbUBC2* or the *NbHUB1* gene sequence in an inverted orientation. Empty vector pTRV:00 (pTRV1+pTRV2)-inoculated plants did not show symptom or phenotypic changes during the course of experiments (Fig 2B). *NbHUB1*-silenced plants did not show phenotypic changes relative to vector-inoculated mock plants (Fig 2C). Also, we did not notice any morphological abnormalities on *N*. *benthamiana* plants infiltrated with pTRV2*-NbUBC2* (Fig 2D). *NbUBC2* and *NbHUB1* silencing was confirmed by detection of *NbUBC2-*, and *NbHUB1*-specific siRNAs present in the silenced plants (Fig 2H and 2I).

**Table 1:** Effect of *NbUBC2-* and *NbHUB1-* silencing on *ChiLCV* pathogenesis.

Constructs	No of plants performed VIGS	Abnormalities	No of plants inoculated with ChiLCV	No of plants showing symptom	Symptoms*	Symptom severity#
pTRV1 + pTRV2	50	no	-	-	no	-
pTRV1+ pTRV2-*NtPDS*	9	Photo bleaching	-	-	no	-
pTRV1 + pTRV2-*NbUBC2*	50	no	-	-	no	-
pTRV1 + pTRV2-*NbHUB1*	50	no	-	-	no	-
pTRV1 + pTRV2 + pCAMBIA2300	50	no	0	0	no	0
pTRV1+pTRV2+ChiLCV	-	-	25	25	LC,VT, St, LD, Tw	4
pTRV1 + pTRV2-*NbUBC2* + ChiLCV	50	no	30	0	no	0
pTRV1 + pTRV2-*NbHUB1* + ChiLCV	50	no	30	26	Mild LC	1

^#^Symptom severity was assessed according to Chakraborty et al. [11]

*LC—leaf curling, VT—vein thickening, St—stunting, LD—leaf distortion, Tw—twisting of petiole.

Further qRT-PCR was carried out to study the levels of *NbUBC2* and *NbHUB1* in the silenced plants. *NbHUB1* and *NbUBC2* expression were decreased in the *NbHUB1*-silenced plants (S3A and S3B Fig). Interestingly, following ChiLCV infection, expression levels of both *NbHUB1* and *NbUBC2* transcripts were further decreased in the silenced plants (S3A and S3B Fig). A similar result was obtained from the *NbUBC2*-silenced plants. *NbHUB1* and *NbUBC2* expression levels were also decreased in *NbUBC2*-silenced plants which were further reduced in the ChiLCV-inoculated *NbUBC2*-silenced plants (S3A and S3B Fig).

Next, we studied the effects of silencing *NbUBC2* and *NbHUB1* on ChiLCV pathogenesis. For this purpose, pTRV:00-infiltrated, *NbUBC2*- and *NbHUB1*-silenced plants were inoculated with ChiLCV and the plants were regularly monitored for symptom severity (Fig 2E–2G and 2J). ChiLCV inoculated pTRV:00-infiltrated plants showed severe symptom (severity grade 4) (Fig 2E and 2J). Very mild symptoms were observed on *NbHUB1*-silenced plants (Fig 2F and 2J). ChiLCV failed to infect and induce symptoms on *NbUBC2*-silenced *N*. *benthamiana* plants (Fig 2G and 2J).

We studied ChiLCV DNA and transcript accumulation in *NbUBC2*-, and *NbHUB1*-silenced plants. For the detection of ChiLCV DNA, we initially performed a comparative Southern blotting assay. Results showed reduced viral titer in the *NbHUB1*-silenced plants (Fig 2K). The viral titre was further reduced in the *NbUBC2* silenced plants (Fig 2K). To measure the reduction in viral titre in the silenced plants, qPCR was carried out. The qPCR results showed significant reduction of viral DNA accumulation in *NbUBC2*-silenced (78%, p<0.001) and *NbHUB1*-silenced (60%, p <0.01), relative to non-silenced plants inoculated with ChiLCV (Fig 2L). *N*. *benthamiana* plants infiltrated with empty pTRV:00 did not yield amplification and were considered as negative controls for qPCR. Furthermore, qRT-PCR results, performed with the C2-specific primers, suggested reduced accumulation of C2 transcripts in either *NbUBC2*- or *NbHUB1*-silenced plants (Fig 2M). In comparison to pTRV:00-infiltrated plants, C2 transcripts level was decreased by 97% in *NbUBC2*-silenced (p <0.001) and 23% in *NbHUB1*-silenced (p<0.01) plants (Fig 2M). A similar result was observed when qRT-PCR was performed to check the transcript level of the virion-sense ORF, V2. In *NbUBC2*- and *NbHUB1*-silenced plants, V2 transcript accumulation was decreased [approximately 99% in *NbUBC2* (p <0.001) and 43% (p <0.01) in *NbHUB1* silenced plants] relative to that in wild-type inoculated plants (S3C Fig). A more drastic reduction of the viral transcripts was observed for the *NbUBC2*-silenced plants than the *NbHUB1*-silenced plants.

We analyzed the effect of TRV on ChiLCV multiplication. We isolated total DNA from either pTRV:00 or WT *N*. *benthamiana* plants inoculated with ChiLCV and carried out qPCR using ChiLCV specific primers. We did not observe significant difference of ChiLCV accumulation in either of these plants at 21 dpi (Fig 2N).

### Silencing of *NbHUB1* and *NbUBC2* affects post-translational modifications of global cellular histones in *N*. *benthamiana*

Furthermore, we investigated the global cellular level of histone modifications (H2B-ub and H3-K4me3) in pTRV:00 and silenced plants by carrying out immunoblotting with specific antibodies (Fig 2O). Total histone-enriched protein was isolated from the mock-infiltrated and the ChiLCV-inoculated wild-type and silenced (*NbUBC2 and NbHUB1*) plants after 21 dpi and immunoblotting was performed using specific antibodies. The results revealed reduced level of H2B-ub and H3-K4me3 modifications in the *NbHUB1-* and *NbUBC2*-silenced plants (Fig 2O). ChiLCV infection further decreased the H2B-ub and H3-K4me3 levels in the both silenced plants (Fig 2O). But the reduction was more prominent in the *NbUBC2-*silenced plants.

### ChiLCV promoter activity is decreased in *NbHUB1*-, and *NbUBC2*-silenced plants

**Fig 3:**
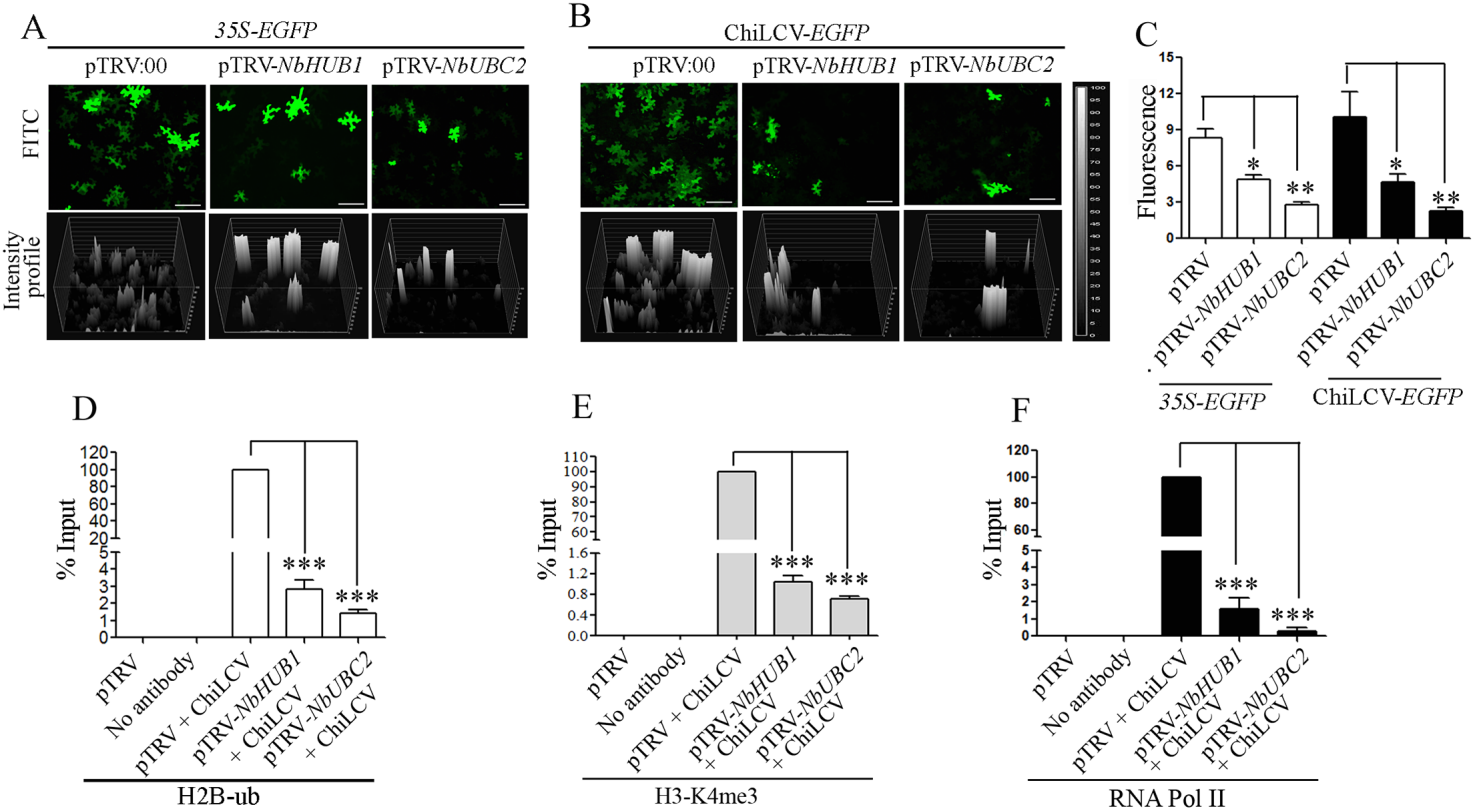
An analysis of comparative study of activity of ChiLCV and 35S promoter in silenced plants. (A) *35S-EGFP* constructs and (B) ChiLCV based EGFP expression vector (ChiLCV-*EGFP*). Scale bar = 100μm. Intensity profile of each samples was developed using NIS-Element 4.0 software. (C) Graphical representation of intensity of EGFP fluorescence of *35S-EGFP* in pTRV:00 (n = 244), pTRV-*NbHUB1* (n = 410) and pTRV-*NbUBC2* (n = 385). EGFP intensity of ChiLCV-*EGFP* in pTRV:00 (n = 225), pTRV-*NbHUB1* (360), pTRV-*NbUBC2* (n = 410). n = total number of cells observed. (D-F) Detection of H2B-ub, H3-K4me3 and DNA- dependant RNA Polymerase II occupancy on ChiLCV promoter in *NbUBC2*-, and *NbHUB1*-silenced plants. ChIP-PCR was carried out using anti-H2B-ub, anti-H3-K4me3, RNA Pol II antibodies and ChiLCV promoter specific primers. The asterisk denotes statistical significance differences between mean values determined by *t*-test (***p<0.001, **p<0.01, *p<0.05).

The activity of the ChiLCV promoter was assessed in plants using a ChiLCV-based expression vector where ORF V1 was replaced with EGFP in order to express the latter under virion sense promoter [52]. A tandem repeat of this construct was made in the pCAMBIA2300 vector and subsequently transferred into *A*. *tumefaciens* strain EHA105. After 21 dpi, pTRV-*NbHUB1*, pTRV-*NbUBC2* and pTRV:00 plants were again infiltrated with either a *35S*-*EGFP* construct or the ChiLCV-*EGFP* construct (Fig 3A and 3B). EGFP fluorescence was observed under a fluorescence microscope (Model 90i, Nikon, Tokyo, Japan) at 7 dpi and total number of cells (n) studied was recorded. An intensity graph of each samples showing EGFP fluorescence was generated using NIS-Element 4.0 software. EGFP expression was clearly observed to be directly proportional to the activity of the promoters. The intensity of EGFP in pTRV:00 was considered as 100% (Fig 3C, in *35S*-*EGFP* 8.33 ± 1.2 and in ChiLCV-*EGFP* 10.05 ± 3.6) and fluorescence of EGFP either in *NbHUB1*- or in *NbUBC2*-silenced plants was assessed accordingly. EGFP fluorescence of *35S-EGFP* was reduced to 58.33% (intensity 4.86 ± 0.67) in pTRV-*NbHUB1* (n = 410) and 33.24% (intensity 2.77 ± 0.45) in pTRV1-*NbUBC2* silenced plants (n = 385) (Fig 3C). The EGFP fluorescence indicating active transcription of the ChiLCV genome, was drastically reduced in both the *NbUBC2*- (22.18%; intensity 2.23 ± 0.63; n = 360) and *NbHUB1*-silenced (46.5%; intensity 4.67 ± 1.15; n = 410) plants (Fig 3C).

### *NbUBC2*-, and *NbHUB1*-silenced plants showed reduced histone post-translational modifications and RNA polymerase II occupancy on the viral promoter

Chromatin immunoprecipitation (ChIP) was performed to assess H2B-ub modification on the geminiviral promoter region using an anti-H2B-ub specific antibody and the viral promoter-specific PCR. Although sonicated samples indicated the presence of viral DNA in inoculated silenced plants, the H2B-ub level was significantly reduced on the viral promoter region in both the *NbUBC2*-, and *NbHUB1*-silenced plants (p <0.001, Fig 3D). Since, H2B-ub modifications correlated with H3-K4me3 modifications, ChIP experiments were further carried out using the anti-H3-K4me3 specific antibody. The results indicated a significantly reduced level of H3-K4me3 modification on the viral promoter region (p <0.001, Fig 3E). Monoubiquitination of H2B and trimethylation of H3-K4 is known to modify functions related to the activation of gene expression and to regulate elongation by RNA polymerase II [53]. Hence, RNA Pol II occupancy was further analyzed on the viral promoter region in the silenced plants using anti-RNA Pol II specific antibody. The RNA Pol II occupancy on the viral promoter region was also significantly reduced several folds in both types of silenced plants (p <0.001, Fig 3F).

### Subcellular localization of Rep, NbHUB1, and NbUBC2

**Fig 4:**
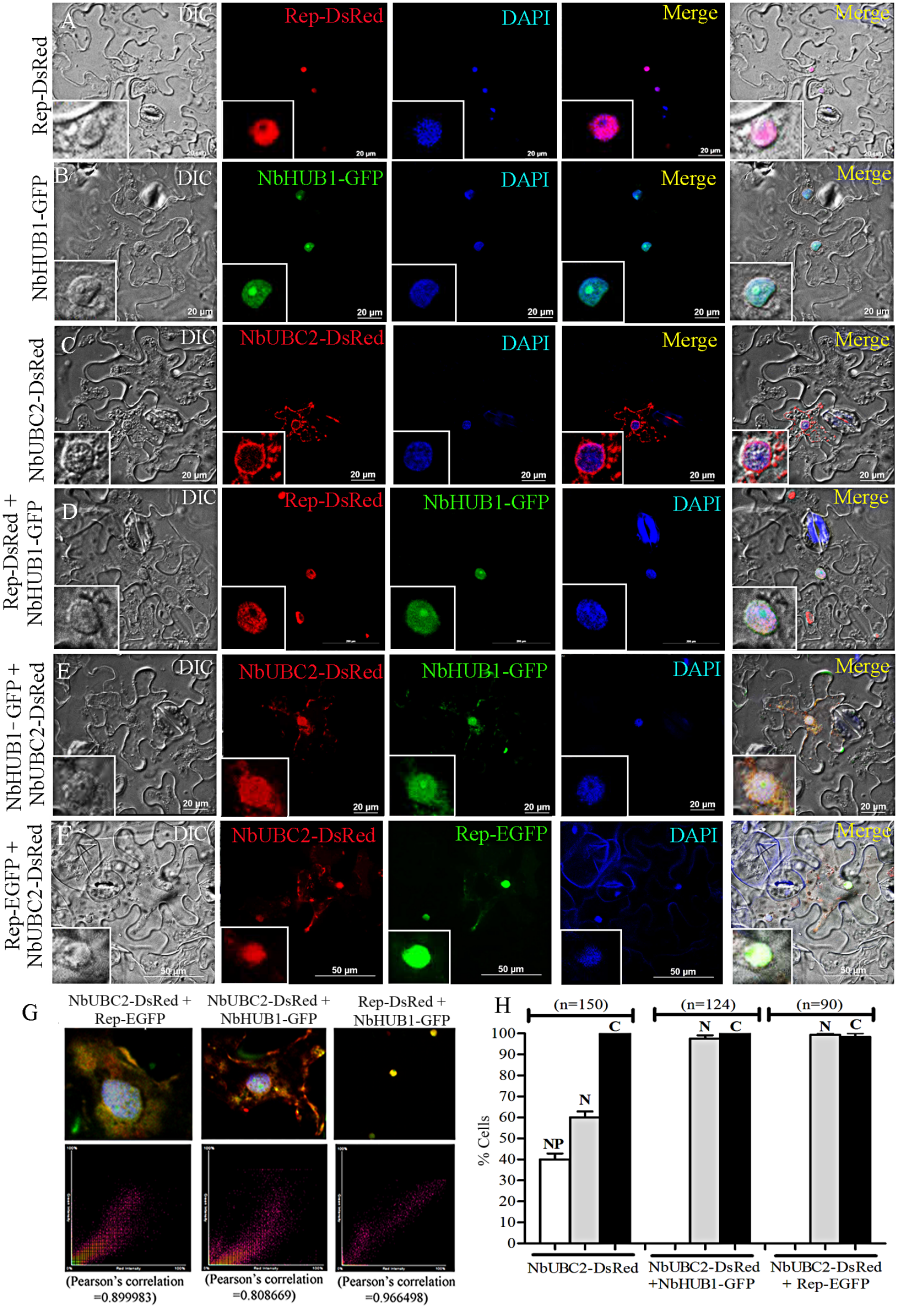
Sub-cellular localization and colocalization of Rep, NbHUB1, and NbUBC2 fluorescent fusion proteins in epidermal cells of *N*. *benthamiana* leaves. Sub- cellular localization of (A) Rep-DsRed, (B) NbHUB1-GFP (C) NbUBC2-DsRed, (D) Colocalization of Rep-DsRed and NbHUB1-GFP, (E) Colocalization of NbUBC2-DsRed and NbHUB1-GFP, (F) Colocalization of Rep-EGFPand NbUBC2-DsRed. DAPI is used as nuclear marker. Scale bar = 20μm (A-C, E), 200μm (D), and 50 μm (F). (G) Pearson’s correlation for colocalization was calculated using the NIS-Element 4.0 software. Pearson's correlation coefficient of NbUBC2-DsRed and Rep-EGFP (0.89), NbUBC2-DsRed and NbHUB1-GFP (0.80), Rep-DsRed and NbHUB1-GFP (0.96) are mentioned. (H) Graphical representation of percentage of cells showing NbUBC2-DsRed localization alone (n = 150) and in the presence of either NbHUB1-GFP (n = 124) or Rep-EGFP (n = 90). ‘n’ denotes total number of cells observed.

The subcellular localization of NbHUB1, NbUBC2 and Rep proteins was investigated using enhanced green fluorescent protein (EGFP) and Discosoma red fluorescent protein (DsRed) as the reporter in transient expression assays in the epidermal cells of *N*. *benthamiana* leaves. NbUBC2, and Rep were fused with DsRed separately whereas NbHUB1 was expressed as fusion of GFP. The in-frame fusion proteins were expressed from the CaMV *35S* promoter. Fig 4 shows the confocal microscopy images of fluorescence resulting from agroinfiltration of the fusion constructs into the leaves of the wild-type *N*. *benthamiana*. Fluorescence of EGFP and DsRed from the vector infiltrated plants was observed throughout the cell and served as controls (S4A Fig).

We analyzed the presence of the nuclear localization sequence (NLS) in Rep, NbHUB1 and NbUBC2 proteins on a cNLS mapper (http://nls-mapper.iab.keio.ac.jp/cgi-bin/NLS_Mapper_help.cgi). The sequence analysis predicted a nuclear localization signal (NLS) in the ChiLCV Rep protein (between amino acid residues 244 and 273) (S3 Text). In the transient expression assay, a Rep-DsRed signal was exclusively observed in the nuclei of the infiltrated cells as confirmed by DAPI staining (Fig 4A). Rep-DsRed signal was observed throughout the nucleoplasm except in the nucleolus (Fig 4A, S4B Fig). The position of nucleolus in the nucleus was detected by co-expressing Fib-mRFP with Rep-EGFP (S4B Fig).

The NbHUB1-GFP fusion protein was expressed in *N*. *benthamiana* through the pCXDG vector and the results indicated nuclear localization of NbHUB1-GFP fluorescence. Amino acid sequence analysis of NbHUB1 revealed the presence of two sequences similar to NLSs, located between residues 288 to 320, and 703 to734 (S3 Text). The NbHUB1-GFP fluorescence was observed to overlap with the DAPI stain in the nucleus (Fig 4B). In most of the cells, NbHUB1-GFP fluorescence was also noticed at a distinct body in the nucleoplasm (Fig 4B). In order to identify the specific location of NbHUB1 in the nucleoplasm, nucleolus specific maker, Fibrillarin-mRFP (Fib-mRFP) was used for co-localization study. Results indicated merging of both NbHUB1 and Fibrillarin in the nucleolus (S4B Fig). The protein sequence analysis failed to indicate the presence of NLS in NbUBC2 (S3 Text). The NbUBC2-DsRed fusion protein, in contrast, was localized predominantly in the cytoplasm rather than the nucleus (Fig 4C). To confirm the cytoplasmic localization of NbUBC2, we stained the epidermal cells of *N*. *benthamiana* leaves expressing NbUBC2-EGFP with ER tracker. The signal of ER tracker merged with the fluorescence of NbUBC2-EGFP in the cytoplasm (S4B Fig). In addition to cytoplasmic localization of NbUBC2-DsRed, 60% of the cells showed fluorescence throughout the nucleus while 40% of the cells (n = 150) exhibited higher NbUBC2-DsRed signal at the nuclear periphery than in the nucleoplasm (Fig 4H).

### Like cognate partner NbHUB1, geminivirus Rep protein redirects NbUBC2 protein into the nucleus

Since Rep and NbHUB1 are exclusively localized in the nucleus and NbUBC2 is also partially localized in the nucleus, in order to understand the localization of viral Rep protein in presence of NbHUB1 or NbUBC2, we performed a co-localization study in epidermal cells *of N*. *benthamiana* leaves. The colocalization study was carried out by infiltrating *N*. *benthamiana* with *Agrobacterium* cells containing equal amount of NbHUB1-GFP and Rep-DsRed constructs. Both of the proteins were observed to colocalize in nuclei of all the cells under study (n = 128) as they overlap with the DAPI stain (Fig 4D).

Furthermore, colocalization of Rep-DsRed and NbHUB1-GFP was also validated by calculating Pearson’s correlation coefficient using NIS-Element 4.0 software. A correlation coefficient between 0.1 and 1.0 is indicative of colocalization. Pearson’s correlation coefficient of Rep-DsRed and NbHUB1-GFP was found to be 0.97, suggesting colocalization of these two proteins (Fig 4G). Moreover NbHUB1-GFP co-infiltrated with its cognate NbUBC2-DsRed, which indicated the presence of NbUBC2 in both the cytoplasm and the nucleus (Fig 4E). In addition, the high value Pearson’s correlation coefficient (0.80) of NbUBC2-DsRed and NbHUB1-GFP (Fig 4G) indicated that these proteins are also colocalized in all the cells showing signals (100%; n = 124). The colocalization study of NbUBC2-DsRed and Rep-EGFP showed that unlike NbHUB1, NbUBC2-DsRed and Rep-EGFP are colocalized predominantly in the nucleus than in the cytoplasm (Fig 4F). Interestingly, unlike cells infiltrated with NbUBC2-DsRed alone, colocalization of Rep-EGFP resulted in cent-per cent (n = 90) mobilization of NbUBC2-DsRed into the nucleus of the cells analyzed (Fig 4H). Pearson’s correlation coefficient for NbUBC2 and Rep-EGFP was calculated to be 0.89, which is a convincing score for colocalization (Fig 4G).

### Rep localization is not altered either in *NbHUB1*- or *NbUBC2*-silenced plants

**Fig 5:**
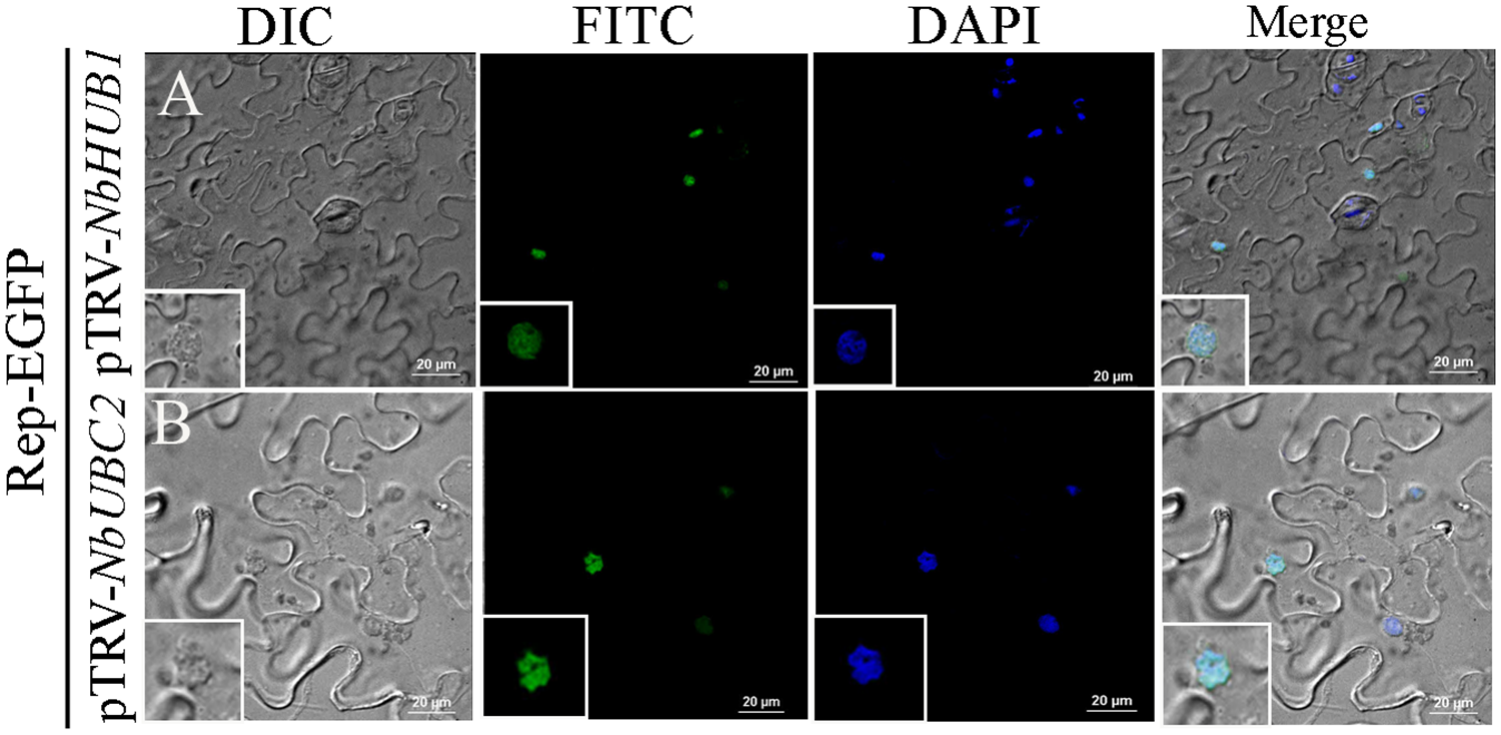
Sub-cellular localization of Rep-EGFP in (A) pTRV-*NbHUB1* and (B) pTRV-*NbUBC2* silenced plants. DAPI is used as nuclear marker. Scale bar = 20μm.

In order to check the individual roles of NbHUB1 and NbUBC2 in the subcellular localization of Rep, we expressed Rep-EGFP in *N*. *benthamiana* lower epidermis in which either *NbHUB1* or *NbUBC2* was transiently silenced. In each case, approximately 140 cells were observed from 5 different leaves, Rep-EGFP was observed to be localized in the nucleus regardless of either *NbHUB1* or *NbUBC2* silencing (Fig 5).

### ChiLCV induces formation of punctate bodies consisting of Rep in the nucleus

To test whether viral infection can alter the subcellular localization of host-encoded and virally encoded proteins in the plant cell, we performed localization studies of NbHUB1, NbUBC2 and the Rep proteins in the presence of ChiLCV. The coinfiltration of the NbHUB1-GFP construct with ChiLCV into *N*. *benthamiana* indicated confinement of the NbHUB1-GFP fluorescence signal in the nucleus of the infiltrated cells (S5A Fig). The experiment with NbUBC2 was particularly interesting: unlike the results for the case of cells infiltrated only with NbUBC2-DsRed described above, the NbUBC2-DsRed fluorescence signal was prominent in the cytoplasm and in the nucleus in presence of ChiLCV infection (S5B Fig).

We co-infiltrated *N*. *benthamiana* plants with Rep-DsRed and ChiLCV. Interestingly Rep-DsRed signal, in the presence of ChiLCV, appeared as several irregularly shaped punctate bodies located randomly in the nuclei (S5C Fig). Coinfiltration of NbUBC2-DsRed and Rep-EGFP with ChiLCV also indicated presence of irregularly shaped punctate bodies in the nucleus (S5D Fig).

**Fig 6:**
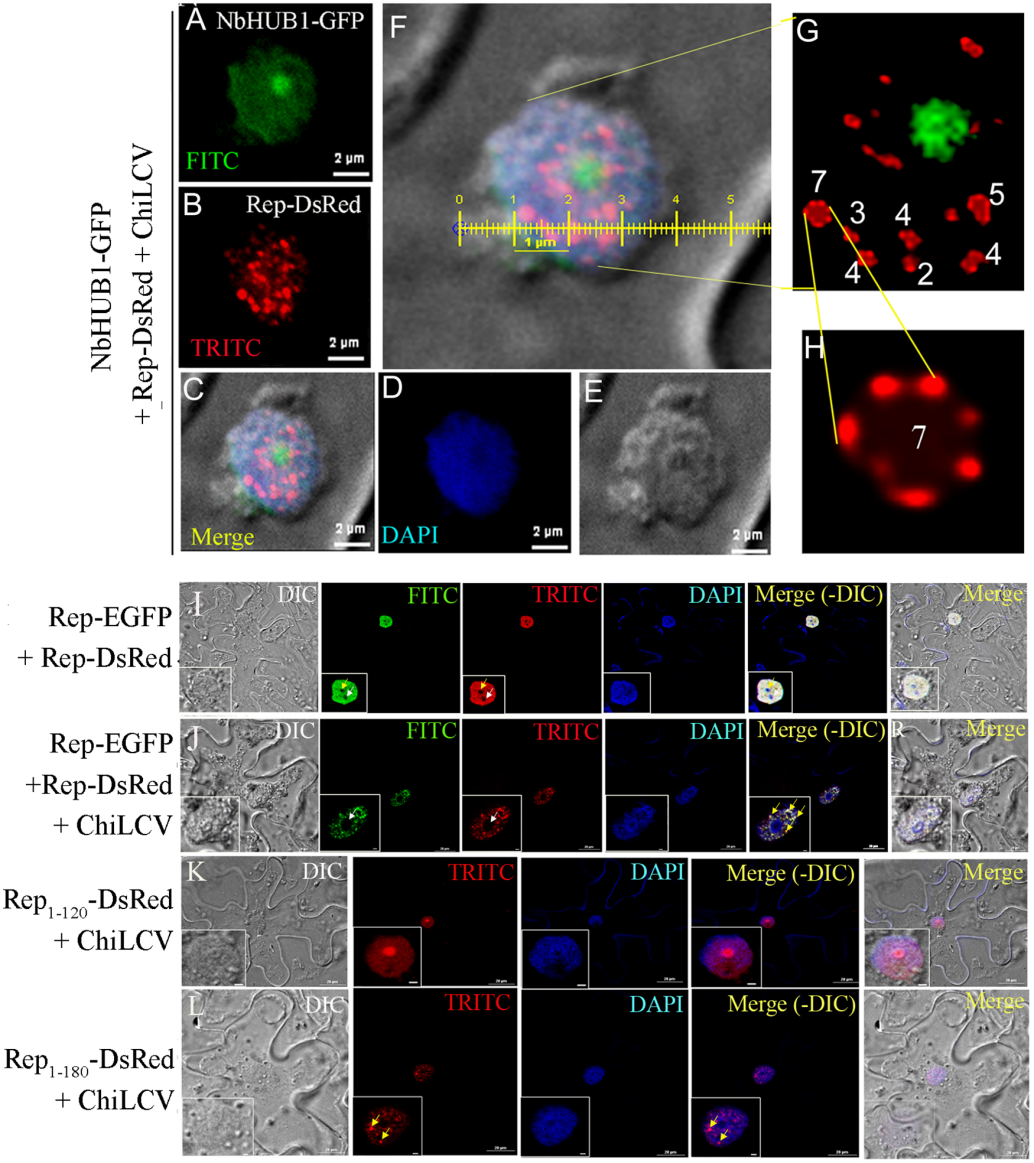
ChiLCV induces formation of punctate bodies consisting of Rep in the nucleus. (A-F) Colocalization of Rep-DsRed and NbHUB1-GFP in presence of ChiLCV at 7 dpi. Scale bar = 2μm. (G) Magnified view of the nucleus. Closer examination of punctate bodies indicated “beads-on-string” like organization and the number of “beads” varied from two to seven. (H) Enlarged picture of one of the representative punctate bodies consisting of seven beads organized in a circular manner. The punctate bodies were organized as “beads-on-string”-like structures that measured as approximately 400 nm in diameter and the average size of the beads was calculated to be ~100 nm. The brightness and contrast of the image were adjusted by using Adobe Photoshop (www.adobe.com/in/products/photoshop) in order to improve image clarity. (I) Rep-EGFP with Rep-DsRed, (J) Rep-EGFP and Rep-DsRed organized as punctate structures in presence of ChiLCV, (K) Rep_1-120_-DsRed failed to form punctate bodies in presence of ChiLCV and (L) Rep_1-180_-DsRed derived punctate bodies in presence of ChiLCV.

Surprisingly, when *N*. *benthamiana* plants coinfiltrated with the constructs expressing NbHUB1-GFP, Rep-DsRed and ChiLCV, the NbHUB1-GFP fluorescence was confined to nuclei as in the case of NbHUB1-GFP alone, but Rep-DsRed in this case formed several orderly punctate bodies of different shapes in the nuclei (Fig 6A–6H and S5E Fig). However, upon close examination, these punctate bodies revealed “beads-on-string” like appearance. We adjusted the contrast of the image using Adobe Photoshop (version 8) in order to bring clarity to the magnified view of a representative punctate body formed by Rep (Fig 6G and 6H). These punctate bodies were different from those observed in case of Rep-DsRed along with ChiLCV, which did not show any definite organization and shape (S5F–S5H Fig). The number of bead-like structures varied from two to seven in punctate bodies (Fig 6G). Interestingly, in one such punctate body Rep-DsRed appeared to be organized as a circular “string” of seven “beads” (Fig 6H) which measured approximately 400 nm in diameter in total while a single bead was estimated approximately 100 nm in diameter (Fig 6F).

We also checked the functional status of the fusion proteins (NbHUB1-EGFP, NbUBC2-DsRED and Rep-EGFP). RepEGFP_6xHis_ and EGFP_6xHis_ tagged fusion proteins were overexpressed in *E*. *coli* strain BL21(DE3) and purified to test ATP hydrolysis activity (S6A and S6B Fig). RepEGFP_6xHis_ fusion protein hydrolyzed [γ ^32^P]ATP similar to Rep_6xHis_ but EGFP_6xHis_ failed to exhibit ATPase activity (S6C Fig). This experiment showed that EGFP_6xHis_ does not influence the function of the Rep protein. Furthermore, we overexpressed EGFP, NbHUB1-GFP and NbUBC2-EGFP in *N*. *benthamiana* plants infected with ChiLCV (at 21 dpi). To check the functional role of NbHUB1-GFP and NbUBC2-EGFP, immunoblotting assays were performed with anti-H2B antibody. The results showed enhanced H2B-ub in *N*. *benthamiana* plants infiltrated with either NbHUB1-GFP or NbUBC2-EGFP in comparison with plants infiltrated with EGFP (S6D Fig).

In order to rule out the possibility of punctate bodies being an artefact of DsRed, we co-expressed Rep-EGFP and Rep-DsRed either in presence or absence of ChiLCV (Fig 6I and 6J). The punctate bodies were clearly visible when Rep-EGFP was expressed along with ChiLCV and merged with the punctate bodies of Rep-DsRed (Fig 6J). These results also suggest involvement of more than one Rep molecules in the formation of punctate bodies. It is important to note that the punctate bodies like structures were not visible when Rep-EGFP and Rep-DsRed were co-expressed in absence of ChiLCV (Fig 6I).

Furthermore, we also determined the minimal region of Rep protein required for formation of the punctate bodies. We coexpressed Rep_1-120_-DsRed and Rep_1-180_-DsRed with ChiLCV in the epidermal cells of *N*. *benthamiana* plants. Rep_1-120_-DsRed failed to form punctate bodies in presence of ChiLCV (Fig 6K), whereas Rep_1-180_-DsRed organized as punctate bodies in presence of ChiLCV as observed in the case of full length Rep protein and ChiLCV (Fig 6L). The above observation suggests that the oligomerisation domain of Rep protein (121–180 aa) is indispensable for the punctate body formation.

### ChiLCV Rep protein interacts with NbHUB1 and NbUBC2

**Fig 7:**
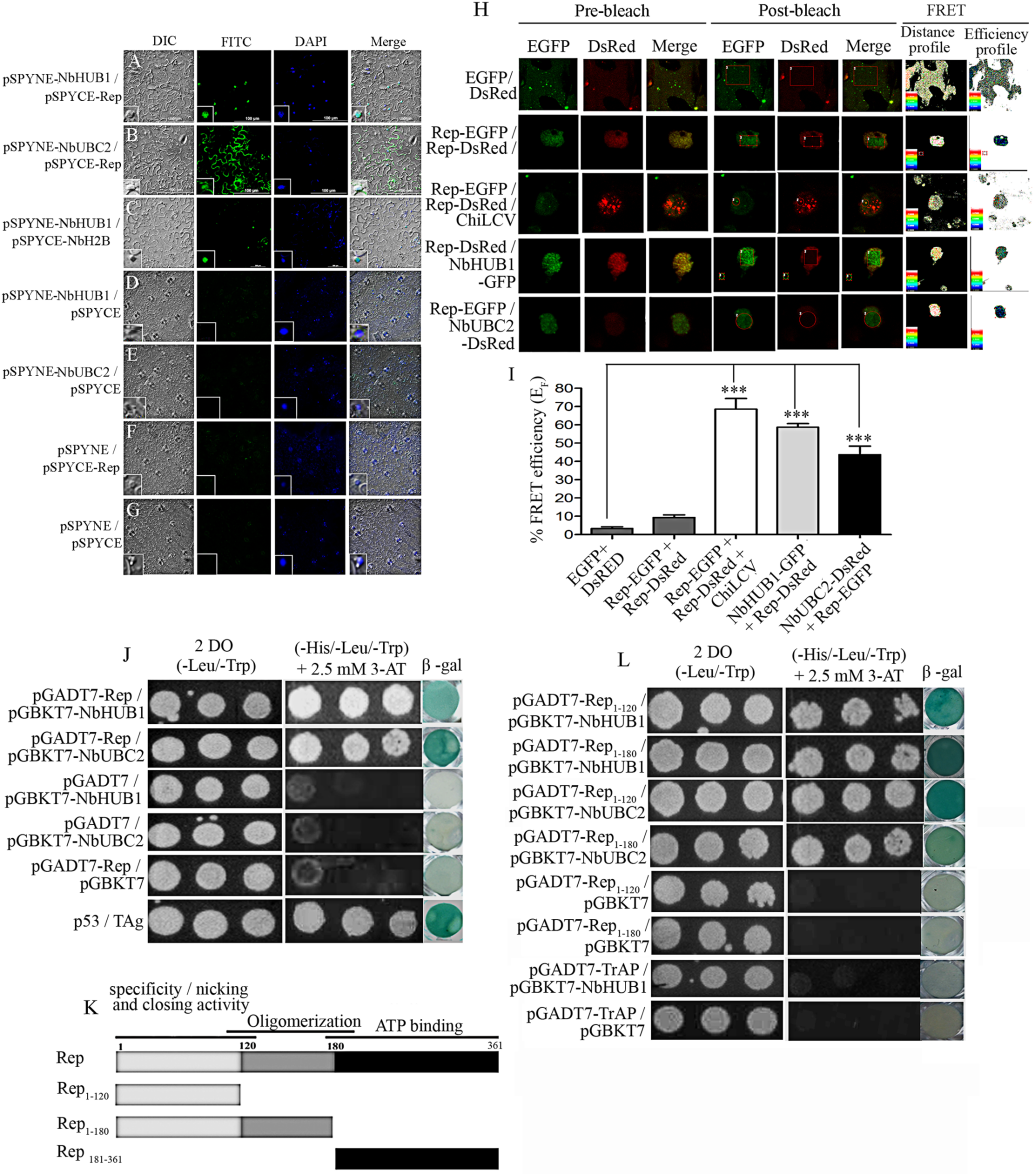
Rep interacts with NbHUB1 and NbUBC2 in vivo. In planta bimolecular fluorescence complementation assays were performed in the lower epidermis of *N*. *benthamiana* leaves (at 5 dpi). NbHUB1 and NbUBC2 were expressed as in-frame fusion with the N-terminal of the YFP protein using the pSPYNE vector. The Rep protein was expressed with C-terminal region of the YFP protein using pSPYCE vector. BiFC assay of interaction of (A) pSPYNE-NbHUB1 and pSPYCE-Rep, (B) pSPYNE-NbUBC2 and pSPYCE-Rep, (C) pSPYNE-NbHUB1 and pSPYCE-NbH2B. (D) pSPYNE-NbHUB1 / pSPYCE, (E) pSPYNE-NbUBC2 / pSPYCE, (F) pSPYCE-Rep / pSPYNE and (G) pSPYNE / pSPYCE serve as control. Scale bar = 100μm. (H) The protein–protein interaction was also monitored by FRET microscopy in the epidermal cells of *N*. *benthamiana* leaves coexpressing EGFP and DsRed, Rep-EGFP and Rep-DsRed, NbHUB1-GFP and Rep-DsRed, Rep-EGFP and NbUBC2-DsRed. Representative acceptor photobleaching images show EGFP (donor) and DsRed (acceptor) channels before and after bleaching. After bleaching, DsRed fluorescence decreases in the bleached are as indicated by rectangles/circles. The FRET profiles showed the proximity between the donor and acceptor molecules and the efficiency of FRET. (I) Graph represents the quantification of FRET efficiency (E_F_) of three independent experiments. (J) Yeast two-hybrid assay of Rep and NbHUB1, Rep and NbUBC2 on non selective media (-Leu / -Trp) and selective media (-His / -Leu / -Trp with 2.5 mM 3-AT). β galactosidase activity were checked for each combination and corresponding negative controls._P_53 and TAg served as positive control. (K) Schematic diagram of deletion mutants of Rep protein used in yeast two-hybrid assays. (L) Yeast two-hybrid and β-galactosidase assays of in vivo interaction between deletion mutants of Rep protein and TrAP with NbHUB1 and NbUBC2.

In planta interaction study of ChiLCV Rep protein with NbHUB1 and NbUBC2 was performed by carrying out bimolecular fluorescence complementation (BiFC) assays. The Rep protein was expressed as a fusion protein with the C-terminal fragment of the yellow fluorescent protein (YFP) (pSPYCE-Rep) and either NbHUB1 or NbUBC2 was fused with the N-terminal fragment of the YFP protein (pSPYNE-NbHUB1 or pSPYNE-NbUBC2). Agroinfiltration was carried out with pSPYCE-Rep and pSPYNE-NbHUB1. Here, the GFP, and DAPI fluorescence were observed to overlap revealing that Rep and NbHUB1 interacted with each other in the nucleus (Fig 7A). Furthermore, an interaction between Rep and NbUBC2 was observed in the cytoplasm as well as in the nucleus (Fig 7B). The interaction of NbHUB1 and NbH2B in the nucleus served as a control (Fig 7C). We also performed experiments with corresponding empty vectors and constructs (pSPYNE-NbHUB1with pSPYCE, pSPYNE-NbUBC2 with pSPYCE, pSPYCE-REP with pSYPNE, and pSPYNE with pSPYCE) in order to exclude any bias in the experiments, and no such interaction signal was obtained in these cases (Fig 7D–7G).

Furthermore, interaction of the viral Rep protein with Rep protein itself, NbHUB1 and NbUBC2 was demonstrated directly in planta by fluorescence resonance energy transfer (FRET) microscopy (Fig 7H and 7I). FRET between a pair of proteins provides powerful means to monitor protein-protein proximity and interaction in living cells. FRET efficiency between these proteins was determined by acceptor photobleaching and calculation of FRET efficiency (E_F_). DsRed and EGFP were transiently coexpressed in the *N*. *benthamiana* epidermal cells and FRET was performed following [54,55]. We transiently expressed our proteins of interest as fusion proteins with either DsRed or EGFP in the epidermal cells of *N*. *benthamiana*. In the FRET experiment between EGFP and DsRed approximately 6% of FRET efficiency was noticed which was far below the threshold value [55] of being considered as a positive interaction (Fig 7H and 7I). FRET between EGFP and DsRed were considered as negative control for other experiments. Rep-EGFP and Rep-DsRed combination showed very low level of FRET efficiency (9.33 ± 2.55%) indicating no interaction (Fig 7H and 7I). Interestingly, FRET efficiency of Rep-EGFP and Rep-DsRed increased to 68.66 ± 10.2% in the presence of ChiLCV when Rep proteins were organized as punctate bodies (Fig 7H and 7I). We observed FRET efficiency of 58.66 ± 3.5% and 43.66 ± 8.08% in the case of NbHUB1-GFP with Rep-DsRed and Rep-EGFP with NbUBC2-DsRed, respectively confirming interaction between these pairs of proteins (Fig 7H and 7I).

The in planta interactions of Rep protein with NbHUB1 and NbUBC2 proteins were further validated by yeast two-hybrid assays. Nucleotide sequences representing Rep protein was cloned in pGADT7 vector whereas *NbHUB1* and *NbUBC2* were cloned separately in the yeast expression vector pGBKT7. To map the region of Rep protein that interacts with NbHUB1 and NbUBC2 proteins, we generated deletion mutants of Rep protein. DNA sequences corresponding to Rep_1-120_ (i.e. amino acid residues 1 to 120 of Rep), Rep_1-180_ and Rep_181-361_ were cloned in the pGADT7 expression vector to form pGADT7- Rep_1-120_, pGADT7- Rep_1-180_ and pGADT7- Rep_181-361,_ respectively (Fig 7K).

*Saccharomyces cerevisiae* strain AH109 was cotransfected with either the pGADT7-Rep and pGBKT7-NbHUB1 constructs or the pGADT7-Rep and pGBKT7-NbUBC2 constructs, and then β-galactosidase assays were carried out. Rep protein interacted with NbHUB1 and NbUBC2 in the yeast two-hybrid assays as confirmed by the results of the β-galactosidase activity (Fig 7J). pGADT7-Rep with pGBKT7, pGBKT7-NbUBC2 with pGADT7, and pGBKT7-NbHUB1 with pGADT7 constructs did not show β-galactosidase activity, and therefore served as negative controls for the experiments (Fig 7J). The interaction between p53 and large T antigen (TAg) showed β galactosidase activity and was used as a positive control (Fig 7J).

We cotransfected yeast cells with the constructs of either NbHUB1 or NbUBC2 with each Rep deletion mutants and performed β galactosidase assays (Fig 7L). NbHUB1 and NbUBC2 showed interaction with the Rep_1-120_ and Rep_1-180_ domains. Notably, NbHUB1 showed interaction with Rep_181-361_ but NbUBC2 failed to interact with Rep_181-361_(S7A Fig). These interactions were also confirmed by β-galactosidase assays (S7A Fig). Constructs such as Rep_1-120_+pGBKT7, Rep_1-180_ +pGBKT7 and Rep_181-361_+pGBKT7 did not exhibit β-galactosidase activity and therefore, served as negative controls (Fig 7L and S7A Fig). Since previous reports suggested that ORF C2 codes for a transcription activator protein (TrAP), we were interested to know whether C2 can also play a role in the regulation of H2B-ub modification by interacting with NbHUB1. Yeast two-hybrid assays involving pGADT7-TrAP with pGBKT7-NbHUB1 failed to show an interaction where pGADT7-TrAP with pGBKT7 served as the negative control (Fig 7L). Furthermore, we made an effort to map the region of the NbHUB1 protein that interacts with viral Rep protein. We generated, and cloned deletion mutants of NbHUB1 into the pGBKT7 vector to form pGBKT7-NbHUB1_1-213_ and pGBKT7-NbHUB1_1-593_ (S7B Fig). We performed yeast two-hybrid and β-galactosidase assays (S7C Fig). The combinations pGBKT7-NbHUB1_1-213_ with pGADT7-Rep and pGBKT7-NbHUB1_1-213_ with pGADT7 showed weak but same intensity of β-galactosidase activity (S7C Fig), whereas pGBKT7-NbHUB1_1-593_ with pGADT7-Rep exhibited an interaction in β-galactosidase assay (S7C Fig). However, NbHUB1_1-593_ with pGADT7 failed to show interaction.

Further, we carried out FRET experiments to monitor live interaction between NbHUB1 and NbUBC2 with the Rep mutants. NbHUB1-GFP showed significant interactions with all the Rep mutants; Rep_1-120_-DsRed (45±5%), Rep_1-180_-DsRed (44±4%) and Rep_181-361_-DsRed (58.50±10%) (S8A and S8B Fig). NbUBC2-EGFP showed considerable interaction with Rep_1-120_-DsRed (38±4%) and Rep_1-180_-DsRed (40.5±6%). The combination of NbUBC2-EGFP and Rep_181-361_-DsRed showed FRET efficiency similar to negative control of Rep mutants that measured less than 20% of efficiency (S8A and S8B Fig).

### Subcellular localization of Rep-_1-120_ and Rep _1–180_ mutants

**Fig 8:**
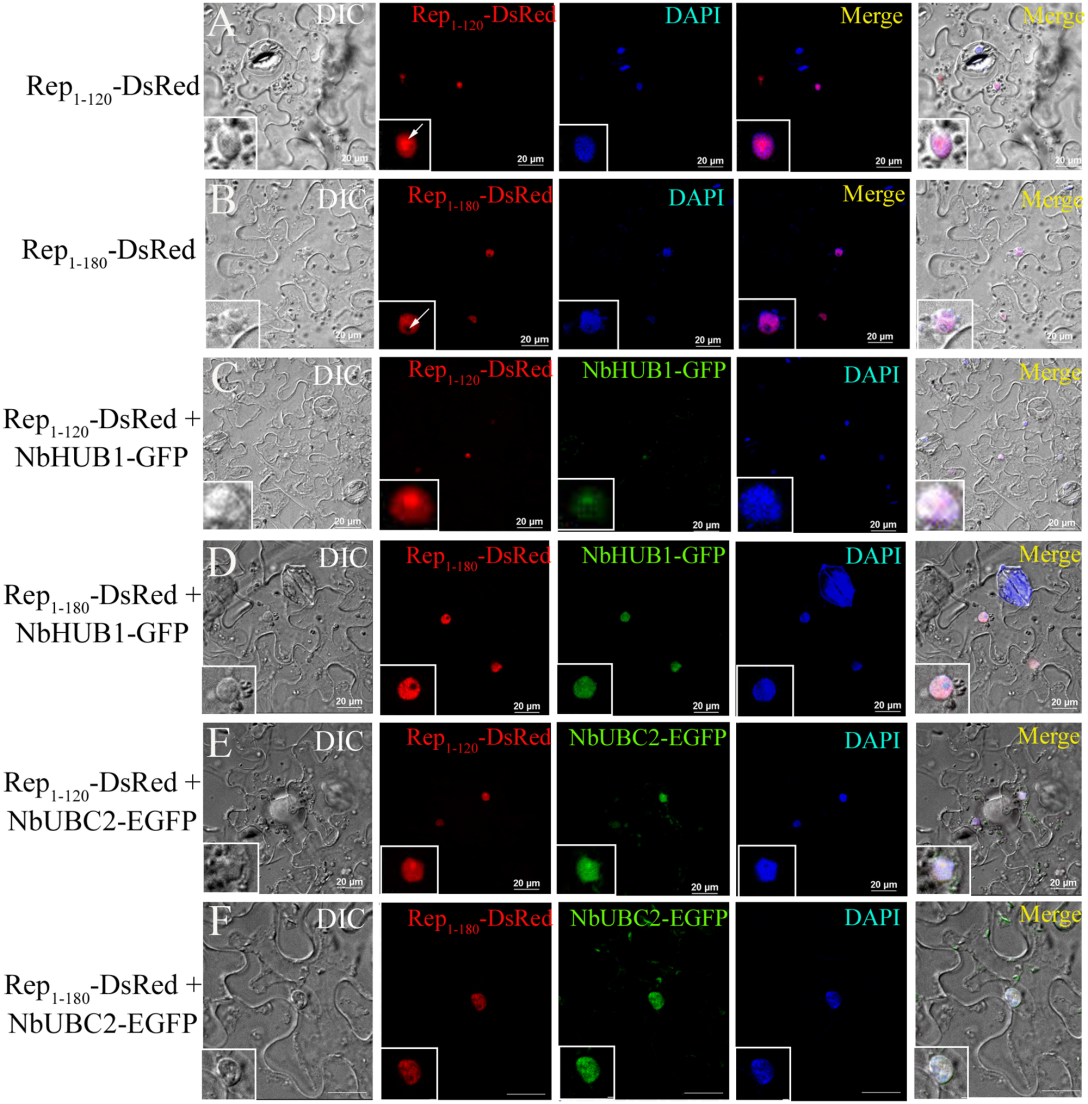
Subcellular localization and colocalization of deletion mutants of Rep. (A) Rep_1-120_-DsRed was expressed in epidermal cells of *N*. *benthamiana* leaves under CaMV *35S* promoter. At 3 dpi, all observed cells (n = 15) showed fluorescence of Rep_1-120_-DsRed in the nucleus and in the nucleolus (arrow). (B) Rep_1-180_-DsRed fluorescence was restricted throughout the nucleus except the nucleolus (arrow). (C) Colocalization of NbHUB1-GFP and Rep_1-120_-DsRed was observed. (D) Rep_1-180_-DsRed was colocalized in the nucleus with NbHUB1-GFP. (E) Rep_1-120_-DsRed colocalized with NbUBC2-EGFP in nucleus. (F) Rep_1-180_-DsRed also colocalized with NbUBC2-EGFP in nucleus. Scale bars = 20 μm.

In the nucleus, NbHUB1 (as E3 ligase) plays crucial role to specify the substrate for ubiquitination. Further, our results showed colocalization and interaction of Rep protein with NbHUB1. Rep protein consists of different regions that are known to perform specific functions during the viral multiplication and pathogenesis [14,15,48]. To investigate the subcellular localization of Rep mutants, Rep_1-120_-DsRed and Rep_1-180_-DsRed fusion constructs were developed and subsequently expressed in the epidermal cells of *N*. *benthamiana* leaves. In this experiment each cell (n = 15) showed Rep_1-120_-DsRed signal throughout the nucleoplasm including the nucleolus (arrow; Fig 8A). All the cells expressing Rep_1-180_-DsRed (100%; n = 16) exhibited fluorescence in the nuclei except in the nucleolus (arrow, Fig 8B). Furthermore, Rep_1-120_-DsRed and NbHUB1-GFP fluorescence were colocalized both in the nucleus and nucleolus (Fig 8C). In contrast, Rep_1-180_-DsRed and NbHUB1-GFP localization was restricted to nucleoplasm (Fig 8D) while NbHUB1-GFP fluorescence was maintained in the nucleolus also. Both Rep_1-120_-DsRed and Rep_1-180_-DsRed were colocalized with NbUBC2 in the nucleus (Fig 8E and 8F).

### The host monoubiquitnation machinery does not ubiquitinate Rep protein

**Fig 9:**
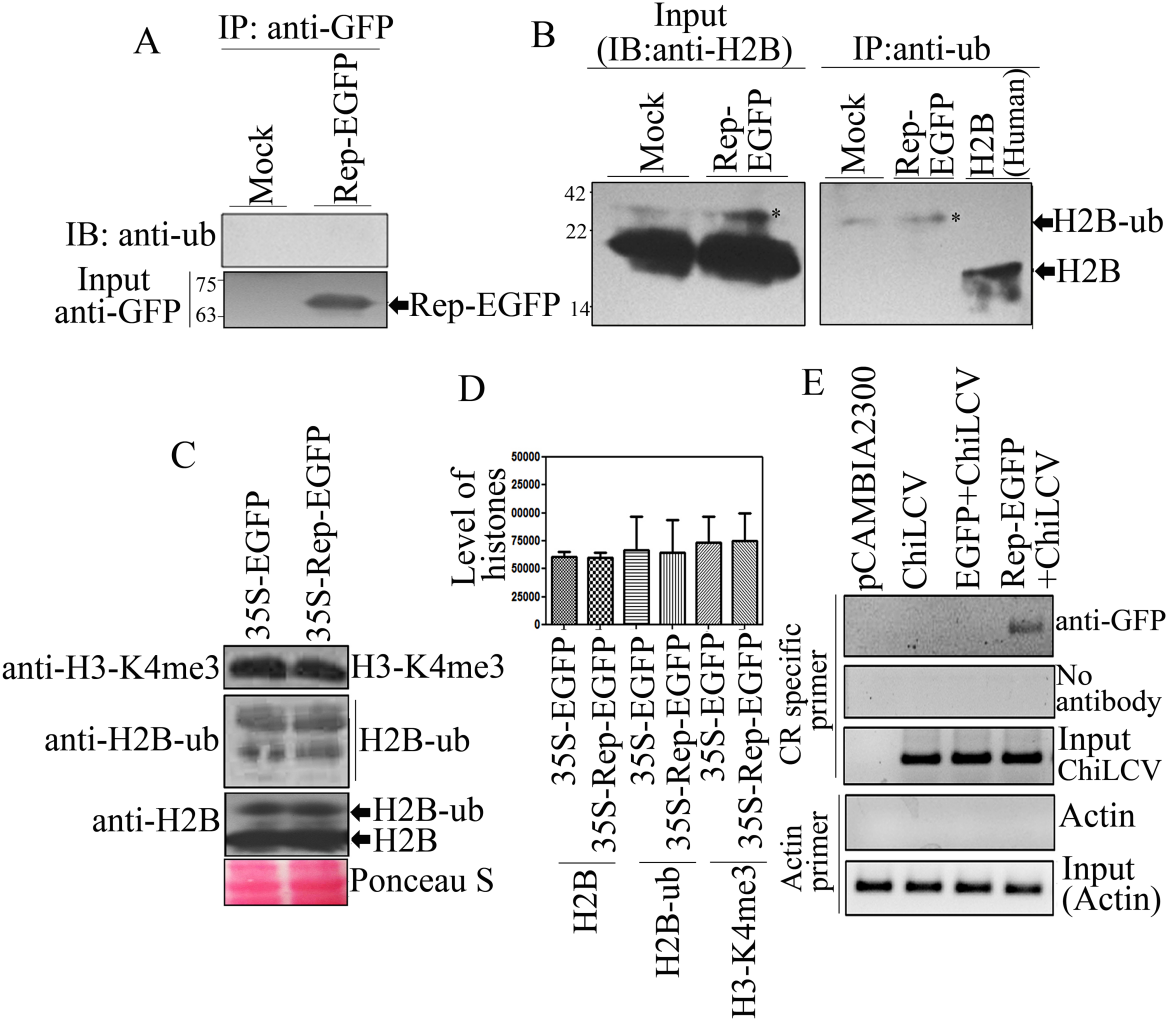
Rep protein neither gets ubiquitinated nor influences global histone modifications. (A) Detection of Rep-EGFP ubiquitinationin vivo. Immunoprecipitation was carried out from the total nuclear proteins using anti-GFP specific antibody at 7 dpi followed by immunoblotting with an anti-ubiquitin specific-antibody. Immunoblotting with anti-GFP specific antibody serves as input. (B) In vivo ubiquitination assay of H2B. Immunoprecipitation of ubiquitinated H2B using anti-ubiquitin specific antibody and immunoblotting was carried out with anti-H2B specific antibody. Immunoblotting of total nuclear proteins with H2B served as input control whereas human H2B served as control of unmodified H2B. (C) The effect of Rep protein on the total cellular histone modifications. Immunoblotting was performed using anti-H2B, anti-H2B-ub, and anti-H3-K4me3 specific antibodies from the total histone-enriched nuclear proteins isolated from EGFP-, and Rep-EGFP-infiltrated leaves. (D) Quantitative graphical representation of total cellular histone modifications. (E) Rep protein binds to ChiLCV DNA unlike host DNA. ChIP-PCR assays were carried out using the anti-GFP specific antibody and viral promoter specific primers. pCAMBIA2300, Rep-EGFP, EGFP with ChiLCV, and Rep-EGFP with ChiLCV-infiltrated leaves were subjected to ChIP-PCR analysis. The sample processed without antibody was considered as a control. Actin is considered as negative control.

Since the previous experiments confirmed the interactions between ChiLCV Rep protein and either NbHUB1 or NbUBC2, it is important to study whether Rep is also ubiquitinated in the plant. Rep-EGFP constructs were infiltrated into *N*. *benthamiana* leaves and immunoprecipitation was performed using anti-GFP specific antibody followed by immunoblotting with an anti-ubiquitin specific antibody. The immunoprecipitated samples included an approximately 66 kDa protein, consistent with the expression and presence of the Rep-EGFP protein (Fig 9A). Furthermore, immunoblotting with anti-ubiquitin did not show a signal in the Rep-EGFP samples, suggesting that Rep was not ubiquitinated in *N*. *benthamiana* (Fig 9A).

To validate the result of in vivo monoubiquitination assay, we performed immunoprecipitation of H2B-ub using ubiquitin specific antibody and subsequently immunoblotting was carried out with H2B specific antibody. Our results showed ubiquitination of H2B from the mock-, and Rep-EGFP infiltrated leaves (Fig 9B). The signal for unmodified H2B of *N*. *benthamiana* was not detected in immunoprecipitation samples by immunoblotting with anti-H2B specific antibody (Fig 9B). We also used Human H2B-unmodified (New England Biolabs, Massachusetts, United Kingdom) as control. In input both forms of H2B (unmodified, and ubiquitinated) were detected (Fig 9B).

### Rep does not affect global cellular level of modifications of histones

In one of the previous experiments, ChiLCV infection altered the global level of H2B-ub in the infected plants. We, therefore, tested the ability of Rep protein to affect the global cellular level of post-translational modifications of histones. Total histone protein was isolated from EGFP-, and Rep-EGFP-infiltrated *N*. *benthamiana* plants and immunoblotting was performed separately with anti-H2B, anti-H2B-ub and anti-H3-K4me3 specific antibodies. We did not observe any significant difference in H2B, H2B-ub and H3-K4me3 modifications between EGFP-and Rep-EGFP infiltrated leaves (Fig 9C and 9D).

### Rep specifically binds to viral DNA rather than plant DNA

To investigate the specificity of the binding of Rep to the viral DNA, we carried out ChIP from the plants co-infiltrated with either Rep-EGFP or EGFP with ChiLCV. Initially, ChIP was carried out with a GFP-specific antibody in combination with virus-specific primers and desired amplification was obtained in the leaves infiltrated with Rep-EGFP and ChiLCV. However, leaves infiltrated with either ChiLCV or EGFP along with ChiLCV did not indicate the presence of viral DNA (Fig 9E). As expected, mock-treated samples (pCAMBIA2300-infiltrated leaves) failed to show amplification. Samples without antibody served as a negative control whereas sonicated samples were used as the input control for the experiments that confirmed the presence of viral DNA in plants infiltrated with ChiLCV, EGFP and ChiLCV, and Rep-EGFP and ChiLCV (Fig 9E). The binding of Rep to the viral DNA was validated in an electrophoresis mobility shift assay (EMSA) (S9 Fig). Since Rep protein displays DNA-binding activity, we were interested to determine whether Rep can also bind host genomic DNA. To address this issue, the binding of Rep to actin DNA was investigated through ChIP-PCR and the results failed to detect actin DNA in the plants infiltrated with either pCAMBIA2300 and ChiLCV or EGFP with ChiLCV and Rep-EGFP with ChiLCV, whereas input controls showed amplification of actin DNA (Fig 9E).

### Discussion

Euchromatin is modified by several post-translational modifications that can regulate transcription either by recruiting histone post translational modification-specific binding proteins [57], or by directly altering the physical properties of individual nucleosomes [58] and the chromatin fibre [59]. The circular conformation of the ChiLCV genome, its smaller size, and its compact coding and bi-directional transcript organization are remarkably different from the cellular genome, yet during the infection cycle, the ChiLCV ssDNA genome is converted into dsDNA and then assembled into chromatin in the host nuclei. The chromatinized viral genome therefore acts as a potential substrate for histone post-translational and transcriptional machineries but the mechanism of recruitment of the apparatus responsible for histone post-translational modifications on the viral mini-chromosome remains unknown. This study has identified a previously uncharacterized role of Rep and its ability to stimulate / enhance transcription of the viral genome.

In the present study, we detected deposition of H2B-ub and H3-K4me3 on the ChiLCV chromatin. In cellular chromatin, enrichment of H2B-ub and H3-K4me3 at the promoters is known to stimulate transcription by recruiting components of the pre-initiation complex and other transcriptional activators. H2B-ub and H3-K4me3 enrichment on the nucleosome of the ChiLCV promoters aligns with RNA polymerase II occupancy in a manner that is reminiscent of active promoters in eukaryotic chromatin. We hypothesized that active transcription of the ChiLCV genome is regulated by chromatin-based mechanisms similar to the mechanisms in the case of eukaryotic chromatin [60,61]. Although we also detected deposition of H3-K27me3 on viral genome which is considered as repressive epigenetic mark one of the recent studies revealed presence of different forms of geminivirus minichromosome in infected pepper plants [31]. It is possible that during the course of pathogenesis geminivirus minichromosome are present in at least two different populations, transcriptionally active and transcriptionally repressed minichromosome. The transcriptionally active minichromosomes are enriched in deposition of active markers like H3-K4me3 and H2b-ub. Contrary, transcriptionally repressed minichromosomes are enriched in repressor signatures like H3-K27me3. Our study also suggests presence of different type of viral minichromosome in the susceptible *N*. *benthamiana* plants infected with ChiLCV. It is possible those transcriptionally active minichromosomes are enriched in H3-K4me3 and H2B-ub, whereas the minichromosome which are deposited with H3-K27me3 are transcriptionally repressed viral chromatin and are meant for purposes other than transcription.

Previous studies suggested that H3-K4me3 modification is promoted by H2B monoubiquitination that is achieved by the BRE1 homologs HISTONE MONOUBIQUITINATION1 (HUB1) and HUB2 and the RAD6 homologs UBIQUITIN CARRIER PROTEIN1 (UBC1), UBC2, and UBC3 in *Arabidopsis* [36,37,38]. To explain this trans-histone cross-talk, it was postulated that H2B-ub might act as a ‘‘wedge” to non-specifically unfold chromatin for the methyltransferases Set1 and Dot1 to facilitate access to their substrates or, H2B-ub might function as a ‘‘bridge” to directly recruit them [62, 63]. Later on, one of the studies demonstrated that H2B-ub does not act as ‘wedge’ to unfold chromatin non-specifically but functions to maintain nucleosome stability [33]. Two recent studies have implicated Swd2, a Set1-COMPASS complex subunit, as the link between H2B-ub and H3-K4 methylation [64]. Swd2 seems to regulate H3-K79 methylation by recruiting Dot1. As viable Swd2 mutants have a major effect on H3-K4me3 in vivo [64, 65], there may be other regulator(s) mediating H3-K4 mono- and dimethylation.

In the current study, H2B-ub deposition on cellular chromatin was observed to initially increase at 7 dpi following infection of ChiLCV, but then to decrease from 14 and 21 dpi, whereas H3-K4me3 and H3-K27me3 modifications did not show significant difference between mock and infected plants. The expression of sets of genes either promoting or inhibiting virus multiplication are regulated simultaneously. The decreased level of H2B-ub in *N*. *benthamiana* following ChiLCV infection suggests, possibly, down regulation of most of the genes involved in defence against ChiLCV [66, 67]. In the case of adenovirus, the interaction of E1a with human E3 ligase (hBre1) results in inactivation of innate antiviral defense by hampering H2B monoubiquitination, a prerequisite for interferon-mediated response [68]. The enrichment of H3-K4me3 in cellular chromatin suggests its regulation by some other undefined mechanism [69]. This is in line with findings that during the course of myogenic differentiation, H3-K4me3 is acquired and retained on a large number of genes in the absence of detectable H2B-ub [69]. ChiLCV infection reduced the global level of H2B-ub but we observed no altered H3-K4me3, which indicates this dependency may not exist in *N*. *benthamiana* plants. Diminishing levels of H2B-ub was found to correlate with reduced levels of *NbUBC2* and *NbHUB1* transcripts in the ChiLCV-infected plants. But the progressive severity of symptoms implies that the levels of both proteins remain above the threshold level to support viral propagation in a permissive host.

Next, the importance of monoubiquitination of H2B in the pathogenesis of ChiLCV was validated by silencing *NbUBC2* and *NbHUB1* genes in *N*. *benthamiana*. *N*. *benthamiana* has two homologues each of *NbUBC2 and NbHUB1* with redundant function. In *N*. *benthamiana* plants in which either *NbUBC2* or *NbHUB1* were transiently silenced, did not exhibit any phenotypic change from wild type, unlike a previous report of *AtUBC1* and *AtHUB1* transgenic mutants that induced early flowering [41]. Downregulation of *NbUBC2* and *NbHUB1* resulted in decline in the levels of both viral transcript and DNA. Interestingly, downregulation was far more pronounced in *NbUBC2*-silenced plants than the *NbHUB1*-silenced plants. NbUBC2, a ubiquitin-conjugating enzyme (E2), can interact with several cellular E3 ligases that are involved in and regulate many other cellular functions. In yeast, E2 RAD6 also acts together with E3 RAD18 in ubiquitylation [70,71]. Downregulation of *NbUBC2* affects various cellular pathways that may be required for the pathogenesis of ChiLCV. HUB1, an E3 ligase, is known to be involved in the monoubiquitination of H2B (H2B-ub) of cellular chromatin. *NbHUB1* silencing hence affected H2B-ub of chromatin, and this effect explains the low titre of ChiLCV in *NbUBC2*-silenced plants. Earlier reports add to our findings. AtUBC2, for example, was found to rescue the UV-sensitivity of the yeast rad6 mutant [72]. In *Arabidopsis*, the transcript levels of the floral repressor genes FLOWERING LOCUS C (FLC), MADS ASSOCIATED FLOWERING 4 (MAF4) and MAF5 were reported to be reduced in UBC1- and UBC2-silenced plants [41]. In yeast, a RAD6 (a homolog of UBC2) mutant showed decreased H3-K4me3 modifications [62].

Transient silencing of either *NbUBC2* or *NbHUB1* caused a significant reduction in the global cellular deposition of H3-K4me3 and H2B-ub in *N*. *benthamiana*, and the level was further reduced in the silenced plants inoculated with ChiLCV. *NbUBC2* silencing reduced cellular H3-K4me3 and H2B-ub in silenced plants infected with ChiLCV. Down-regulation of *NbUBC2* also decreased cellular histone levels in plants as evidenced by western blotting using anti-H2B specific antibody. It is important to note that along with its own expression, UBC2 is also required for the regulation of H2B gene expression. Since infection of *N*. *benthamiana* by ChiLCV was found to reduce the accumulation of *NbUBC2* transcript, such an infection would in turn lead to a decrease of the deposition of H3-K4me3 and H2B-ub to below the detectable threshold level of western blotting. *NbHUB1* silencing followed by ChiLCV infection induced a comparatively low level of reduction in the deposition of H3-K4me3 and H2B-ub in total cellular post-translational modifications of histone.

Although the current experiments have targeted the silencing of *NbUBC2*, it is possible that these experiments also (inadvertently) silenced other isoform (*NbUBC1;* GenBank accession no. MF374795) because of the considerable sequence identity of *NbUBC2* with the other (S4 Text). Furthermore, qRT-PCR results showed significant down regulation of expression of *NbUBC1* in *NbUBC2*-silenced plants (S3D Fig). In plants, *UBC1* and *UBC2* showed redundant function are responsible for monoubiquitination of H2B [73,74]. Therefore, additional effects due to this silencing of *NbUBC1* cannot be ruled out at this point. Since *NbUBC1* is also involved in H2B ubiquitination, the effects of any downregulation of *NbUBC1* can be considered to be equivalent to the effects of *NbUBC2* downregulation. Monoubiquitination of H2B by *AtUBC2* and *AtHUB1* regulate the expression of several host genes [39,45], and the downregulation of these genes might affect their cellular function in a way that cannot be avoided.

We also analyzed the activation of the ChiLCV promoter in *NbUBC2*- and *NbHUB1*-silenced plants. *NbUBC2*-silenced plants showed a reduction of EGFP-expressing cells in comparison to *NbHUB1*-silenced plants. *NbHUB1*-silenced plants exhibited fewer cells expressing EGFP fluorescence than did wild-type *N*. *benthamiana* plants. Furthermore, silencing of *NbHUB1* and of *NbUBC2* led to less deposition of H2B-ub, which in turn reduced H3-K4me3, leading to low RNA polymerase II occupancy on the viral promoter region. The reduced amount of H3-K4me3 and H2B-ub could also be due to decreased level of viral titer. In general it is considered that even low level of viral titer is sufficient to multiply and produce multiple copies of viral genome in a permissive host. However, in the silenced plants, decreased level of H3-K4me3 and H2B-ub (as evidenced by immunoblotting and ChIP assays) resulted in reduced level of viral transcription, as a result of which the virus failed to produce sufficient amount of viral proteins (above threshold level) and eventually could not overcome the effect of silencing to cause pathogenesis. Together, this information indicates that UBC2 and HUB1 play a pivotal role in the transcription of the geminiviral genome, and hence regulate viral gene expression.

Both ChiLCV Rep and NbHUB1 have nuclear localization sequences and eventually become localized in the nucleus. The major known function of the geminivirus Rep protein is to initiate the viral DNA replication [16,2,15] whereas that of HUB1 is to facilitate monoubiquitination of histone 2B [39]. This function of NbHUB1 is supported by our observation of its interaction with H2B in the nucleus. Furthermore, co-localization of Rep-DsRed and NbHUB1-GFP in the nucleus was confirmed by our confocal microscopy and Pearson’s correlation coefficient results. These results indicated the involvement of both of these proteins in a common cellular function in the nucleus.

NbUBC2 was observed to be localized in the cytoplasm, but concentrated around the nuclear periphery with a low amount in the nucleus. NbUBC2-DsRed fluorescence was observed throughout the cells, consistent with the putative functions of UBC2 with different E3 ligases and cellular proteins. Interestingly, the accumulation of NbUBC2-DsRed in the nucleus increased in the presence of either its cognate partner NbHUB1 or the ChiLCV Rep protein. Although NbUBC2 seems to be localized mainly in the cytoplasm, we found small fractions of this protein in the nucleus, and the ability of Rep to redirect NbUBC2 to the nucleus may stimulate viral transcription, which is thought to occur by the interaction of NbUBC2 with NbHUB1. Previous studies have also suggested re-localization of host protein in presence of viral protein. In one of the studies, poly(A)-binding protein1 (PABP1), a predominantly cytoplasmic protein that is required for initiation of efficient translation, was observed to be partially relocated to the nucleus during herpes simplex virus type 1 (HSV-1) infection [75]. These results suggest that viral proteins can alter the sub-cellular localization of host proteins according to their requirement and functions.

We identified a physical interaction between Rep and the histone post-translational modifications machineries (NbUBC2 and NbHUB1). This result prompted us to hypothesize that Rep recruits NbUBC2 and NbHUB1 for post-translational modifications of histone onto the viral minichromosome. We observed an interaction of the Rep protein with NbHUB1 in the nucleus as evidenced by both BiFC and FRET analyses. FRET analysis has also been used to study the interaction between pre coat protein (encoded by ORF V2) of *Tomato yellow leaf curl virus* (TYLCV) with SGS3 proteins of Arabidopsis and tomato [55]. In previous BiFC assay studies, HUB1-MED21 and HUB1-HUB1 interactions were found to occur in the nucleus [39,45], indicating that the functional compartment of HUB1 is the nucleus. NbHUB1 is able to interact with the full-length Rep protein as well as all three Rep domains under investigation. Our study also suggests NbHUB1 protein residues1-593 aa is essential for its interaction with Rep. Deletion mutants of NbHUB1 containing only residues 1-213aa exhibited β-galactosidase activity in negative control which indicates presence of DNA binding motif. We also found NbUBC2 to interact with the N-terminal region of the Rep protein, i.e. that containing the oligomerization domain (residues 1–180), in both nucleus and cytoplasm. However, NbUBC2 did not interact with Rep_181-361_ domain of the Rep protein. Although the Rep protein interacts with NbUBC2 and NbHUB1, it is not ubiquitinated by the monoubiquitination apparatus present in *N*. *benthamiana*. Furthermore, the possibility of the Rep protein being transported to the nucleus by NbHUB1 and NbUBC2 was ruled out because we did not observe any change of Rep-DsRed localization in either *NbHUB1*-, or *NbUBC2*-silenced plants.

As observed in this present study, oligomerization of Rep in the nucleoplasm has also been shown earlier using BiFC [56]. Previous studies have reported that activities related to site specific nicking and ligation of DNA lies within 1–120 amino acids region of the Rep protein [14,15] during viral genome replication. Our study showed, Rep_1-120_ localized predominantly in the nucleus and nucleolus but suggesting other functions of Rep protein which may be governed by this region. As nucleolus is the centre of rRNA processing and ribosome assembly, it is possible that during the infection cycle, apart from the known roles played by Rep protein, it might be involved in other functions occurring in the nucleolus via its 1–120 amino acids region. Furthermore, the interaction and colocalization study indicate that 1–120 amino acids region of Rep protein region plays pivotal role in the recruitment of NbUBC2 in the nucleus, although other functions of this region can not be excluded. Our observation of localization of Rep_1-180_ in the nucleoplasm (except nucleolus) suggests that Rep may be involved in diverse functions which are governed by different regions of this protein.

The NbUBC2-DsRed signal was more prominent in the nucleus than the cytoplasm in presence of ChiLCV. This result may be due to Rep being one of the early proteins synthesized from the ChiLCV genome and may interact and facilitate the transport of NbUBC2 to the nucleus. Our studies showed that ChiLCV infection did not alter the localization of NbHUB1. Interestingly, unlike the Rep-DsRed alone, Rep-DsRed protein formed, according to our observations, distinct punctate bodies in the nucleus in the presence of ChiLCV. This is the first observation and report of formation of geminivirus Rep protein-induced punctate bodies in infected cells. The sizes of the punctate bodies were highly variable, and these bodies were observed throughout the entire nucleoplasm. The Rep domain 121–180 is indispensable for the formation of punctate bodies. Because of the limited sensitivity of the confocal microscope, we could not clearly identify the exact shape and organization of these specialized structures. Our analysis of FRET experiments suggest that Rep-EGFP did not interact with Rep-DsRed. Furthermore, FRET efficiency of Rep-EGFP and Rep-DsRed indicated significant interaction only in the presence of ChiLCV. Taken together, these results suggest ChiLCV genome induces Rep-Rep interaction that eventually forms Rep-derived punctate bodies. However, prominent punctate bodies with a definite organization were observed when the plants were co-infiltrated with Rep-DsRed, HUB1-GFP and ChiLCV. Here, Rep-DsRed formed well ordered punctate bodies of variable sizes, and most of them were organized as a circular “beads-on-string” -like structure consisting of two to seven Rep-DsRed entities. NbHUB1-GFP fluorescence was observed throughout the nucleus, consistent with HUB1 being involved in the monoubiquitination of H2B of cellular chromatin spread throughout the nucleus. Similarly, the ChiLCV DNA facilitates reorganization of Rep-DsRed, which is otherwise scattered in the nucleus.

The Rep protein can oligomerize, and it binds to the viral DNA during replication. During rolling circle replication, geminiviruses also form concatamer-like structures. In one of the previous reports electron micrographs of negatively stained samples of the cccDNA-containing fraction showed irregular globules of 10–25 nm in diameter and these particles were found to be chromatin-like "beads on a string" [76]. Notably, our results showed the formation of Rep protein derived punctate bodies of variable size one of which was calculated to be 400 nM. The formation and organization of punctate bodies by the Rep protein in the nucleus suggests that the viral genome is either in active replication or transcription. However, in eukaryotes, punctate nuclear bodies appear to be involved in transcriptionally active regions [77]. Previously, based on a transmission electron microscopy (TEM) analysis, it was hypothesized that geminivirus genome is organized as “beads on string” structures consisting of 12 nucleosomes. [78,3,77,79]. However, such a TEM analysis could not predict the nature of viral and host proteins involved in the formation of the “beads-on-string” structure. Our confocal microscopy and ChIP results suggested involvement of viral Rep and host histone proteins in the formation of “beads-on-string” like structure. Further investigations also need to be carried out to determine the other constituents of “beads-on-string”-like structure of Rep proteins and to determine roles of “beads -on-string’ -like structure of Rep protein.

Our results suggested a previously unidentified role for the Rep protein in virus transcription, and we therefore analyzed the effect of the Rep protein on global cellular post-translational modifications of histone. Interestingly, a global increase in neither H2B-ub nor H3-K4me3 was observed when Rep was ectopically expressed in *N*. *benthamiana*. The interaction of Rep with UBC2 and HUB1, along with its ability to bind viral DNA, have led us to hypothesize that Rep may recruit monoubiquitination machinery on the viral promoter region. Furthermore, we were also interested to test the ability of Rep to facilitate recruitment of this apparatus on the host DNA. Our ChIP-PCR results suggested that Rep specifically bound to viral DNA and failed to bind actin DNA. This result suggests that Rep recruits monoubiquitination machinery specifically on the viral genome and not on the host genome. However, we cannot exclude chromatin modification not yet analyzed or histone modifications at a specific set of genes.

Geminivirus Rep is a multi-tasking protein and is involved in replication of the viral genome by both rolling circle replication and recombination-dependant replication. Rep also reprograms cell cycles to facilitate synthesis of viral DNA and can circumvent the RNAi surveillance mechanism of host plants. The small size and limited coding capacity of the geminivirus genome has resulted in a multifarious role of the Rep protein to create a permissive environment within the host cell for its successful propagation. Previous reports indicated an interaction of Rep with histone H3 [80], which is postulated to be involved in displacing nucleosomes from viral DNA to allow access to the replication machinery and/or prevent methylation of H3 lysine 9 [81]. Clearly, the versatile Rep protein has evolved to exploit its ability to interact with the cellular histone post-translational modification apparatus to efficiently stimulate transcription of the viral genome. Recently, the E1A protein encoded by a human adenovirus has been shown to optimally activate early viral transcription and begin the cycle of viral replication [9].

**Fig 10:**
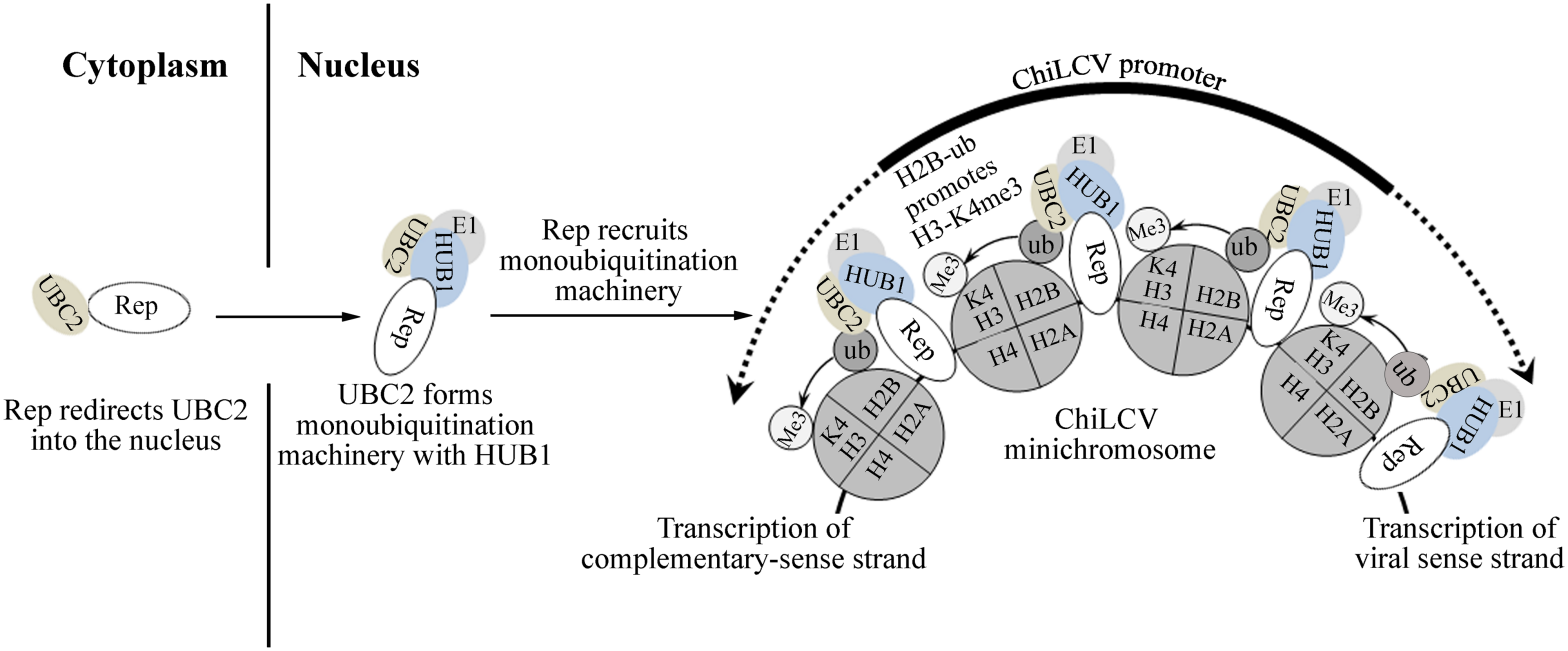
A proposed model for Rep mediated recruitment of histone monoubiquitination machineries on viral promoter and stimulation of geminivirus transcription. In the geminivirus-infected cells, Rep protein redirects NbUBC2 into the nucleus. NbUBC2 in association with NbHUB1 form monoubiquitination machinery in the nucleus. Rep associated with the nucleosomes of viral genome recruits monoubiquitination machinery to ubiquitinate NbH2B on the viral promoter and eventually promotes the trimethylation of H3-K4. Thus Rep-mediated recruitment of the monoubiquitination apparatus stimulates the bi-directional transcription of the viral genome.

To the best of our knowledge, this is the first experimental evidence for the recruitment of histone post-translational modification machineries on chromatin of any plant viral genome. Mechanistically, our study has demonstrated the post-translational modifications of histone on a transcriptionally active viral minichromosome and has uncovered essential roles of Rep for recruitment of histone monoubiquitination machineries and hence for stimulation of geminiviral transcription (Fig 10). Optimal activation of viral transcription may favour synthesis of viral proteins that help to establish pathogenesis of the virus in a permissive host, and such a mechanism undoubtedly acts as an adaptive strategy employed by plant DNA viruses.

## Methods

### Plant growth and inoculation

*Nicotiana benthamiana* plants were grown and maintained under controlled glasshouse conditions of 16 h light and 8 h dark period at 25 ± 2°C with a relative humidity of 60%. *Agrobacterium tumefaciens* strain EHA105 cultures carrying tandem repeat infectious constructs of the viral genome (DNA A like; GenBank accession no. EF190217) and the cognate betasatellite (GenBank accession no. EF190215) were mixed in equal concentrations and *N*. *benthamiana* plants were inoculated at the 5–6 leaves stage following the procedure described by [82].

*A*. *tumefaciens* harbouring only the pCAMBIA2300 vector was used for mock inoculation. All of the inoculated plants were kept in a glasshouse under insect-proof controlled conditions. Symptom severity was assessed following [11].

### Isolation of total plant DNA

The two uppermost leaves from either mock- or virus-inoculated plants were harvested at 7, 14, and 21 days post inoculation (dpi) or as specified elsewhere and total DNA was isolated following the procedure described by [83]. The concentration and quality of the total DNA were assessed with a spectrophotometer (Model ND2000, Nano Drop, Thermo Fisher Scientific, Massachusetts, USA).

### Total RNA isolation, cDNA preparation and qRT-PCR

For isolation of total RNA, the two uppermost leaves were harvested from either virus- or mock-inoculated plants at 7, 14, and 21 dpi using Tri-reagent (Sigma, St Louis, USA) following the manufacturer’s instructions. The quantity of total RNA was analyzed by using a spectrophotometer (Model ND2000, Nano Drop, Thermo Fisher Scientific, Massachusetts,USA). For the synthesis of cDNA, 1μg of DNaseI-treated total RNA was reverse transcribed using oligo(dT_18_) primers and M-MuLV reverse transcriptase (M-MuLV RT RNase H-minus, Thermo Fisher Scientific,Massachusetts USA). The first strand of the cDNA was synthesized by incubating the reaction mixture at 42°C for 1 h and RT was inactivated at 72°C for 10 min. The resultant cDNA was subjected to qRT-PCR using the specific primers.

The qRT-PCR was performed following the procedure described by Kushwaha and colleagues [67]. The experiments were repeated three times with independent biological samples with three technical replicates. Primers for the qRT-PCR were designed using the PRIMER3 software (Applied Biosystem, Massachusetts, USA) and are listed in the S2 Table. PCR reactions were performed in 48-well plates using the SYBR green master mix (Roche, Basel, Switzerland) and carried out on an Eco-Real time PCR (Illumina, California, USA) 40 cycles of the following PCR conditions: 94°C for 10 min, 94°C for 30 s, 55°C for 20 s, 72°C for 20 s. For detection and quantification of viral DNA accumulation, qPCR was performed using 500 ng of total DNA. *N*. *benthamiana* actin (Genbank accession no KA729611) was taken as an endogenous reference for normalization. The threshold cycle (Ct) values of either plant genes or viral DNA were normalized with the Ct value of the internal control and the obtained ΔΔCt values were used to make a relative expression graph using PRISM software (www.graphpad.com). In case of ChIP qPCR, Ct values were further normalized with input values.

### Isolation, cloning and phylogenetic analysis of *NbUBC2* and *NbHUB1*

In the absence of a nucleotide sequence of *N*. *benthamiana UBC2*, the *UBC2* sequence of *N*. *sylvestris* (GenBank accession no XM_009778452) was used to design primers (primer pair no. 8, S2 Table) to amplify *NbUBC2* from *N*. *benthamiana*. The PCR was performed for 28 cycles using *N*. *benthamiana* cDNA as a template and specific primer pairs (initial denaturation at 98°C for 1 min 20 s, followed by 28 cycles of 98°C for 20 s, annealing at 57°C for 30 s and extension at 72°C for 30 s). The PCR-amplified *NbUBC2* was cloned into the pJET1.2 vector (Thermo Fisher Scientific, Massachusetts, USA) and this cloning was confirmed by sequencing using the commercial facility at the University of Delhi South Campus, New Delhi. Likewise for amplification of *NbHUB1*, the tomato *HUB1* sequence (GenBank accession no. XM_010314495) was used to design primers (primer pair no. 12; S2 Table). *NbHUB1* was amplified by using the following PCR program: initial denaturation at 98°C for 1 min 30 s followed by 28 cycles of denaturation at 98°C for 20 s, annealing at 52°C for 1 min and extension at 72°C for 5 min. *NbHUB1* was cloned into the pJET1.2 vector, and was confirmed by sequencing as described above. The phylogenetic trees of *NbHUB1* and *NbUBC2* were generated using the MEGA6 program (http://www.megasoftware.net/) and the exon and intron sequences were mapped using the Arabidopsis homolog on The Arabidopsis Information Resource (TAIR).

### Plasmid constructs and microscopy

DNA sequences for the EGFP and DsRed were amplified by PCR using gene-specific primers (primer pair no. 20 and 1, respectively; S2 Table) and subsequently cloned into the pRT101 vector at the *Bam*HI and *Xba*I sites to obtain pRT101-EGFP and pRT101-DsRed, respectively. The ORF C1 of ChiLCV (coding for the Rep protein) was amplified using specific primers (primer pair no. 19; S2 Table) and was introduced at *Bam*HI-*Kpn*I sites of the pRT101-EGFP vector as well as in pRT101-DsRed vector. Similarly, *NbUBC2* was amplified using primer pair no. 21 (S2 Table) and subsequently cloned into the pRT101-DsRed vector at *Eco*RI-*Kpn*I sites to generate an N-terminal in-frame fusion construct. Rep_1-120_ and Rep_1-180_ were amplified using primer pair no 29 and 30, respectively. Rep_1-120_-DsRed was developed at *Sac*I-*Kpn*I sites and Rep_1-180_-DsRed fusion constructs were made at *Kpn*I-*Bam*HI sites. The constructs were then verified by sequencing. The cassettes carrying CaMV P35S promoter-gene-EGFP, CaMV P35S promoter-gene-DsRed, CaMV P35S promoter-EGFP and CaMV P35S promoter-DsRed were released from the recombinant pRT101 vector and inserted into pCAMBIA2300 at the *Hind*III site. Similarly, Rep_181-361_ was amplified using primer FP 5ʹ GGTACCATGGTTTCTCCTTTTTCTTCTTCTTC 3ʹ and RP 5ʹ GGATCCACGCGTCGACGCCTGGTCCCC 3ʹ and cloned in the pRT101 vector between *Kpn*I and *Bam*HI restriction sites. Above mentioned method was repeated to obtain Rep_181-361_-DsRed construct.

For a co-localization study, *NbHUB1* was amplified using primer pair no. 22 (S2 Table) and cloned into the pCXDG vector [84]. All the constructs were introduced into *A*. *tumefaciens* (strain GV2260) by using the freeze-thaw method and infiltrated onto the lower epidermis of *N*. *benthamiana* plants. For the co-localization study, an equal ratio either of Rep-EGFP and NbUBC2-DsRed or of NbUBC2-DsRed and NbHUB1-GFP or of Rep-DsRed and NbHUB1-GFP were co-infiltrated.

For microscopy studies, the lower epidermis of the infiltrated leaves were peeled off and placed on glass slides. Samples were treated with 4 μg/mL 4',6-diamidino-2-phenylindole (DAPI) for five minutes and washed five times with sterile double-distilled water. These treated samples were mounted in 70% glycerol and observed under a confocal microscope (Eclipse TiE, Nikon, Tokyo, Japan). For analysis of fluorescence, the EGFP constructs were observed under an FITC filter while DsRed constructs were observed under a TRITC filter and DAPI was observed under a DAPI filter. EGFP fusion proteins were imaged with a wavelength of 480 nm excitation, and a 515/30 nm emission filter. Nuclear marker DAPI, when bound to dsDNA, has an absorption maximum at wavelength of 358 nm and emission at 461 nm. The DsRed fusion proteins were imaged at maximum absorption wavelength of 554 nm with emission at 586 nm. The number of cells analyzed under confocal microscope is denoted by ‘n’ in the manuscript. NIS-Element 4.0 software (Nikon, Tokyo, Japan) was used to subtract the background signals from the images of localization, colocalization, and to calculate Pearson’s correlation coefficient of the colocalization.

### Bimolecular fluorescence complementation assays

A bimolecular fluorescence complementation assay (BiFC) was performed using pSPYNE, and pSPYCE vectors that were obtained from the Arabidopsis Biological Resource Center [85]. The ORF C1 of ChiLCV was amplified using primer pair no. 10 (S2 Table) and cloned into the pSPYCE vector at *Kpn*I and *Xho*I sites. *NbHUB1* was amplified using primer pair no. 6 (S2 Table) and cloned into the pSPYNE vector at the *Kpn*I and *Sal*I sites. *NbUBC2* was cloned into the pSPYNE vector at the *Kpn*I and *Bam*HI sites using primer pair no. 28 (S2 Table). The *NbH2B* sequence was amplified using primer pair no. 27 (S2 Table) and cloned into the pSPYCE vector at the *Kpn*I and *Bam*HI sites. The pSCPYCE-Rep, pSPYNE-*NbUBC2* and pSPYNE-*NbHUB1* constructs were transferred into *A*. *tumefaciens* strain GV2260 as previously described. For the BiFC assay, pSPYCE-REP with pSPYNE-*NbUBC2*, pSPYCE-REP with pSPYNE-*NbHUB1*, and pSPYCE-*NbH2B* with pSPYNE-*NbHUB1* were co-infiltrated onto the lower epidermis of *N*. *benthamiana*. In vivo expression was observed under a confocal microscope (Model Eclipse TiE, Nikon, Tokyo, Japan). NIS-Element 4.0 software (Nikon, Tokyo, Japan) was used to subtract the background signals.

### Plasmid constructs and yeast two-hybrid assays

Yeast two-hybrid assays were performed with the GAL4 system according to the manufacturer’s instructions (Clonetech, California, USA). The *UBC* coding sequence of *N*. *benthamiana* (*NbUBC2)* was amplified using primer pair 8 (S2 Table) and cloned into the pGBKT7 vector at the *Nco*I–*Bam*HI sites. Similarly, the full-length coding sequence of *NbHUB1* was amplified by PCR (primer pair no. 12; S2 Table) and subsequently inserted at the *Sal*I site in the pGBKT7 vector whereas DNA sequences corresponding to different domains of *NbHUB1* (NbHub_1-213_) and *NbHUB1* (NbHUB1_1-593_) were amplified (primer pair nos. 13 and 14; S2 Table) and introduced into the pGBKT7 vector at the *Eco*RI-*Sal*I sites. The full-length DNA of ORF C1 was amplified (primer pair no. 2; S2 Table) and introduced into the *Bam*HI-*Sal*I site in the pGADT7 vector (Clontech, California, USA). Nucleotide sequences representing various domains of the Rep protein (Rep_1-120_, Rep_1-180_ and Rep_181-361_) were amplified (primer pairs nos. 3, 4 and 5 respectively; S2 Table) and introduced into the pGADT7 vector at the *Bam*HI-*Sal*I sites. The ORF C2 of ChiLCV was also amplified using primer pair no. 24 (S2 Table) and introduced into the *Nco*I-*Bam*HI sites in the pGADT7 vector.

Lithium acetate mediated transformation of *Saccharomyces cerevisae* strain AH109 was performed following manufacturer’s recommendations (Clontech, CA, USA). Plating was carried out on YPDA plates lacking leucine and tryptophan (2DO). For the β-galactosidase assay, 2–3 colonies were inoculated in 2DO media and kept at 30°C (200 rpm for 24 h). The culture was pelletized and subjected to three cycles of freeze and thaw in liquid nitrogen for one minute each. The pellet was resuspended in 1X PBS buffer containing 0.1% Triton X-100 and X-gal (500 μg/mL). The suspension was vortexed and transferred onto 96-well flat-bottom plates. The plates were incubated at 37°C until a blue color developed.

### Isolation of histone-enriched nuclear proteins

We optimized the protocol for the isolation of histone-enriched protein from the *N*. *benthamiana* plants. Five gram of leaves were harvested and homogenized in liquid nitrogen to isolate intact nuclei. Nuclei were isolated in nuclei isolation buffer containing 20 mM Tris-HCl (pH 7.5), 10 mM KCl, 10 mM MgCl_2_, 6% sucrose, 0.6% Triton X-100, 0.05% β-mercaptoethanol and 1 mM phenylmethylsulfonyl fluoride (PMSF), and a 1:100 dilution of protease inhibitor (SIGMA, St. Louis, USA). The homogenate was filtered through four layers of miracloth and the filtered homogenate was centrifuged at 2800 rpm for 20 min at 4°C. The obtained nuclei pellet was resuspended in the nuclei lysis buffer containing 10 mM Tris HCl pH 7.5, 2 mM EDTA, 0.4 M HCl, 0.05% β-mercaptoethanol, 1 mM PMSF, 0.6% Triton X-100 and 1:100 dilution of protease inhibitor andwas incubated on ice for 4 h with gentle rotation followed by centrifugation at 12000 *g* for 20 min at 4°C. The supernatant was precipitated with 32% trichloroacetic acid and the resulting histone pellet was washed twice with acetone, air dried and stored at -80°C until further use.

### Immunoprecipitation

For immunoprecipitation experiments, 5 g of mock and Rep-EGFP infiltrated *N*. *benthamiana* leaves were harvested and homogenized in nuclei isolation buffer (0.25M sucrose, 15 mM PIPES pH 6.8, 5 mM MgCl_2_, 60 mM KCl, 15 mM NaCl, 1mM CaCl_2_, 0.9% Triton X-100, 1:100 dilution of protease inhibitor. The homogenized sample was centrifuged at 2500 *g* for 20 min at 4°C. The nuclei pellet of the centrifuged sample was taken for total nuclear protein isolation. Total nuclear protein was extracted using nuclei lysis buffer (50 mM HEPES pH 7.5, 150 mM NaCl, 1mM EDTA, 1mM PMSF, 1% SDS, 0.1% Na deoxycholate,1% Triton X-100, 1:100 dilution of protease inhibitor. The lysed nuclear sample was centrifuged at 13000 rpm for 20 min at 4°C. The supernatant from the centrifuged sample was taken in fresh micro centrifuge tube.

The supernatant sample was precleared by adding 50 μL of Agarose A/G beads (sc-2003, Santa Cruz Biotechnology, Dallas, USA) at 4°C for 2 h with gentle rotation, and then centrifuged at 2800 *g* for 2 min after which the supernatant was transferred into a fresh micro centrifuge tube. A mass of 5μg of either anti-GFP (ab290, Abcam, Cambridge, United Kingdom) or Ubiquitin (P4D1, sc-8017, Santa Cruz Biotechnology, Dallas USA) was added into this supernatant and kept on gentle rotation at 4°C for 4 h. This resulting sample was centrifuged at 2800 *g* for 2 min and the resulting beads were washed five times with wash buffer (25 mM Tris-HCl, pH 7.5, 150 mM NaCl, 5% glycerol, 0.05% Nonidet P-40, 2.5 mM EDTA, 1 mM phenylmethylsulfonyl fluoride, and 1:100 volume of complete cocktail of protease inhibitors) and eluted with 2X SDS protein loading bufferafter boiling for 5 min. Immunoblotting was performed using either anti-ub-(1:200, Santa Cruiz Biotechnology, Dallas, USA) or anti-H2B-specific antibodies (ab1790; Abcam, Cambridge, United Kingdom) in 1:5000 dilution.

### Immunoblotting

Total histone protein pellet was dissolved in urea-CHAPS buffer (7 M urea, 2 M thiourea, and 4% CHAPS) and a mass of 10 μg of the protein was separated onto 15% SDS polyacrylamide gel. Subsequently, total histone protein was transferred onto a PVDF (Amersham, Little Chalfont, United Kingdom) membrane, which was processed for western blotting. The membrane was blocked in 5% skimmed milk for 2 h followed by three washings with PBST (1X PBS and 0.05% Tween20). The membrane was incubated in the primary antibody for 2–3 h. After the three washings, the membrane was incubated with anti-rabbit IgG secondary antibody labelled with horseradish peroxidase at a 1:10000 dilution (SIGMA, St. Louis, USA). The immunoblot was developed by applying the ECL method using Amersham ECL Prime Western Blotting Detection Reagent (Amersham, Little Chalfont, United Kingdom). For immunodetection, anti-H2B (ab1790; 1:5000 dilution), anti-H3-K4me3 (ab8580; 1:5000 dilution), anti-ubiquitinated H2B (MM0029; 1:500 dilution) and H3-K27me3 specific antibodies (02–449 Millipore, Massachusetts United State) were used.

### Chromatin immunoprecipitation

A chromatin immunoprecipitation (ChIP) assay was performed following [86] with required modifications. Five grams of leaves from the plants (as described elsewhere) were harvested and crosslinked in buffer containing 0.4 M sucrose, 10 mM Tris–HCl (pH 8), 1 mM PMSF, 1 mM EDTA and 1% formaldehyde by vacuum infiltration for 10–15 min. Crosslinking was stopped by the addition of 2.5 mL of 2 M glycine. The leaves were pat dried and then homogenized in the nuclei isolation buffer (as mentioned above), and the homogenate was filtered through four layers of miracloth and centrifuged at 12000 *g* for 20 min. The resulting white pellet of nuclei was washed twice with nuclei isolation buffer and, finally, was dissolved in 2 mL of nuclei lysis buffer (50 mM HEPES pH 7.5, 150 mM NaCl, 1 mM EDTA, 1% SDS, 0.1% sodium deoxycholate and 1% Triton X-100), followed by centrifugation at 13000 *g* for 20 min. The supernatant was sonicated and centrifuged at 13000 *g* for 10 min at 4°C. A volume of 200 μL of the resulting material was removed and diluted five times. These diluted samples were pre-cleared with 50 μL of salmon sperm DNA/protein A agarose beads for 1 h at 4°C, and then centrifuged at 3500 *g* for 2–4 min at 4°C. The supernatant was removed and the appropriate antibody was added and incubated overnight at 4°C with gentle rotation. A volume of 75 μL of salmon sperm DNA/protein A agarose beads was added into these samples and incubated at 4°C with gentle rotation for 2 h. The resulting samples were then centrifuged at 3500 *g* for 2–4 min at 4°C, and were washed with a low-salt buffer (150 mM NaCl, 20 mM Tris–HCl pH 8, 0.2% SDS, 0.5% Triton X-100 and 2 mM EDTA), then a high-salt buffer (500 mM NaCl, 20 mM Tris–HCl pH 8, 0.2% SDS, 0.5% Triton X-100 and 2 mM EDTA), followed by a LiCl buffer (0.25 M LiCl, 1% sodium deoxycholate, 10 mM Tris–HCl pH 8, 1% NP-40 and 1 mM EDTA) and finally a TE buffer (1 mM EDTA and 10 mM Tris–HCl pH 8). For elution, 250 mL of a freshly prepared elution buffer was added into each of these washed samples and incubated for 15 min at room temperature. These samples were then centrifuged at 3500 *g* for 2–4 min at 4°C followed by addition of 20 μL of 5 M NaCl into the supernatant and was subsequently incubated at 65°C for 4 h to reverse the crosslinking. To remove the histone proteins, a sample of 10 mL of 0.5 M EDTA, 20 μL of 1 M Tris–HCl pH 6.5, and 1 μL proteinase K (20 mg/mL) were added and incubated for 2 h at 45°C. Finally, chromatin was purified by carrying out the phenol-chloroform method and was dissolved in 50 μL of TE buffer. ChIP-PCR was performed using ChiLCV-specific primers (primer pairs 25 or 26; S2 Table) by carrying out the following PCR protocol: initial denaturation at 94°C for 4 min followed by 32 cycles of treatment at 94°C for 30 s, 57°C for 30 s and 72°C for 30 s. For ChIP-PCR of *NbActin*, primer pair no. 18 (S2 Table) was used. The quantitative data of each histone markers was normalized with quantitative data of virus titre in the input control. Furthermore, in the VIGS experiments the normalized quantitative value of pTRV+ ChiLCV was considered as 100% and the per cent decrease of histone markers on the virus promoter was calculated in the *NbHUB1* and *NbUBC2*-silenced plants. The experiments were repeated three times with three biological replicates.

### Fluorescence resonance energy transfer (FRET) microscopy

The FRET experiments were performed by using the acceptor photobleaching method following [54, 55]. The experiments were carried out on Olympus FV1000 microscope (Olympus, Tokyo, Japan). In the experiments EGFP serves as donor and DsRed functions as acceptor molecules. The acceptor was bleached by scanning a region of interest (ROI) at 100% laser power, resulting in photobleaching of at least 90% of the original fluorescence. The pre- and post-bleach images were collected, and ROI fluorescence intensity was measured by using Fluoview 1000 software. Each measurement was conducted on a set of 3 different cells. The percentage of FRET efficiency (*E*_*F*_) was calculated as *E*_*F*_ *= (l*_*n+1*_*-l*_*n*_*) x* 100*/l*_*n+1*_ (where *I*_*n*_and *I*_*n+*1_ are the EGFP intensities at the time points between which the bleaching occurred).

### Virus-induced gene silencing

A tobacco rattle virus (TRV)-based virus-induced gene silencing (VIGS) vector was used in this study. pTRV1 (CD3-1039), pTRV2 (CD3-1040), and pTRV2-*NtPDS* (CD3-1045) vectors [87]—were procured from the Arabidopsis Biological Resource Center (ABRC). The *NbUBC2* sequence (462 bp) was amplified using primer pair no. 7 (S2 Table) and inserted into the pTRV2 vector at *Kpn*I- *Sal*I sites. Similarly, *NbHUB1* was amplified using primer pair no. 11 (S2 Table) and digested with *Eco*RI and *Bam*HI to release a product with a length of 426 bp and this fragment was inserted in the pTRV2 vector at the *Eco*RI site. pTRV2-*NbUBC2* and pTRV2-*NbHUB1* constructs were mobilized in the *A*. *tumefaciens* strain GV2260. The *Agrobacterium* culture carrying pTRV2-*NbHUB1* and pTRV2-*NbUBC2* constructs were grown in LB media supplemented with rifampicin (30 μg/ml), kanamycin (50μg/mL) and carbenicillin (50μg/mL) at 28°C for 24 h. The culture was centrifuged and the pellet was resuspended in buffer containing 10 mM MES, 10 mM MgCl_2_ and 100 μM acetosyringone. The lower leaves of the *N*. *benthamiana* plants were infiltrated with either pTRV-*NbHUB1* (pTRV2-*NbHUB1* + pTRV1) or pTRV-*NbUBC2* (pTRV2-*NbUBC2* + pTRV1) using a needleless syringe and infiltrated plants were kept in a growth chamber maintained at 28°C with 70% relative humidity.

### Southern hybridization

The Southern blotting assay was performed by following Chakraborty et al. [11]. Total plant DNA (8 μg) was separated on 0.8% agarose gels and transferred to a positively charged nylonmembranes (MDI, Ahmedabad, India). Viral DNA was detected by hybridizing the blot with C1-specific radiolabelled (α-^**32**^P dCTP) probes. Virus-specific bands were detected using a phosphorimager analysis system (Typhoon, Amersham, GE Healthcare).

### Detection of siRNAs

Total RNA was isolated from the uppermost leaves (1 g) using Trizol reagent following the manufacturer’s protocol (Sigma, St. Louis, USA). Enrichment of low-molecular-weight RNAs was performed 5% polyethylene glycol 8000–0.5 M NaCl [88]. Twenty micrograms of low-molecular weight RNA was separated from other RNA by using a 15% Tris–borate-EDTA-urea acrylamide gel and transferred onto a nylon Hybond-N+ membrane (Amersham, Little Chalfont, United Kingdom) using a semi-dry electroblotter (Amersham, Little Chalfont, United Kingdom). For the detection of *NbUBC2* and *NbHUB1*, [α-^32^P dCTP]-labelled specific DNA probes were used. The hybridization was carried out overnight at 40°C in hybridization buffer (7% SDS, 0.5 M sodium phosphate, 1 mM EDTA). After two washings with buffer (2X SSC and 0.2% SDS), an image of siRNA was obtained by using a phosphorimager (Typhoon 9210; Amersham, Little Chalfont, United Kingdom).

### Expression and purification of ChiLCV Rep protein

ChiLCV ORF C1 (that codes for Rep protein) was amplified using primer pair no. 9 (S2 Table) and subsequently cloned into the prokaryotic expression pET28a(+) vector at *Bam*HI and *Hind* III. Rep-His was expressed in *Escherichia coli* BL21 (DE3) cells and purified by Ni-NTA affinity chromatography. Briefly, a single colony was cultured at 37°C under constant agitation in 5 mL Luria Broth (LB) medium containing 50 μg/mL kanamycin. The next day, 1% of this culture was used in 1000 mL of fresh medium with appropriate antibiotics. Cells were cultivated at 37°C with shaking until they reached an OD_600_≅ 0.6. Rep-His was induced at 16°C with 0.2 mM isopropyl-b-D-thiogalactopyranoside (IPTG) and the cells were harvested at 6000 rpm for 10 min. The resulting pellet was dissolved in lysis buffer (50 mM Tris-Cl pH 8, 300 mM NaCl, 10% glycerol, 4 mM β- mercaptoethanol, 4 mM PMSF). Cells were lysed with lysozyme (0.5 mg/mL) and sonicated at 25% amplitude with 10 cycles of 10 s on / 50 s off. Sonicated lysate was clarified by centrifugation at 13000 rpm for 40 min and the resulting supernatant was bound to a Ni-NTA column. Washing was done with 50 mM imidazole and recombinant protein was eluted with 300 mM imidazole. The purified Rep-His fractions were denatured at 100°C with Laemmli buffer [89] and run on 12% SDS PAGE.

EGFP was amplified using the primer pair 5ʹFP GGATCCATGGTGAGCAAGGGCGAGGAGC 3ʹ and RP 5ʹ AAGCTTCTTGTACAGCTCGTCCATGCCG 3ʹ and cloned into the pET28a(+) vector at the *Bam*HI and *Hind*III restriction sites. Subsequently, the Rep gene was amplified with specific primers FP 5ʹ GGATCCATGCCTAGGGCTGGGAGATTCAA 3ʹ and RP 5ʹ GGATCCACGCGTCGACGCCTGGTCC 3’ and introduced at the *Bam*HI site of the pET28a-EGFP construct to generate Rep-EGFP_6xHis_ construct. EGFP_6xHis_ and Rep-EGFP_6xHis_ proteins were expressed and purified following the protocols as mentioned above.

### Electrophoresis mobility shift assay

Electrophoresis mobility shift assays (EMSA) were performed using a 120bp (2684–46 nt of circular genome) of the intergenic region (IR) of ChiLCV as a probe. 100ng of double-stranded oligonucleotides were labelled with a Klenow fragment (Thermo Scientific, Massachusetts, USA). EMSA reactions were carried out at 25**°**C for 60 min in 80 μL of buffer (10 mM Hepes-KOH, pH 8.0, 100 mM KCl, 5mM MgCl_**2**_, 30mM NaCl, 1 mM DTT, and 20% glycerol). The EMSA reaction products were loaded onto an 8% polyacrylamide gel and the gel was allowed to run at 120V for 3 h. Further, the gel was dried and kept overnight in radiation-sensitive plates and the signal was detected on a phosphorimager (Typhoon 9200, Amersham, Little Chalfont, UK).

### ATPase assay

The purified EGFP_6xHis_, Rep-EGFP_6xHis_ and Rep_6xHis_ proteins were used to perform ATPase assay following standard method [15]. The proteins at varying concentrations (0.05, 0.1, 0.2,0.3 μg) were incubated with 1:5 dilution of radioactive [γ-^32^P]ATP in a buffer containing 20 mM Tris–HCl (pH 8.0), 1 mM MgCl_2_, 100 mM KCl, 8 mM DTT and 80 μg/ml BSA at 25°C for 15 min and subsequently loaded on the Thin-layer chromatography (Sigma, St Louis, USA) plates and kept in a running buffer (0.5 M LiCl and 1 M HCOOH). Subsequently, the TLC plate was air dried and autoradiographed.

## Supporting information

S1 Fig Infectivity of ChiLCV on *N*. *benthamiana*. (A) qRT-PCR of the viral transcripts in mock-, and virus-inoculated plants at different time points. (B) ChIP-PCR with anti-GFP antibody and ChiLCV promoter specific primer serve as control.
(TIF)

S2 Fig Expression, isolation and phylogenetic analysis of *NbHUB1* and *NbUBC2*. Expression profile of (A) *NbH2B*, (B) *NbUBC2*, and (C) *NbHUB1* in the root, flower, stem and leaves. Expression profile of (D) *NbH2B* (E) *NbUBC2*, and (F) *NbHUB1* in ChiLCV-infected *N*. *benthamiana* at 7, 14, and 21 dpi. The phylogenetic trees of *NbUBC2* (G), and *NbHUB1* (H) were produced using MEGA6 software. Schematic diagrams showing gene organization and domains of NbUBC2 (I) and NbHUB1 (K) proteins that were predicted and generated on the basis of information available on the Sol Genome Network and TAIR. (L) Protein interactome network of AtH2B, AtHUB1 and AtUBC2 generated using STRING database.
(TIF)

S3 Fig *NbUBC2* and *NbHUB1* are indispensable for ChiLCV pathogenesis. (A) Relative expression level of *NbHUB1* in mock, *NbHUB1-*, and *NbUBC2*-silenced plants either in the presence or in the absence of ChiLCV. Student *t*-test was performed to determine the statistical significance of the differences between the mean values using Graphpad Prism software (*** p<0.001 and **p<0.01). (B) The expression profile of *NbUBC2* in mock, *NbHUB1*-, and *NbUBC2*-silenced plants either in the presence or in the absence of ChiLCV. The statistical significance of the differences between the mean values were calculated by performing *t*-test (*** p<0.001 and **p<0.01). (C) Detection of the C2 transcript of ChiLCV by qRT PCR in *NbHUB1-* and *NbUBC2-* silenced plants. (D) qRT PCR study of expression profile of *NbUBC1* and *NbUBC2* in NbUBC2-silenced plants. (*** p<0.001 and **p<0.01).
(TIF)

S4 Fig Localization of EGFP and DsRed in *N*. *benthamiana*. (A) EGFP and DsRed lack proper subcellular sorting signals and therefore the entire cell showed corresponding green (EGFP) or Red (DsRed) fluorescence. (B) Colocalization of fibrillarin-mRFP either NbHUB1-GFP or Rep-EGFP. Fib-mRFP served as nucleolus marker. Confirmation of NbUBC2-EGFP cytoplasmic localization as NbUBC2-EGFP fluorescence merged with the ER tracker signal.
(TIF)

S5 Fig Subcellular localization of NbHUB1, NbUBC2 and Rep proteins in the presence of ChiLCV at 7 dpi. (A) Subcellular localization of NbHUB1-GFP in the presence of ChiLCV. Scale bar = 2 μm. (B) Subcellular localization of NbUBC2-DsRed in the presence of ChiLCV. (C) Formation of punctate bodies by Rep protein in the presence of ChiLCV. (D) Colocalization of Rep and NbUBC2 in the presence of ChiLCV. (E) Constructs expressing NbHUB1-GFP and Rep-DsRed were coinfiltrated along with ChiLCV into *N*. *benthamiana*. Ordered punctate bodies were noticed in the nucleus. (F-H) Rep protein formed irregular shaped punctate bodies (enlarged view) in the nucleus. Scale bar = 2 μm.
(TIF)

S6 Fig Analysis of functional status of GFP-fusion proteins. (A) Rep-EGFP_6XHis_, EGFP_6XHis_ and Rep_6XHis_ proteins were expressed and purified from *E*. *coli* strain BL21 (DE3) cells. (B) Immunoblotting confirmation of Rep-EGFP_6XHis_, EGFP_6XHis_ and Rep_6XHis_ using anti-His antibody. (C) ATPase assay was carried out using 0.05, 0.1, 0.2 and 0.3 μg of proteins for 15 minute and run on the TLC plate. (D) EGFP, NbHUB1-GFP and NbUBC2-EGFPwere expressed in the symptomatic leaves of *N*. *benthamiana* plants infected with ChiLCV. After 21 dpi, immunoblotting was performed using anti-H2B antibody to analyze the level of H2B-ub.
(TIF)

S7 Fig Interaction of Rep with NbHUB1 and NbUBC2. (A) Yeast two-hybrid assay of Rep_181-361_ with NbHUB1 and NbUBC2 on non selective (2DO, -Leu-Trp) and selective (-His/ -Leu /-Trp with 2.5 mM 3-AT) and β-galactosidase (β–gal) assays. (B) Schematic diagram of deletion mutants of NbHUB1, (C) Yeast two-hybrid assay on non selective (2DO, -Leu-Trp) and selective (-His/ -Leu /-Trp with 2.5 mM 3-AT) and β–gal assay of the deletion mutants of NbHUB1 with the Rep protein.
(TIF)

S8 Fig FRET analysis of Rep mutants with NbHUB1 and NbUBC2. (A) FRET microscopy showing pre-bleach and post bleach images with distance and FRET efficiency profiles of each combinations. (B) Graphical representation of FRET efficiency calculated for each combination as indicated.
(TIF)

S9 Fig Purification of the Rep-His recombinant protein and the electrophoretic mobility shift assay (EMSA). (A) Purification of Rep-His protein. ChiLCV Rep protein was expressed as Rep-His recombinant protein in *E*. *coli* BL21. Rep–His was purified following the protocol described in the methods. The size of the recombinant Rep-His protein was observed to be about 45 kDa. (B) Binding of Rep-His with the viral genome by EMSA. The study of the interaction between the viral genome and the Rep-His protein was achieved by carrying out EMSA, following the protocol as mentioned in the Methods section. Rep-His formed a complex with a radiolabelled dsDNA probe of viral DNA and showed a shift in mobility.
(TIF)

S1 Table Annotation and homology analysis of *NbUBC2* and *NbHUB1*. (PDF)

S2 Table Primers sequences used in the study. (PDF)

S1 Text Analysis of the protein sequences of UBC2 from various plants by Clustal W. (PDF)

S2 Text Multiple alignment of protein sequences of HUB1 from various plants by Clustal W. (PDF)

S3 Text Putative nuclear localization signals (NLS) of NbUBC2, NbHUB1 and Rep proteins. (PDF)

S4 Text Sequence alignment of *NbUBC1* and *NbUBC2* (PDF)
