# Correction: The replication initiator protein of a geminivirus interacts with host monoubiquitination machinery and stimulates transcription of the viral genome

**DOI:** 10.1371/journal.ppat.1007281

**Published:** 2018-08-29

**Authors:** Nirbhay Kumar Kushwaha, Mansi, Supriya Chakraborty

There is a mistake in the second author’s name. The correct name is: Mansi. The correct citation is:

Kushwaha NK, Mansi, Chakraborty S (2017) The replication initiator protein of a geminivirus interacts with host monoubiquitination machinery and stimulates transcription of the viral genome. PLoS Pathog 13(8): e1006587. https://doi.org/10.1371/journal.ppat.1006587
