# Correction: The replication initiator protein of a geminivirus interacts with host monoubiquitination machinery and stimulates transcription of the viral genome

**DOI:** 10.1371/journal.ppat.1012030

**Published:** 2024-02-22

**Authors:** Nirbhay Kumar Kushwaha, Mansi Bhardwaj, Supriya Chakraborty

The image for Fig. 7J showing interaction between pGADT7-Rep + pGBKT7-HUB1 and pGADT7-Rep + pGBKT7-UBC2 on 3DO is duplicated on Fig. 7L as pGADT7-Rep_1-120_ + pGBKT7-UBC2 and pGADT7-Rep_1-180_ + pGBKT7-UBC2. The beta-galactosidase assay of pGADT7 + pGBKT7-NbUBC2 is duplicated on the beta-galactosidase assay of pGADT7-Rep1-180 + pGBKT7.

In addition, Fig 1C is duplicated as S1 Fig A. The authors have provided corrected versions of Figs 1 and 7 here.

**Fig 1:**
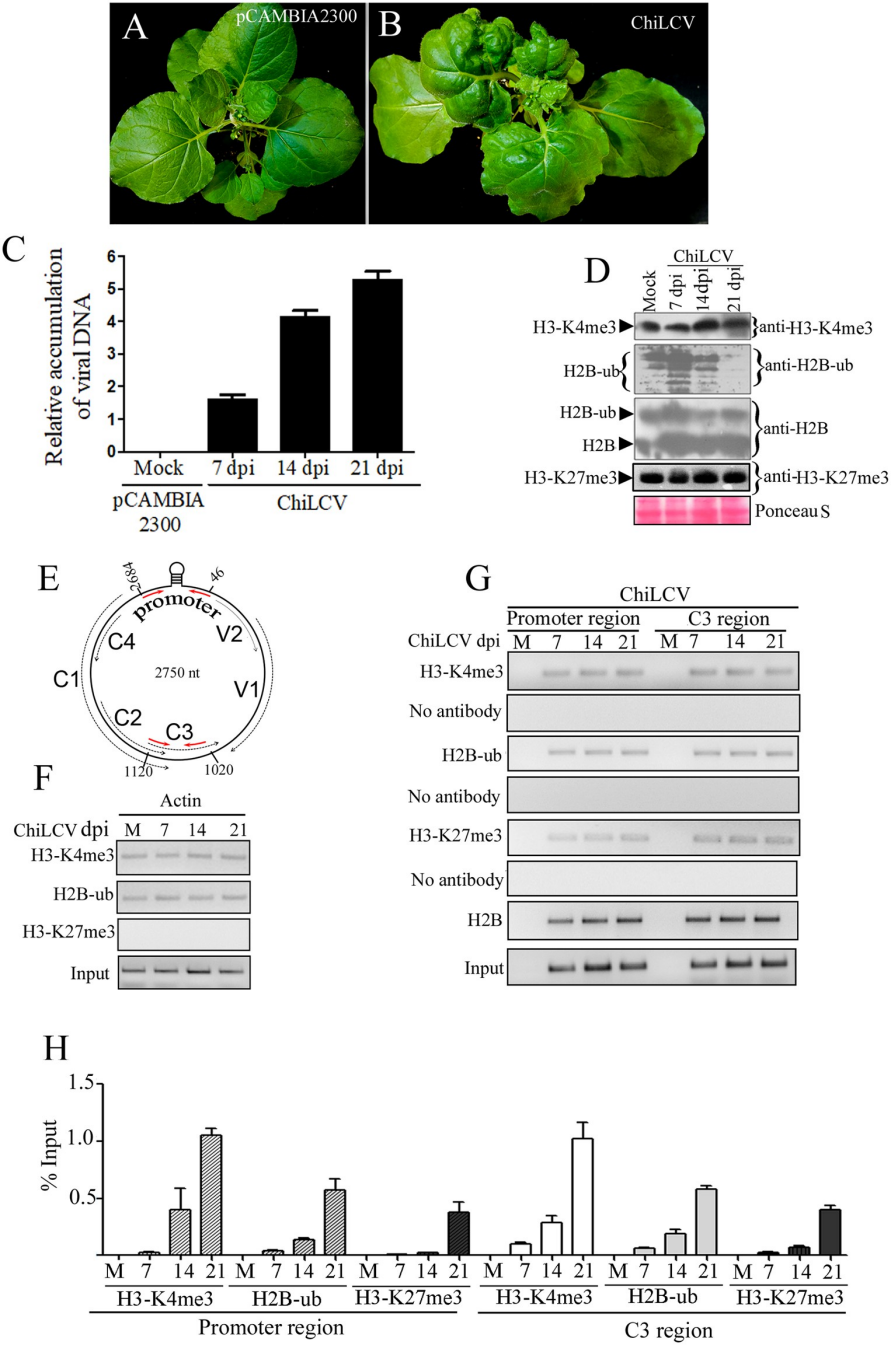
Deposition of H2B-ub and H3-K4me3 on the ChiLCV genome. (A) An *N*. *benthamiana* plant inoculated with the vector pCAMBIA2300. (B) Phenotype of a representative *N*. *benthamiana* plant inoculated with ChiLCV showing typical symptoms of leaf curl disease at 21 days post inoculation (dpi). (C) qPCR of viral DNA accumulation at different dpi. (D) Immunoblot analysis of global cellular H2B, H2B-ub, H3-K4me3 and H3-K27me3 levels in mock-, and virus-inoculated *N*. *benthamiana* plants at 7, 14 and 21 dpi. Immunoblotting was performed using anti-H3-K4me3, anti-H2B-ub, anti-H2B and anti-H3-K27me3 specific antibodies following standard protocol. (E) Schematic diagram of the circular genome of ChiLCV (2750 nt) indicating the relative positions of the viral ORFs and the position of the primers (red arrows) used for chromatin immunoprecipitation in the study. (F) Detection of occupancy of H3-K4me3, H2B-ub and H3-K27me3 on the *Actin* genic region by ChIP-PCR serves as control. (G-H) Detection of H3-K4me3, H2B-ub, H3-K27me3 and H2B on the promoter and C3 region of ChiLCV by ChIP-PCR at different time points following infection using anti-H2B, anti-H2B-ub, anti-H3-K4me3 and H3-K27me3 antibodies and primers specific to the ChiLCV promoter (2684–46 nt) and the C3 region (1020–1120 nt).

**Fig 7:**
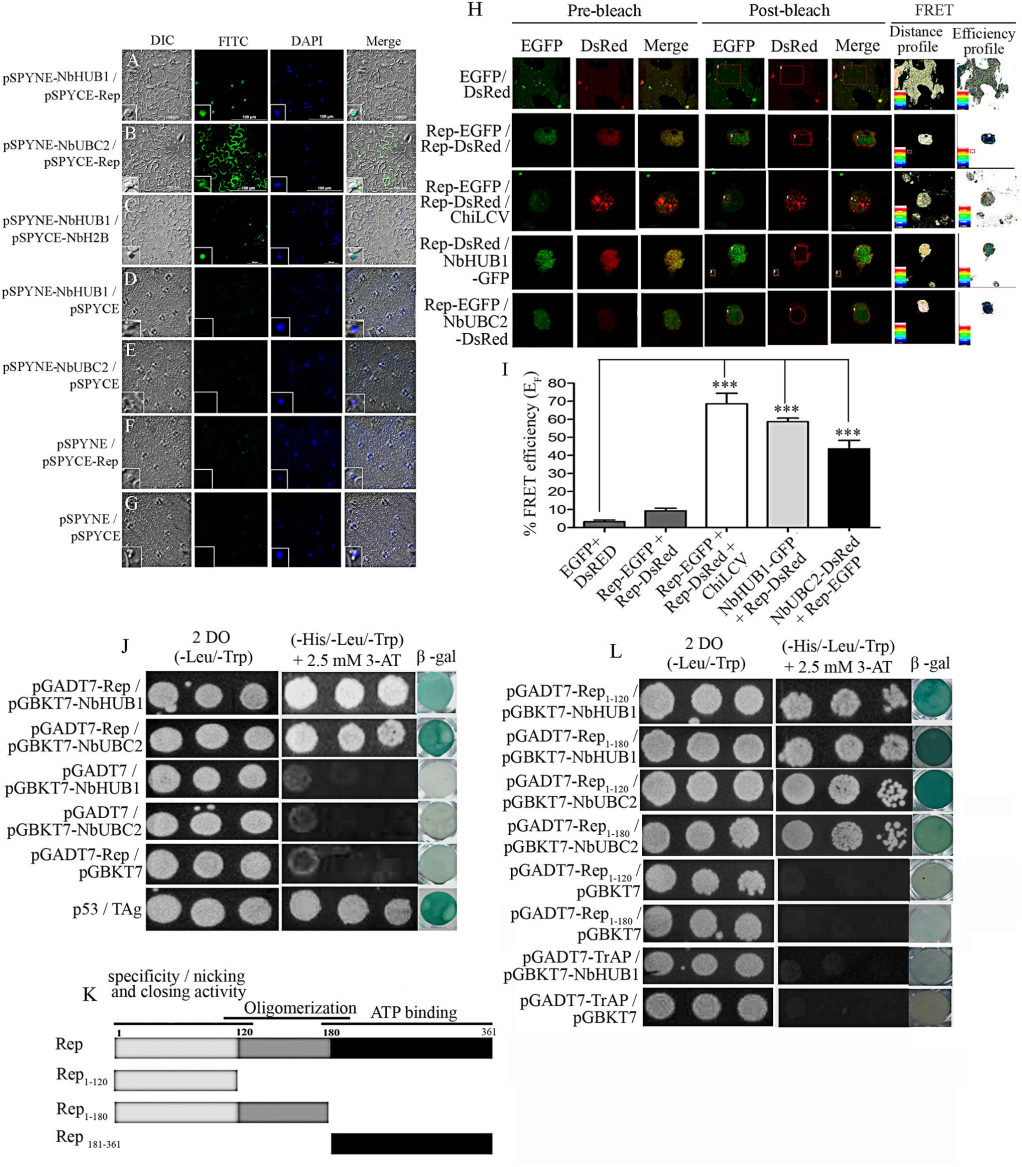
Rep interacts with NbHUB1 and NbUBC2 in vivo. In planta bimolecular fluorescence complementation assays were performed in the lower epidermis of *N*. *benthamiana* leaves (at 5 dpi). NbHUB1 and NbUBC2 were expressed as in-frame fusion with the N-terminal of the YFP protein using the pSPYNE vector. The Rep protein was expressed with C-terminal region of the YFP protein using pSPYCE vector. BiFC assay of interaction of (A) pSPYNE-NbHUB1 and pSPYCE-Rep, (B) pSPYNE-NbUBC2 and pSPYCE-Rep, (C) pSPYNE-NbHUB1 and pSPYCE-NbH2B. (D) pSPYNE-NbHUB1 / pSPYCE, (E) pSPYNE-NbUBC2 / pSPYCE, (F) pSPYCE-Rep / pSPYNE and (G) pSPYNE / pSPYCE serve as control. Scale bar = 100μm. (H) The protein–protein interaction was also monitored by FRET microscopy in the epidermal cells of *N*. *benthamiana* leaves coexpressing EGFP and DsRed, Rep-EGFP and Rep-DsRed, NbHUB1-GFP and Rep-DsRed, Rep-EGFP and NbUBC2-DsRed. Representative acceptor photobleaching images show EGFP (donor) and DsRed (acceptor) channels before and after bleaching. After bleaching, DsRed fluorescence decreases in the bleached are as indicated by rectangles/circles. The FRET profiles showed the proximity between the donor and acceptor molecules and the efficiency of FRET. (I) Graph represents the quantification of FRET efficiency (E_F_) of three independent experiments. (J) Yeast two-hybrid assay of Rep and NbHUB1, Rep and NbUBC2 on non selective media (-Leu / -Trp) and selective media (-His / -Leu / -Trp with 2.5 mM 3-AT). β galactosidase activity were checked for each combination and corresponding negative controls._P_53 and TAg served as positive control. (K) Schematic diagram of deletion mutants of Rep protein used in yeast two-hybrid assays. (L) Yeast two-hybrid and β-galactosidase assays of in vivo interaction between deletion mutants of Rep protein and TrAP with NbHUB1 and NbUBC2.

These inadvertent figure errors do not in any way alter the interpretations or the conclusions drawn in the manuscript.

## References

[1] Kushwaha NK, Bhardwaj M, Chakraborty S (2017) The replication initiator protein of a geminivirus interacts with host monoubiquitination machinery and stimulates transcription of the viral genome. PLoS Pathog 13(8): e1006587. doi: 10.1371/journal.ppat.1006587 28859169 PMC5597257

