# Supplementary figures and images for: The replication initiator protein of a geminivirus interacts with host monoubiquitination machinery and stimulates transcription of the viral genome
Source: PLoS Pathog. 2017 Aug 31;13(8):e1006587. doi: 10.1371/journal.ppat.1006587 (PMC5597257; doi:10.1371/journal.ppat.1006587)

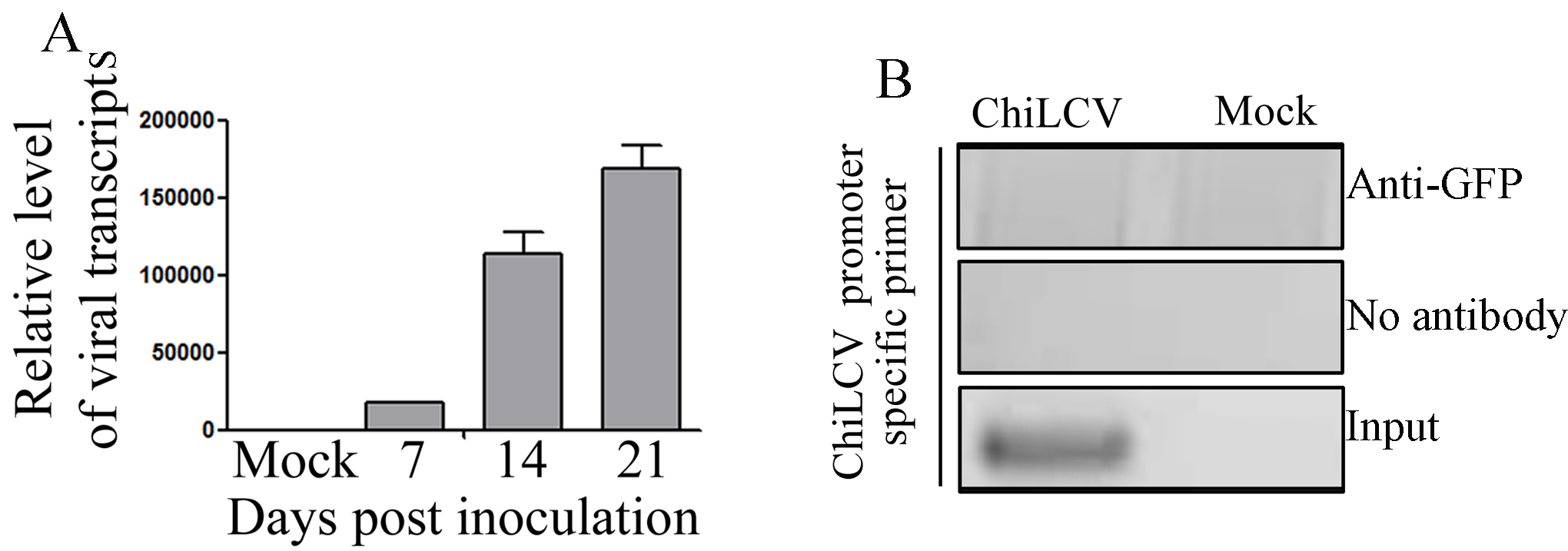

Supplement: S1 Fig — (A) qRT-PCR of the viral transcripts in mock-, and virus-inoculated plants at different time points. (B) ChIP-PCR with anti-GFP antibody and ChiLCV promoter specific primer serve as control. (TIF) [file ppat.1006587.s001.tif]

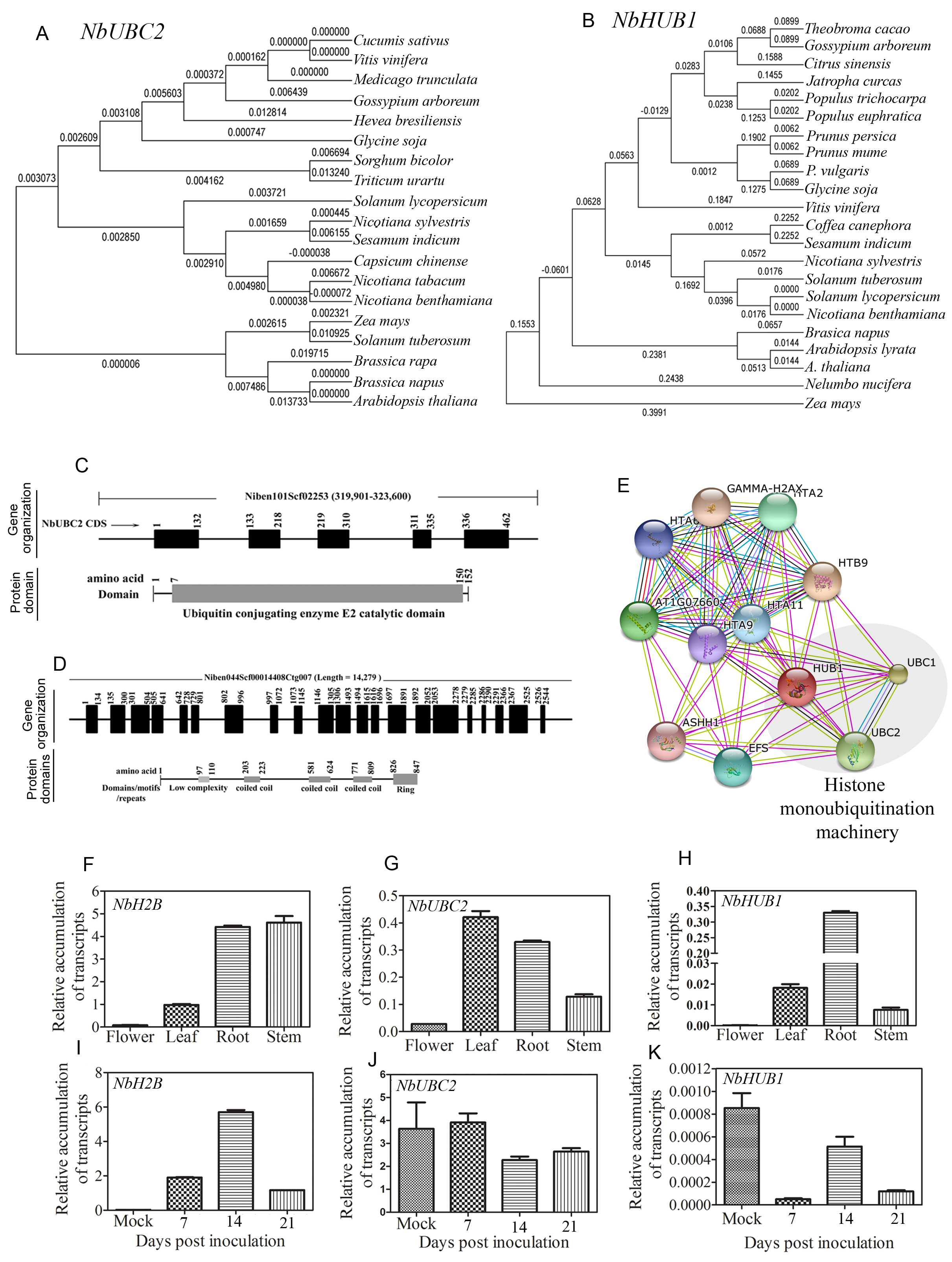

Supplement: S2 Fig — Expression profile of (A) NbH2B, (B) NbUBC2, and (C) NbHUB1 in the root, flower, stem and leaves. Expression profile of (D) NbH2B (E) NbUBC2, and (F) NbHUB1 in ChiLCV-infected N. benthamiana at 7, 14, and 21 dpi. The phylogenetic trees of NbUBC2 (G), and NbHUB1 (H) were produced using MEGA6 software. Schematic diagrams showing gene organization and domains of NbUBC2 (I) and NbHUB1 (K) proteins that were predicted and generated on the basis of information available on the Sol Genome Network and TAIR. (L) Protein interactome network of AtH2B, AtHUB1 and AtUBC2 generated using STRING database. (TIF) [file ppat.1006587.s002.tif]

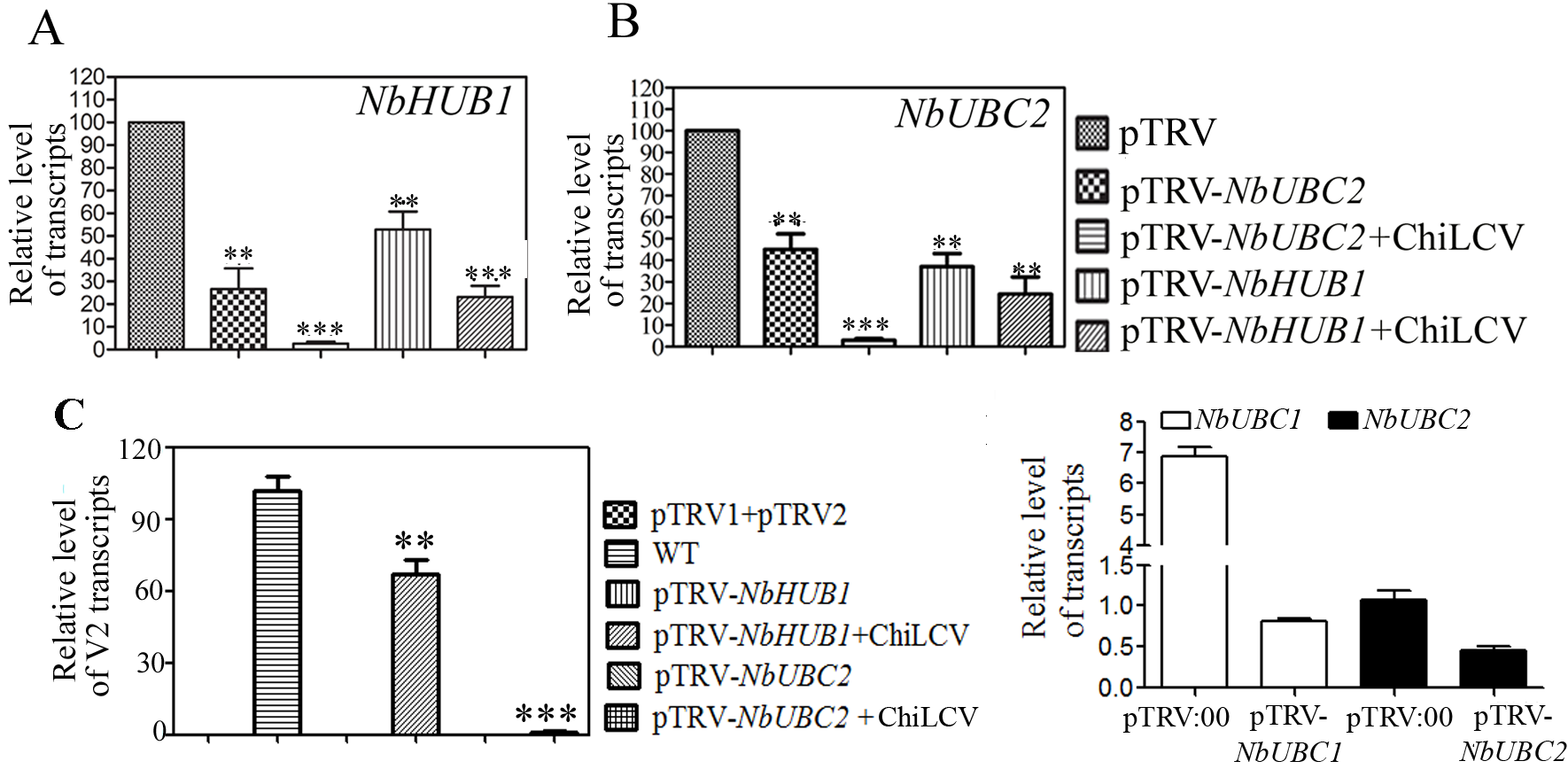

Supplement: S3 Fig — (A) Relative expression level of NbHUB1 in mock, NbHUB1-, and NbUBC2-silenced plants either in the presence or in the absence of ChiLCV. Student t-test was performed to determine the statistical significance of the differences between the mean values using Graphpad Prism software (*** p<0.001 and **p<0.01). (B) The expression profile of NbUBC2 in mock, NbHUB1-, and NbUBC2-silenced plants either in the presence or in the absence of ChiLCV. The statistical significance of the differences between the mean values were calculated by performing t-test (*** p<0.001 and **p<0.01). (C) Detection of the C2 transcript of ChiLCV by qRT PCR in NbHUB1- and NbUBC2- silenced plants. (D) qRT PCR study of expression profile of NbUBC1 and NbUBC2 in NbUBC2-silenced plants. (*** p<0.001 and **p<0.01). (TIF) [file ppat.1006587.s003.tif]

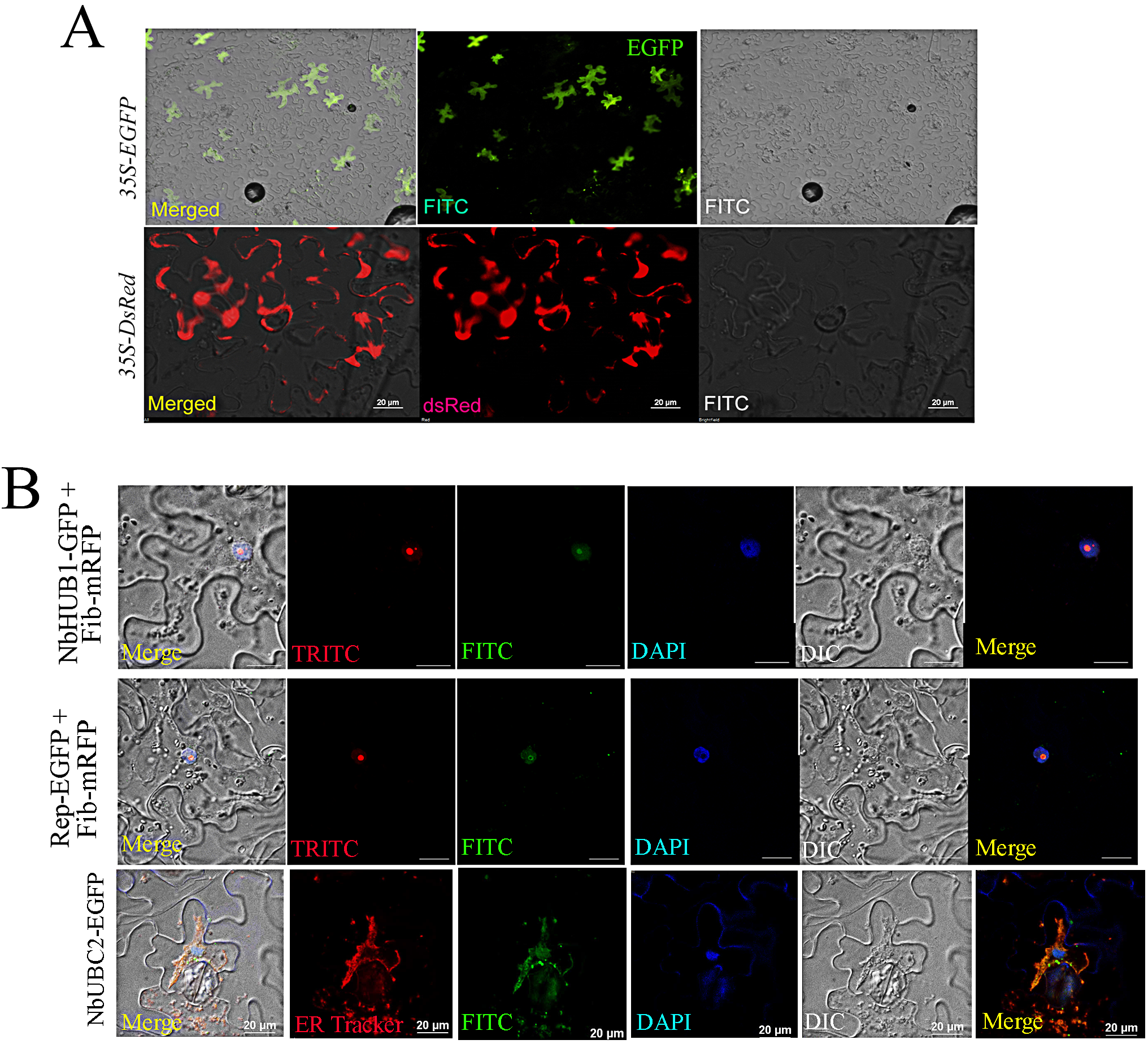

Supplement: S4 Fig — (A) EGFP and DsRed lack proper subcellular sorting signals and therefore the entire cell showed corresponding green (EGFP) or Red (DsRed) fluorescence. (B) Colocalization of fibrillarin-mRFP either NbHUB1-GFP or Rep-EGFP. Fib-mRFP served as nucleolus marker. Confirmation of NbUBC2-EGFP cytoplasmic localization as NbUBC2-EGFP fluorescence merged with the ER tracker signal. (TIF) [file ppat.1006587.s004.tif]

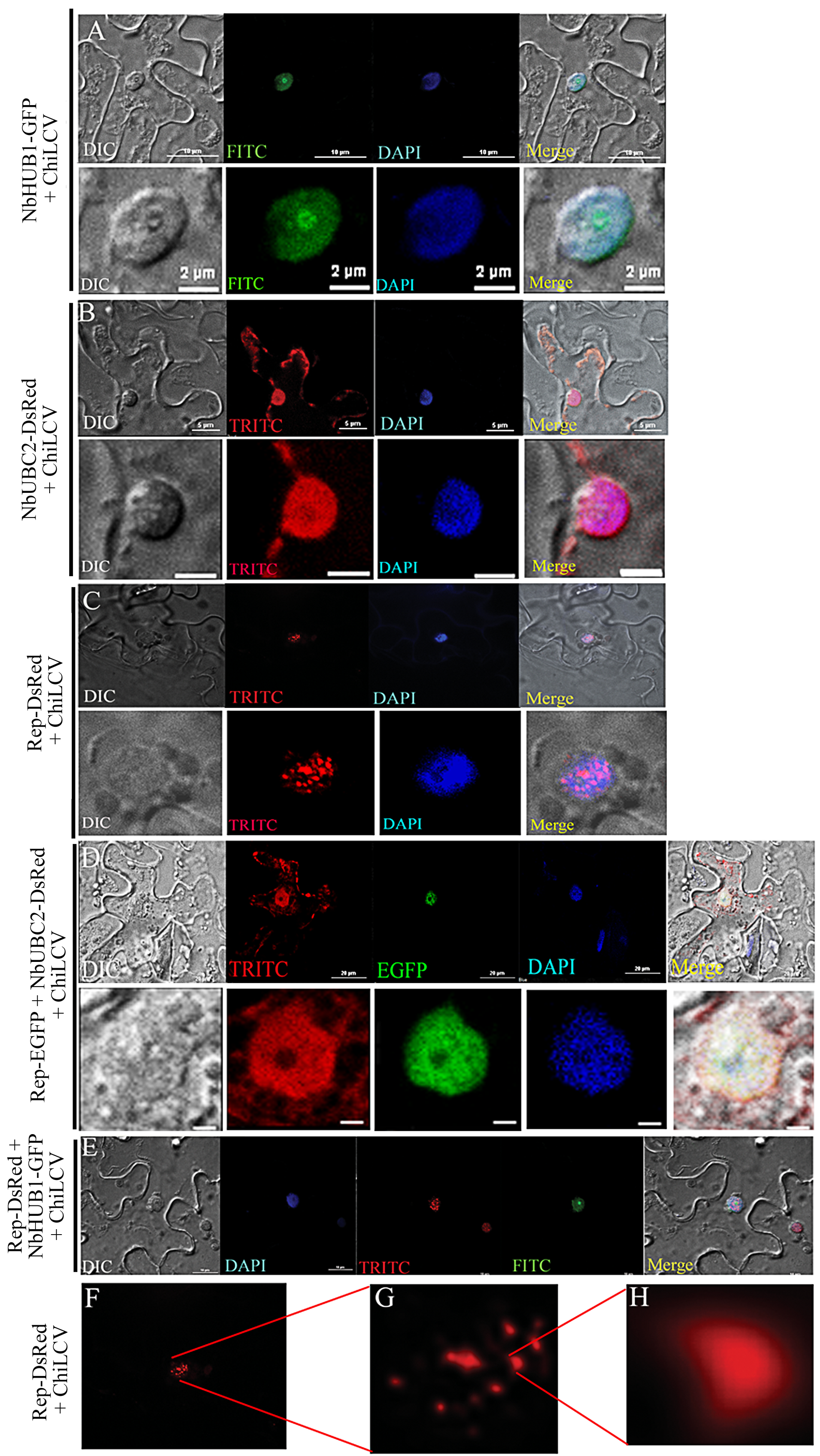

Supplement: S5 Fig — (A) Subcellular localization of NbHUB1-GFP in the presence of ChiLCV. Scale bar = 2 μm. (B) Subcellular localization of NbUBC2-DsRed in the presence of ChiLCV. (C) Formation of punctate bodies by Rep protein in the presence of ChiLCV. (D) Colocalization of Rep and NbUBC2 in the presence of ChiLCV. (E) Constructs expressing NbHUB1-GFP and Rep-DsRed were coinfiltrated along with ChiLCV into N. benthamiana. Ordered punctate bodies were noticed in the nucleus. (F-H) Rep protein formed irregular shaped punctate bodies (enlarged view) in the nucleus. Scale bar = 2 μm. (TIF) [file ppat.1006587.s005.tif]

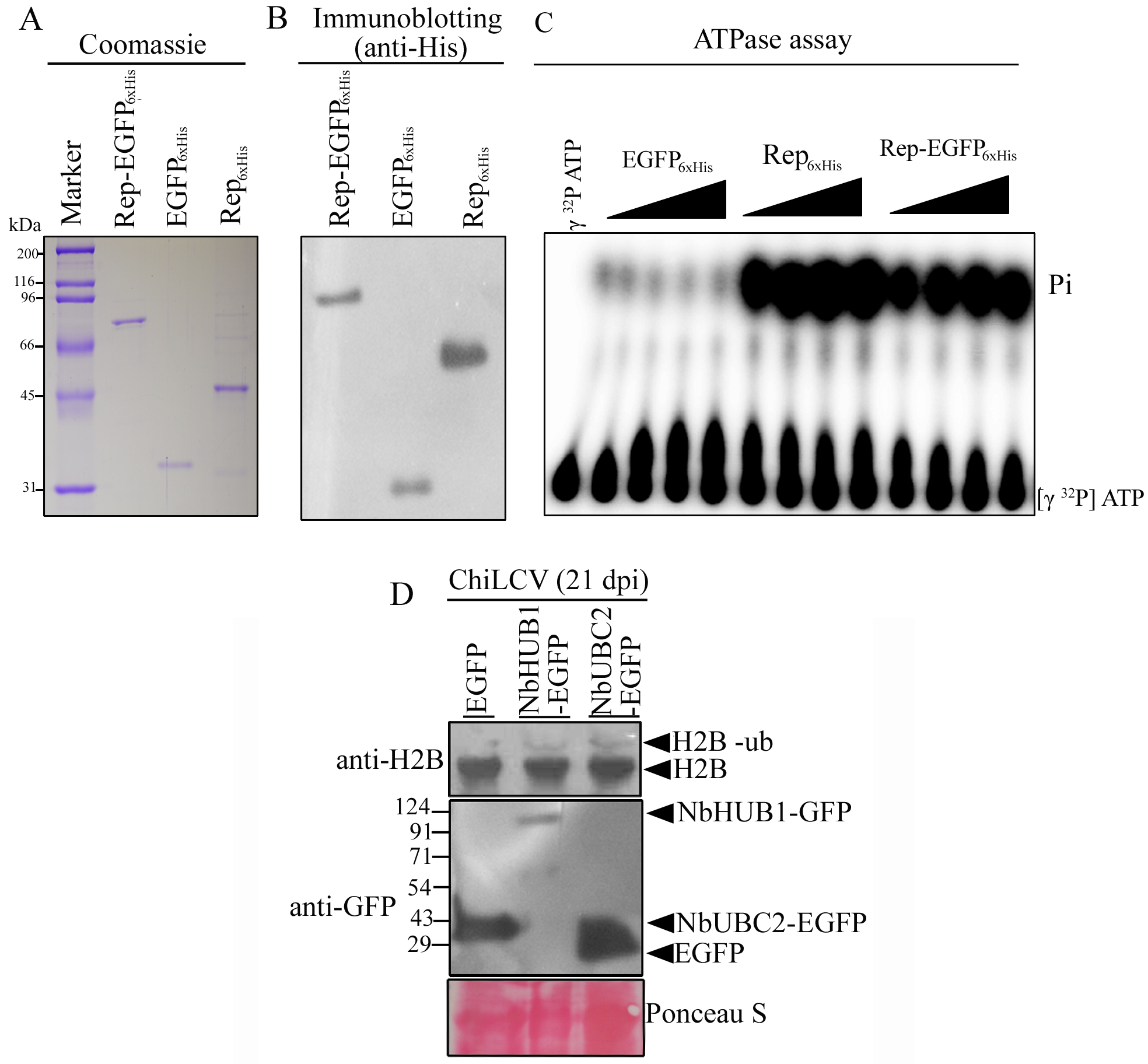

Supplement: S6 Fig — (A) Rep-EGFP6XHis, EGFP6XHis and Rep6XHis proteins were expressed and purified from E. coli strain BL21 (DE3) cells. (B) Immunoblotting confirmation of Rep-EGFP6XHis, EGFP6XHis and Rep6XHis using anti-His antibody. (C) ATPase assay was carried out using 0.05, 0.1, 0.2 and 0.3 μg of proteins for 15 minute and run on the TLC plate. (D) EGFP, NbHUB1-GFP and NbUBC2-EGFPwere expressed in the symptomatic leaves of N. benthamiana plants infected with ChiLCV. After 21 dpi, immunoblotting was performed using anti-H2B antibody to analyze the level of H2B-ub. (TIF) [file ppat.1006587.s006.tif]

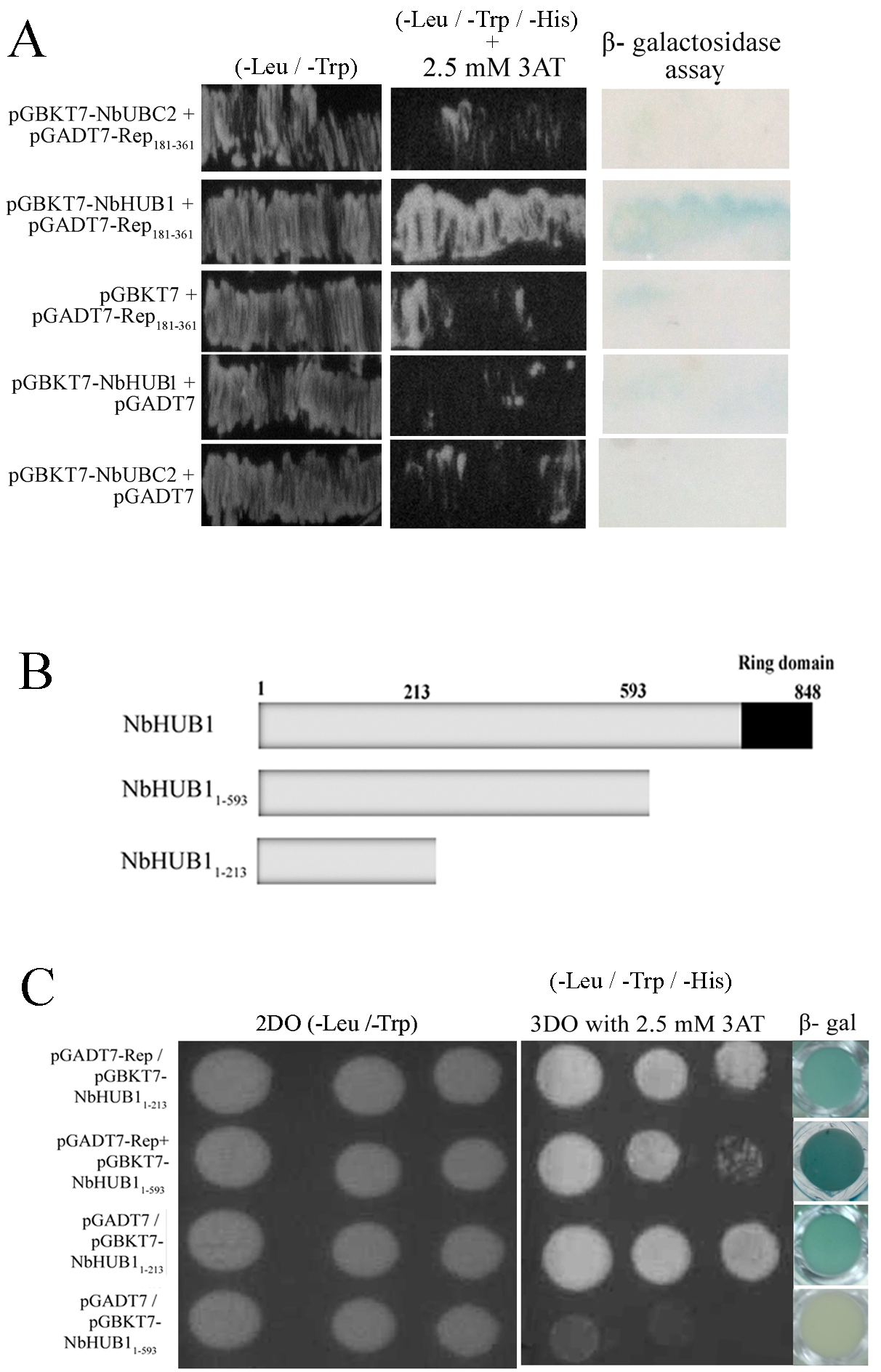

Supplement: S7 Fig — (A) Yeast two-hybrid assay of Rep181-361 with NbHUB1 and NbUBC2 on non selective (2DO, -Leu-Trp) and selective (-His/ -Leu /-Trp with 2.5 mM 3-AT) and β-galactosidase (β–gal) assays. (B) Schematic diagram of deletion mutants of NbHUB1, (C) Yeast two-hybrid assay on non selective (2DO, -Leu-Trp) and selective (-His/ -Leu /-Trp with 2.5 mM 3-AT) and β–gal assay of the deletion mutants of NbHUB1 with the Rep protein. (TIF) [file ppat.1006587.s007.tif]

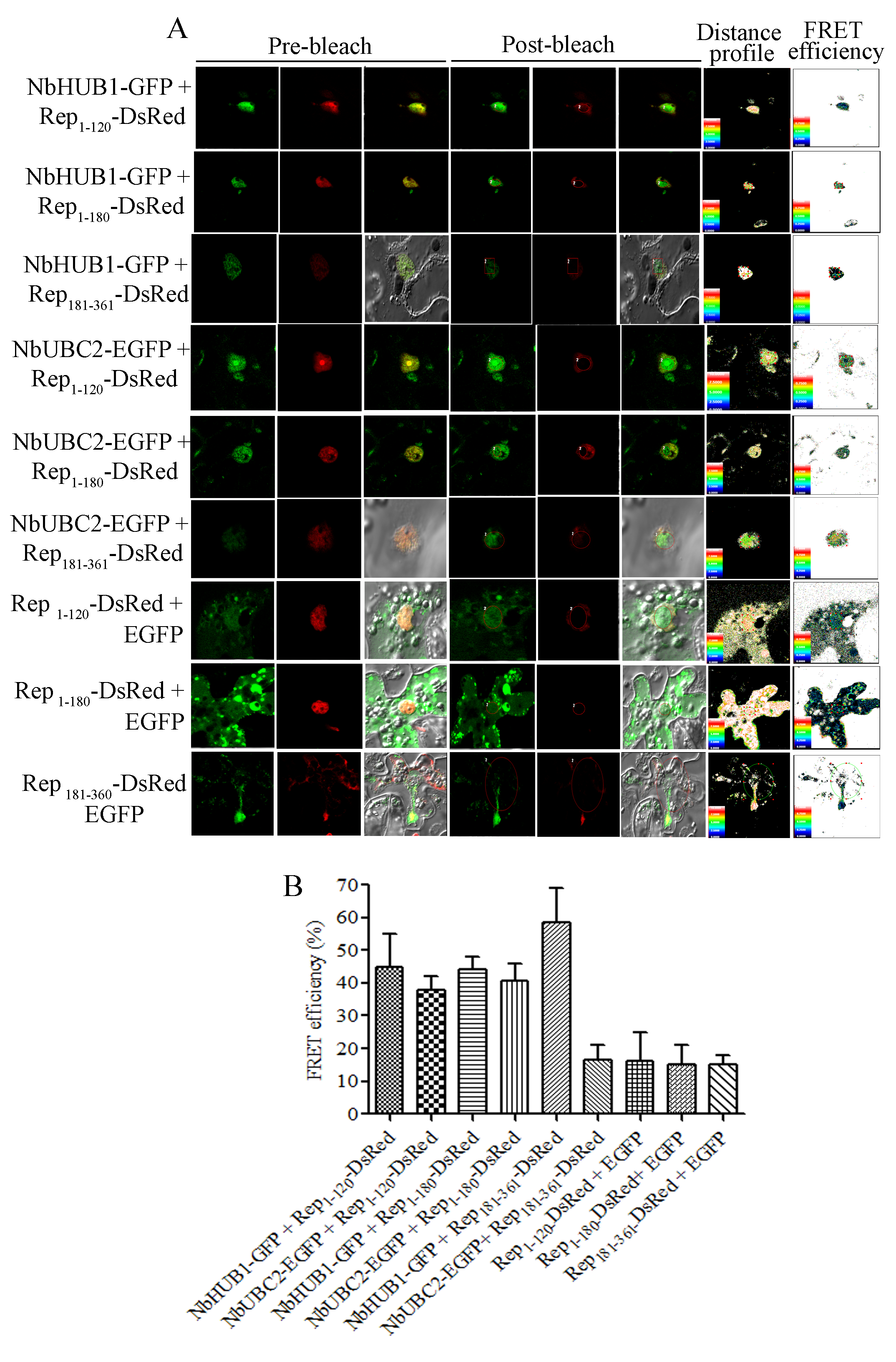

Supplement: S8 Fig — (A) FRET microscopy showing pre-bleach and post bleach images with distance and FRET efficiency profiles of each combinations. (B) Graphical representation of FRET efficiency calculated for each combination as indicated. (TIF) [file ppat.1006587.s008.tif]

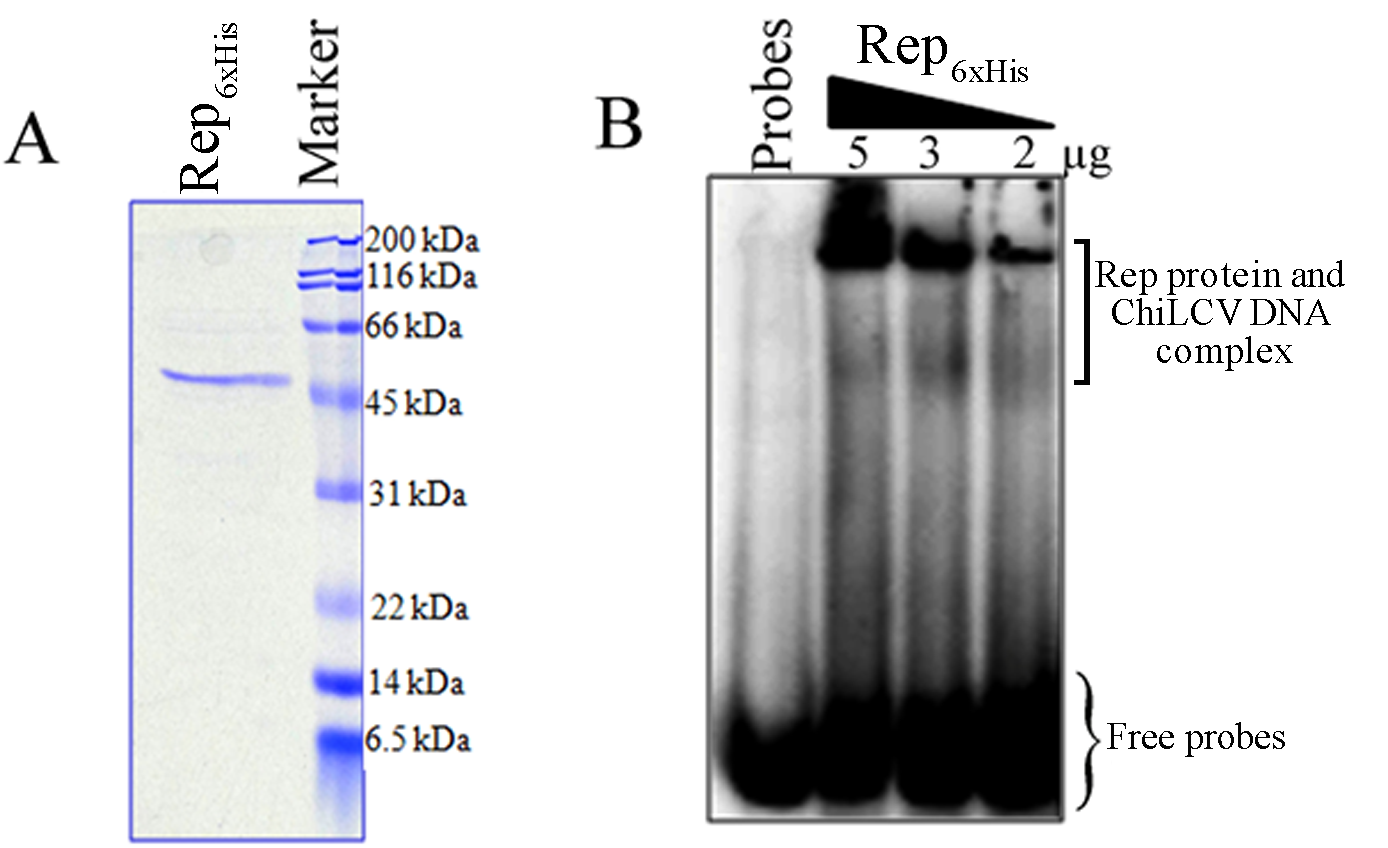

Supplement: S9 Fig — (A) Purification of Rep-His protein. ChiLCV Rep protein was expressed as Rep-His recombinant protein in E. coli BL21. Rep–His was purified following the protocol described in the methods. The size of the recombinant Rep-His protein was observed to be about 45 kDa. (B) Binding of Rep-His with the viral genome by EMSA. The study of the interaction between the viral genome and the Rep-His protein was achieved by carrying out EMSA, following the protocol as mentioned in the Methods section. Rep-His formed a complex with a radiolabelled dsDNA probe of viral DNA and showed a shift in mobility. (TIF) [file ppat.1006587.s009.tif]
