# Supplementary material for: The replication initiator protein of a geminivirus interacts with host monoubiquitination machinery and stimulates transcription of the viral genome
Source: PLoS Pathog. 2017 Aug 31;13(8):e1006587. doi: 10.1371/journal.ppat.1006587 (PMC5597257; doi:10.1371/journal.ppat.1006587)
Supplement: S1 Table — (PDF) [file ppat.1006587.s010.pdf]

Supplemental Table 1: Annotation and homology analysis of NbUBC2 and NbHUB1.

Plant species	Accession no	E-value	identity (%)
NbUBC2			
Nicotiana tomentosiformis	XP_009589859.1	3e-108	99
Solanum lycopersicum	XP_004232235.1	5e-108	99
N. sylvestris	XP_009779442.1	7e-108	99
Solanum tuberosum	XP_006344417.1	8e-108	99
N. tabacum	BAB40311.1	1e-107	99
Zea mays	ACG37110.1	7e-108	99
Oryza sativa	NP_001058973.1	1e-106	97
Sesamum indicum	XP 011085767	2e-107	98
Medicago truncatula	XP 013445765.1	3e-106	97
NbHUB1			
S. lycopersicum	XP_010312797.1	0.0	100
S. tuberosum	XP_006366930.1	0.0	97
N. sylvestris	XP_009798154.1	0.0	88
Vitis vinifera	XP_010661359.1	0.0	64
Populus trichocarpa	XP_006375233.1	0.0	61
Theobroma cacao	XP_007052228.1	0.0	63
Glycine max	XP_006591212.1	0.0	59
