# Supplementary material for: The replication initiator protein of a geminivirus interacts with host monoubiquitination machinery and stimulates transcription of the viral genome
Source: PLoS Pathog. 2017 Aug 31;13(8):e1006587. doi: 10.1371/journal.ppat.1006587 (PMC5597257; doi:10.1371/journal.ppat.1006587)
Supplement: S2 Table — (PDF) [file ppat.1006587.s011.pdf]

Supplemental Table 2: Primers sequences used in the study.

Primer pair No	Primer name	Primer sequences (5′-3′)
1	DsRed	FP GGATCCATGGCCTCCTCCGAGAA<br>RP TCTAGACTATAGGAACAGGTCGT
2	Rep Full	FP GGATCCTAATGCCTAGGGCTGGGAGA<br>RP GAGCTCTCAACGCGTCGACGCCTGGTCC
3	Rep 1-120 (Y2H)	FP GGATCCTAATGCCTAGGGCTGGGAGA<br>RP GAGCTCGCCATCGATTTGGAAAACTCC
4	Rep 1-180 (Y2H)	FP GGATCCTAATGCCTAGGGCTGGGAGA<br>RP GAGCTCATAAACCTCCAACGGAGGTG
5	Rep 181-361 (Y2H)	FP GGATCCATATGTTTCTCCTTTTTCTTCTTCTTC<br>RP GAGCTCACGCGTCGACGCCTGGTCCCC
6	NbHUB1(BiFC)	FP CTCGAGATGGAAAACTTGCTACCGCTTGACAC<br>RP GGTACCTTTGCTCCAAAGCTTGCAGAGC
7	NbUBC2-VIGS-	FP CTCGAGATGATGTCGACTCCAGCGAGGAAGAG<br>RP GGTACCGGATCCCTAGTCGGCAGTCCAGCTTTGTTC
8	NbUBC2(Y2H)	FP CATATGATGTCGACTCCAGCGAGGAAGAGATTGATGAGG<br>RP GGATCCCTAGTCGGCAGTCCAGCTTTGTTCGACAACCTCGCGGAC
9	Rep-His	FP GGATCCATGCCTAGGGCTGGGAGATTCAA<br>RP AAGCTTGACGCGTCGACGCCTGGTCC
10	Rep (BiFC)	FP GGTACCGCCATCGATTTGGAAAACTCC<br>RP GGTACCATAAACCTCCAACGGAGGTG
11	NbHUB1 (VIGS)	FP GAATTCCCCATGGTAATGGAAAACTTGCTACCGCTTGACAC<br>RP GAATTCCTCGGCTTTATTCTTAGCATCGG
12	NbHUB1 Full	FP GTCGACTATGGAAAACTTGCTACCGC<br>RP GTCGACTCAGATATAAACGGCCTTGACATCATTTGC
13	NbHUB1 1-213	FP GAATTCATGGTAATGGAAAACTTGCTACCGCTTGACAC<br>RP GAATTCCTCGGCTTTATTCTTAGCATCGG
14	NbHUB1 1-593	FP GAATTCATGGTAATGGAAAACTTGCTACCGCTTGACAC<br>RP GTCGACAGACTTCAAGACTTCCGAGAGTCTAG
15	NbHUB1 (qRT)	FP ATGGAAAACTTGCTACCGCTTG<br>RP AGACAGAACCATCTTCTGCGC
16	NbUBC2 (qRT)	FP GAAATTCTTGCCGCTTTAACACCCAG<br>RP GCAAATACTCCCATCGGCAT
17	NbH2B(qRT)	FP CAGGTTCATCCTGATATTGGTATCTCT<br>RP AGTCTGAATCTCCCGAGAAGTGA
18	Nbactin (qRT)	FP AGGCTGGATTTGCTGGAGATGATG<br>RP CGTTGTAAAAAGTATGATGCCAAAT
19	REP (Localization)	FP GGTACCATGCCTAGGGCTGGGAGATTCAAT<br>RP GGATCCACGCGTCGACGCCTGGTCC
20	EGFP	FP GGATCCATGGTGAGCAAGGGCGAGGAGC<br>RP TCTAGATTACTTGTACAGCTCGTCCATGCC
21	NbUBC2	FP GAATTCATGTCGACGCCGGCGAGGAAAAGATTG<br>RP GGTACCGTCAGCCGTCCAGCTCTGCTCAAC
22	NbHUB1(pCXDG)	FP AATGGAAAACTTGCTACCGCTTGACAC<br>RP TTTGCTCCAAAGCTTGCAGAGC
23	EMSA CR	FP AATTAGTGTCATCTCAAA<br>RP ACGTGCGTGGTGGGGCCC
24	C2(Y2H)	FP CCATGGATGCGACCTTCATCACCCT<br>RP GGATCCCTAAATACCCTTAAGAAA
25	ChiLCV promoter (ChIP)	FP AATTAGTGTCATCTCAAA<br>RP ACGTGCGTGGTGGGGCCC
26	C3 (ChIP)	FP GTATGCTACCTTAAAGA<br>RP GTATTATGGAATGTATTAGC
27	NbH2B BiFC	FP GGATCCATATGGCACCAAAGGCAGCAGCAGG<br>RP GGTACCGTCGGCGCTGCCAGCGTCCTTTGG
28	NbUBC2 BiFC	FP GGATCCATGATGTCGACTCCAGCGAGGAAGAG<br>RP GGTACCGTCAGCCGTCCAGCTCTGCTCAAC
29	Rep1-120DsRed	FP GAGCTC ATGCCTAGGGCTGGGA<br>RP GGTACC GCCATCGATTTGGAAA
30	Rep1-180DsRed	FP GAGCTC ATGCCTAGGGCTGGGA<br>RP GGATCC ATAAACCTCCAACGGAG
31	C2-qRT	FP CTAAATACCCTTAAGAAATGACCAGTCTG<br>RP ATGCGACCTTCATCATCACCCT
