# Supplementary material for: The replication initiator protein of a geminivirus interacts with host monoubiquitination machinery and stimulates transcription of the viral genome
Source: PLoS Pathog. 2017 Aug 31;13(8):e1006587. doi: 10.1371/journal.ppat.1006587 (PMC5597257; doi:10.1371/journal.ppat.1006587)
Supplement: S1 Text — (PDF) [file ppat.1006587.s012.pdf]

S1 Text. Multiple alignment of protein sequences of UBC2 from various plants by Clustal W.

```
B.rapa              MSTPARKRLMRDFKRLQQDPPAGISGAPHDNNIMLWNAVIFGPDDTPWDGGTFKLSLQFS
Triticum            MSTPSRKRLMRDFKRLQHDPPAGISGAPHDNNIMLWNAVIFGPDDTPWDGGTFKLTLQFT
Sorghum             MSTPARKRLMRDFKRLQQDPPAGISGAPHDNNIMLWNAVIFGPDDTPWDGGTFKLTLQFT
S.tuberosum         MSTPSRKRLMRDFKRLQQDPPAGISGAPYDNNIMLWNAVIFGPDDTPWDGGTFKLTLQFS
Zea                 MSTPARKRLMRDFKRLQQDPPAGISGAPYDNNIMLWNAVIFGPDDTPWDGGTFKLTLQFT
S.lycopersicum      MSTPARKRLMRDFKRLQQDPPAGISGAPYDNNIMLWNAVIFGPDDTPWDGGTFKLTLQFS
Sesamum             MSTPARKRLMRDFKRLQQDPPAGISGAPYDNNIMLWNAVIFGPDDTPWDGGTFKLTLQFT
N.tabacum           MSTPARKRLVRDFKRLQQDPPAGISGAPYDNNIMLWNAVIFGPDDTPWDGGTFKLTLQFS
Capsicum            MSTPARKRLMRDFKRLQQDPPAGISGAPYDNNIMLWNAVIFGPDDTPWDGGTFKLTLQFS
N.benthamiana       MSTPARKRLMRDFKRLQQDPPAGISGAPYDNNIMLWNAVIFGPDDTPWDGGTFKLTLQFS
N.sylvestris        MSTPARKRLMRDFKRLQQDPPAGISGAPYDNNIMLWNAVIFGPDDTPWDGGTFKLTLQFT
B.napus             MSTPARKRLMRDFKRLQQDPPAGISGAPQDNNIMLWNAVIFGPDDTPWDGGTFKLSLQFS
Arabidopsis         MSTPARKRLMRDFKRLQQDPPAGISGAPQDNNIMLWNAVIFGPDDTPWDGGTFKLSLQFS
Hevea               MSTPARKRLMRDFRRLQQDPPAGISGAPQDNNIMLWNAVIFGPDDTPWDGGTFKLTLQFA
Gossypium           MSTPARKRLMRDFKRLQQDPPAGISGAPQDNNIMLWNAVIFGPDDTPWDGGTFKLTLQFT
Glycine             MSTPARKRLMRDFKRLQQDPPAGISGAPQDNNIMLWNAVIFGPDDTPWDGGTFKLTLQFT
Medicago            MSTPARKRLMRDFKRLQQDPPAGISGAPQDNNIMLWNAVIFGPDDTPWDGGTFKLTLQFT
Cucumis             MSTPARKRLMRDFKRLQQDPPAGISGAPQDNNIMLWNAVIFGPDDTPWDGGTFKLTLQFT
Vitis               MSTPARKRLMRDFKRLQQDPPAGISGAPQDNNIMLWNAVIFGPDDTPWDGGTFKLTLQFT
                    ****:****:***:***:********** *************************:***:

B.rapa              EDYPNKPPTVRFVSRMFHPNIYADGSICLDILQNQWSPIYDVAAILTSIQSLLCDPNPNS
Triticum            EDYPNKPPTVRFVSRMFHPNIYADGSICLDILQNQWSPIYDVAAILTSIQSLLCDPNPNS
Sorghum             EDYPNKPPTVRFVSRMFHPNIYADGSICLDILQNQWSPIYDVAAILTSIQSLLCDPNPNS
S.tuberosum         EDYPNKPPTVRFVSRMFHPNIYADGSICLDILQNQWSPIYDVAAILTSIQSLLCDPNPNS
Zea                 EDYPNKPPTVRFVSRMFHPNIYADGSICLDILQNQWSPIYDVAAILTSIQSLLCDPNPNS
S.lycopersicum      EDYPNKPPTVRFVSRMFHPNIYADGSICLDILQNQWSPIYDVAAILTSIQSLLCDPNPNS
Sesamum             EDYPNKPPTVRFISRMFHPNIYADGSICLDILQNQWSPIYDVAAILTSIQSLLCDPNPNS
N.tabacum           EDYPNKPPTVRFISRMFHPNIYADGSICLDILQNQWSPIYDVAAILTSIQSLLCDPNPNS
Capsicum            EDYPNKPPTVRFISRMFHPNIYADGSICLDILQNQWSPIYDVAAILTSIQSLLCDPNPNS
N.benthamiana       EDYPNKPPTVRFISRMFHPNIYADGSICLDILQNQWSPIYDVAAILTSIQSLLCDPNPNS
N.sylvestris        EDYPNKPPTVRFISRMFHPNIYADGSICLDILQNQWSPIYDVAAILTSIQSLLCDPNPNS
B.napus             EDYPNKPPTVRFVSRMFHPNIYADGSICLDILQNQWSPIYDVAAILTSIQSLLCDPNPNS
Arabidopsis         EDYPNKPPTVRFVSRMFHPNIYADGSICLDILQNQWSPIYDVAAILTSIQSLLCDPNPNS
Hevea               EDYPNKPPTVRFVSRMFHPNIYADGSICLDILQNQWSPIYDVAAILTSIQSLLCDPNPNS
Gossypium           EDYPNKPPTVRFISRMFHPNIYADGSICLDILQNQWSPIYDVAAILTSIQSLLCDPNPNS
Glycine             EDYPNKPPTVRFVSRMFHPNIYADGSICLDILQNQWSPIYDVAAILTSIQSLLCDPNPNS
Medicago            EDYPNKPPTVRFVSRMFHPNIYADGSICLDILQNQWSPIYDVAAILTSIQSLLCDPNPNS
Cucumis             EDYPNKPPTVRFVSRMFHPNIYADGSICLDILQNQWSPIYDVAAILTSIQSLLCDPNPNS
Vitis               EDYPNKPPTVRFVSRMFHPNIYADGSICLDILQNQWSPIYDVAAILTSIQSLLCDPNPNS
                    ************:**********************'''''********************

B.rapa              PANSEAARMYSENKREYNRKVRDVVEQSWTAD
Triticum            PANSEAARMFSENKREYNRKVREIVEQSWTAD
Sorghum             PANSEAARLFSENKREYNRKVREIVEQSWTAD
S.tuberosum         PANSEAARMFSENKREYNRKVREVVEQSWTAD
Zea                 PANSEAARMFSENKREYNRKVREVVEQSWTAD
S.lycopersicum      PANSEAARMFSENKREYNRKVREIVEQSWTAD
Sesamum             PANSEAARLFSENKREYNRKVREIVEQSWTAD
N.tabacum           PANSEAARMFSENKREYNRKVREIVEQSWTAD
Capsicum            PANSEAARMFSENKREYNRKVREIVEQSWTAD
N.benthamiana       PANSEAARMFSENKREYNRKVREIVEQSWTAD
N.sylvestris        PANSEAARMFSENKREYNRKVREIVEQSWTAD
B.napus             PANSEAARMFSESKREYNRRVREVVEQSWTAD
Arabidopsis         PANSEAARMFSESKREYNRRVREVVEQSWTAD
Hevea               PANSEAARMFSENKREYNRRVREIVEQSWTAD
Gossypium           PANSEAARMFSENKREYNRRVREIVEQSWTAD
Glycine             PANSEAARMFSENKREYNRKVREIVEQSWTAD
Medicago            PANSEAARMFSENKREYNRRVREIVEQSWTAD
Cucumis             PANSEAARMFSENKREYNRRVREIVEQSWTAD
Vitis               PANSEAARMFSENKREYNRRVREIVEQSWTAD
                    ********::**.******:**::********
```
