# Supplementary material for: The replication initiator protein of a geminivirus interacts with host monoubiquitination machinery and stimulates transcription of the viral genome
Source: PLoS Pathog. 2017 Aug 31;13(8):e1006587. doi: 10.1371/journal.ppat.1006587 (PMC5597257; doi:10.1371/journal.ppat.1006587)
Supplement: S3 Text — (PDF) [file ppat.1006587.s014.pdf]

S3 Text : Putative nuclear localization signals (NLS) of NbUBC2, NbHUB1 and Rep proteins.

Predicted putative NLS in NbHUB1 protein

```
MENLLPLDTAVLLHQNQKLSQKLEAQKIEIAVLEEKFTELRDKQKPYDNT  50
LSAIQKSWEELVGELEICSTRTEDPIRHGNASNDQSCAEDGSVYACDDSF  100
LSLLLQTGATGSSSDVNTQTEYEQKKMDDQKIVKIFRNIVSTVDNVRQMK  150
DKLCAAVLEVLPEDGSCLQKSLSDLHVGVKNLIQTINELHLKHRSLAGAL  200
QNHRDTDAKNKAELKCLRGELEKTIAHLDESNRKLAILKAEKDAAKGVLF  250
PVLNLGNKHSANDKARDKQRDMQDMESTLKEYLDQSSFRLFELKRLHEER  300
IDILKQLSNLQNKLKNLKAICSSQPYILVKDQLAKAKEDLSLYQSLYEKL  350
QVEKDNLSWREKEMNLKNDITDVFRRSSTIADSRIAWLEKEMQKHMQERN  400
MIEGKLEEASREPGRKEIIAEFKKLVSSFPETMGDMQNQLSNYKETASDV  450
HSLRTDVQSLSSILDRKSKEIEALSAKSASQVTEMLKLQAVVNDLKESDM  500
HLKLILEMYTRESAFSRDVFEARSSEYRAWARVQSLKTSLDEHNLEVRVK  550
SAIEAEADSQQKLGAAEAEIAELRQKLDASKRERSRLSEVLKSKHEETEA  600
YLSEIETIGQAYDDMQAQNQQLFQQITERDDYNIKLVLEGVRARQQRDCL  650
AWESQITERAVEDANTMVSSYEMKAAKIDDQLRGCSDLIQKLAEDRGQNS  700
LALENTQKRFLDVRKSSQQLRETLEEWQSKIDEVRVDLAQLQIELEKERF  750
ERKRAEEDVEALRRKTSRLRSHIEGSSVIEKLQQKLREYKEILNCSICFD  800
RRKEVVLAKCYHLFCNPCIQKIVETRHRKCPVCSASFGANDVKAVYI     847
```

Predicted bipartite NLS		
Pos.	Sequence	Score
289	RLFELKRLHEERIDILKQLSNLQNKLKNLKAI	7.4
704	ENTQKRFLDVRKSSQQLRETLEEWQSKIDEV	5.1

NbUBC2 lacks putative NLS sequence

```
MSTPARKRLMRDFKRLQQDPPAGISGAPYDNNIMLWNAVIFGPDDTPWDG  50
GTFKLTLQFSEDYPNKPPTVRFISRMFHPNIYADGSICLDILQNQWSPIY  100
DVAAILTSIQSLLCDPNPNSPANSEAARMFSENKREYNRKVREIVEQSWT  150
AD                                                  152
```

Predicted putative NLS sequence in ChiLCV Rep protein

```
MPRAGRFNINAKNYFLTYPNCSLTKEEALSQLQNLETPVNKLFIRVCREL  50
HENREPHLHVLVQFEGKYQCTNNRFFDLISPTRSAHFHPNIQRAKSSSDV  100
KAYVEKDGDFIDFGVFQIDGRSARGGCQSANDAYAEAINSGSKSSALNIL  150
REKAPKDYVLQFHNLNANLDRIFTPPLEVYVSPFSSSSFDQVPEELEEWA  200
AENVLGAAARPLRPMSIVIEGESRTGKTIWARSLGPHNYLCGHLDLSPKV  250
YSNDAWYNVIDDVDPHYLKHFKEFMGAQRDWQSNTKYGKPVQIKGGIPAI  300
FLCNPGPNSSYKEFLDEEKNSALRNWALKNATFITLEGPLYSGSNQSAAQ  350
ASQEGDQASTR                                         361
```

Predicted bipartite NLS		
Pos.	Sequence	Score
245	DLSPKVYSNDAWYNVIDDVDPHYLKHFKE	5
