# Supplementary material for: The replication initiator protein of a geminivirus interacts with host monoubiquitination machinery and stimulates transcription of the viral genome
Source: PLoS Pathog. 2017 Aug 31;13(8):e1006587. doi: 10.1371/journal.ppat.1006587 (PMC5597257; doi:10.1371/journal.ppat.1006587)
Supplement: S4 Text — (PDF) [file ppat.1006587.s015.pdf]

S4 Text : Multiple alignment of nucleotide sequences of *NbUBC1* and *NbUBC2 of N. benthamiana* by ClustalW (www.ebi.ac.uk/Tools/msa/**clustalw**2).

```
NbUBC2      ATGTCGACTCCAGCGAGGAAGAGATTGATGAGGGATTTTAAGCGGTTACAGCAAGATCCC
NbUBC1      ATGTCGACTCCAGCTAGAAAGAGGTTGATGAGAGATTTCAAGAGGTTGCAGCAGGACCCT
            ************** ** ***** ******** ***** *** **** ***** ** **

NbUBC2      CCGGCCGGCATCAGTGGAGCTCCGTATGACAACAATATAATGCTATGGAATGCAGTCATT
NbUBC1      CCTGCTGGTATTAGTGGTGCACCTCAAGACAACAACATTATGCTTTGGAATGCCGTGATA
            ** ** ** ** ***** ** **  * ******** ** ***** ******** ** **

NbUBC2      TTCGGCCCTGATGATACTCCCTGGGATGGAGGTACATTTAAGCTGACACTTCAATTCTCA
NbUBC1      TTTGGTCCTGATGACACTCCTTGGGATGGTGGTACGTTCAAGCTGACTCTTCAATTCTCT
            ** ** ******** ***** ******** ***** ** ******** ***********

NbUBC2      GAGGACTATCCAAACAAACCACCAACTGTGCGGTTTATTTCCAGAATGTTCCACCCAAAT
NbUBC1      GAGGATTACCCCAATAAGCCACCAACAGTGCGGTTTGTTTCTCGCATGTTTCATCCTAAC
            ***** ** ** ** ** ******** ********* ****  * ***** ** ** **

NbUBC2      ATTTACGCTGATGGAAGTATTTGCTTAGACATCCTGCAAAATCAGTGGAGTCCCATATAT
NbUBC1      ATTTATGCAGATGGAAGTATATGTTTGGATATTCTTCAAAATCAGTGGAGTCCAATATAT
            ***** ** *********** ** ** ** ** ** ***************** ******

NbUBC2      GATGTAGCTGCTATACTGACTTCAATCCAGTCTTTGCTCTGTGATCCAAATCCTAACTCG
NbUBC1      GATGTTGCAGCTATACTTACATCCATTCAGTCATTGCTGTGCGATCCCAACCCCAATTCA
            ***** ** ******** ** ** ** ***** ***** ** ***** ** ** ** **

NbUBC2      CCAGCAAATTCAGAAGCAGCACGCATGTTCAGTGAGAACAAGCGTGAATACAACAGGAAG
NbUBC1      CCTGCAAATTCGGAAGCAGCTCGGATGTTCAGCGAGAATAAAAGGGATTACAACCGCAGA
            ** ******** ******** ** ******** ***** **  * ** ****** * *

NbUBC2      GTGCGCGAGGTTGTCGAACAAAGCTGGACTGCCGACTAG------------
NbUBC1      GTTAGAGAAGTTGTGGAGCAGAGCTGGACTGCTGACGGATCCATCTGCTGA
            **  * ** ***** ** ** *********** ***
```
