# Supplementary material for: The replication initiator protein of a geminivirus interacts with host monoubiquitination machinery and stimulates transcription of the viral genome
Source: PLoS Pathog. 2017 Aug 31;13(8):e1006587. doi: 10.1371/journal.ppat.1006587 (PMC5597257; doi:10.1371/journal.ppat.1006587)

S2 Text. Multiple alignment of protein sequences of HUB1 from various plants by Clustal W.

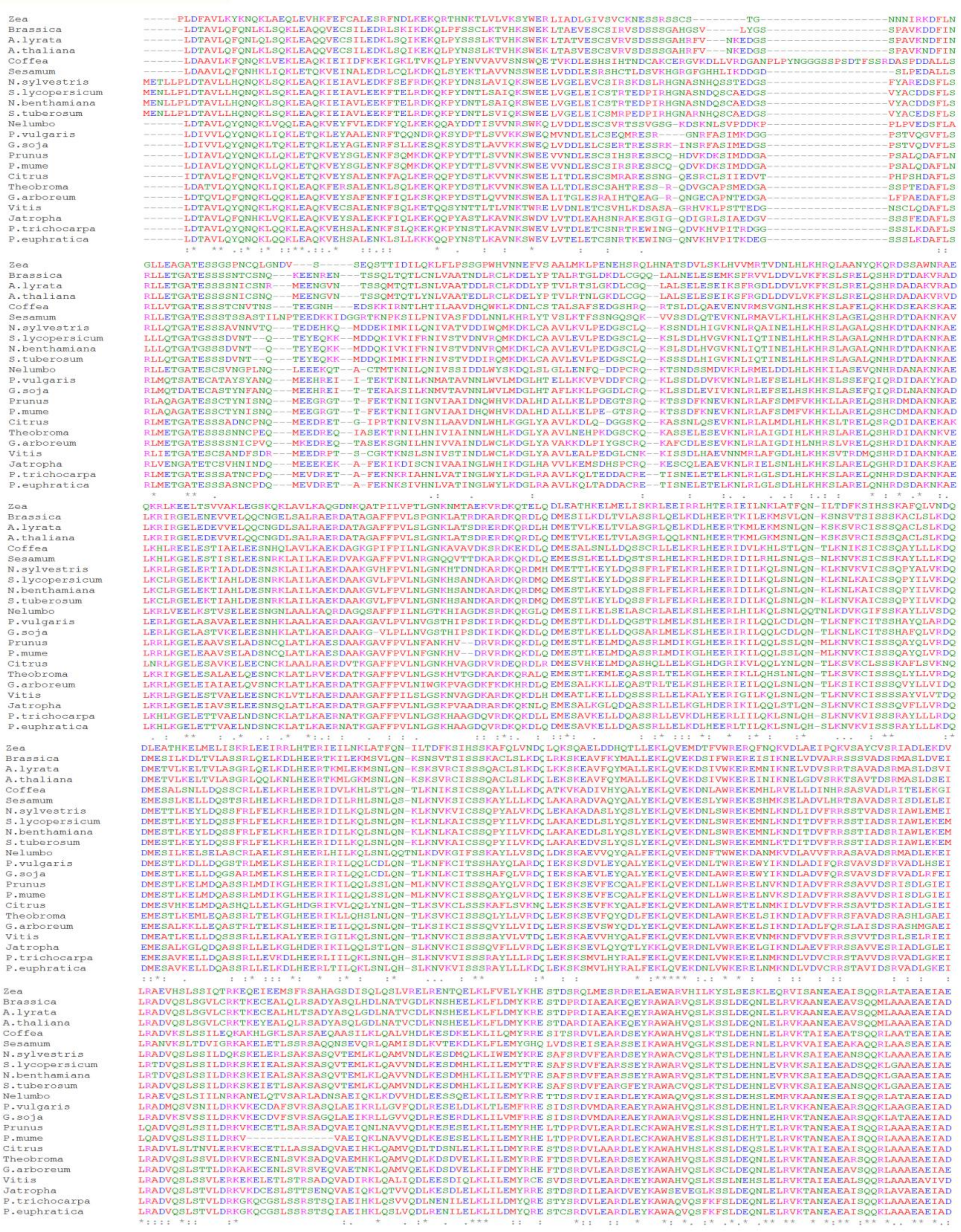

Supplement: S2 Text — (PDF) [file ppat.1006587.s013.pdf]
